# Estimating Local Chlamydia Incidence and Prevalence Using Surveillance Data

**DOI:** 10.1097/EDE.0000000000000655

**Published:** 2017-06-01

**Authors:** Joanna Lewis, Peter J. White

**Affiliations:** From the a NIHR Health Protection Research Unit in Modelling Methodology and MRC Centre for Outbreak Analysis and Modelling, Department of Infectious Disease Epidemiology, Imperial College London, London, United Kingdom; and b Modelling and Economics Unit, National Infection Service, Public Health England, London, United Kingdom.

## Abstract

**Background:**

Understanding patterns of chlamydia prevalence is important for addressing inequalities and planning cost-effective control programs. Population-based surveys are costly; the best data for England come from the Natsal national surveys, which are only available once per decade, and are nationally representative but not powered to compare prevalence in different localities. Prevalence estimates at finer spatial and temporal scales are required.

**Methods:**

We present a method for estimating local prevalence by modeling the infection, testing, and treatment processes. Prior probability distributions for parameters describing natural history and treatment-seeking behavior are informed by the literature or calibrated using national prevalence estimates. By combining them with surveillance data on numbers of chlamydia tests and diagnoses, we obtain estimates of local screening rates, incidence, and prevalence. We illustrate the method by application to data from England.

**Results:**

Our estimates of national prevalence by age group agree with the Natsal-3 survey. They could be improved by additional information on the number of diagnosed cases that were asymptomatic. There is substantial local-level variation in prevalence, with more infection in deprived areas. Incidence in each sex is strongly correlated with prevalence in the other. Importantly, we find that positivity (the proportion of tests which were positive) does not provide a reliable proxy for prevalence.

**Conclusion:**

This approach provides local chlamydia prevalence estimates from surveillance data, which could inform analyses to identify and understand local prevalence patterns and assess local programs. Estimates could be more accurate if surveillance systems recorded additional information, including on symptoms. See video abstract at, http://links.lww.com/EDE/B211.

## Abstract 2

Supplemental Digital Content is available in the text.

The treatment and control of sexually transmitted bacterial infections (bacterial STIs) forms an important component of national sexual health programs because infection can have serious sequelae including pelvic inflammatory disease in women, leading to ectopic pregnancy and infertility.^1^ The most common bacterial STI in Britain is chlamydia, with overall prevalence in 2010–2012 estimated at 1.5% in women and 1.1% in men aged 16–44 years and reporting at least one lifetime sexual partner.^2^ Infection levels vary with age and sex, however, with the highest prevalence, 4.7%, in 18- to 19-year-old women.

Screening and/or widespread opportunistic testing programs for chlamydia have been introduced in countries including England, Australia, the USA, and the Netherlands. National screening programs present opportunities for collecting large quantities of surveillance data, which is valuable but not always straightforward to interpret. For example, if the numbers of chlamydia tests and diagnoses are both higher in one area than another, does this indicate a difference in prevalence or simply more infections identified in one area because of the larger number of tests? If an unusually large proportion of tests in a particular area are positive, is local prevalence higher or were a greater proportion of tests sought in response to symptomatic infections as opposed to being asymptomatic “screens”?

In this article, we present a model-based framework for estimating local chlamydia prevalence using the numbers of tests and diagnoses reported through surveillance systems for a single year. Our method provides direct prevalence estimates as an alternative to test positivity, which is the best proxy currently available but has no simple relationship to prevalence and is affected by the risk profile of those tested.^3^ In addition to positivity, we use information on testing rates from local surveillance, and natural history parameters from the literature. The model considers the contributions of two processes toward the observed numbers of tests and diagnoses: treatment seeking by symptomatic cases and opportunistic testing of individuals without symptoms. We illustrate the method by applying it to publicly available surveillance/program data from England, first at the national, aggregate level by age group, as a validation check, and then broken down by local authority (LA; political administrative units in England) to illustrate how local prevalence can be estimated.

## METHODS

### Terminology

We use the term *coverage* for the number of chlamydia tests recorded in a given year, divided by the population size. (Here, *population* refers to the population of interest: sexually active men and women in England ages between 15 and 24 years.) Subtly different definitions of coverage are used by different authors, but ours is chosen for consistency with the English National Chlamydia Screening Programme (NCSP). By this definition, coverage can in theory be greater than 100% if enough members of the population are tested repeatedly over the year. The *annual diagnoses per capita* is the number of positive tests recorded in a given year, divided by the population size. The *positivity* is the number of positive tests recorded, divided by the total number of tests. *Prevalence* is the proportion of the population who are infected at a given moment, and *incidence* is the number of new infections per unit time, per uninfected member of the population. We describe only quantities with units of time^−1^ as “rates,” although what we term “coverage” and “annual diagnoses per capita” are often described this way elsewhere.

*Screening* describes testing not linked to symptoms: for example, as part of a regular health check, through sexual health outreach services, or offered opportunistically during clinic attendance for another reason.

We define three categories of individual: *uninfected*, (infected-)*symptomatic*, and (infected-)*asymptomatic*. *Nonsymptomatic* individuals are all those not experiencing symptoms, whether because they are uninfected or because they are infected but asymptomatic.

Our terminology is summarized in Table 1.

**TABLE 1.**
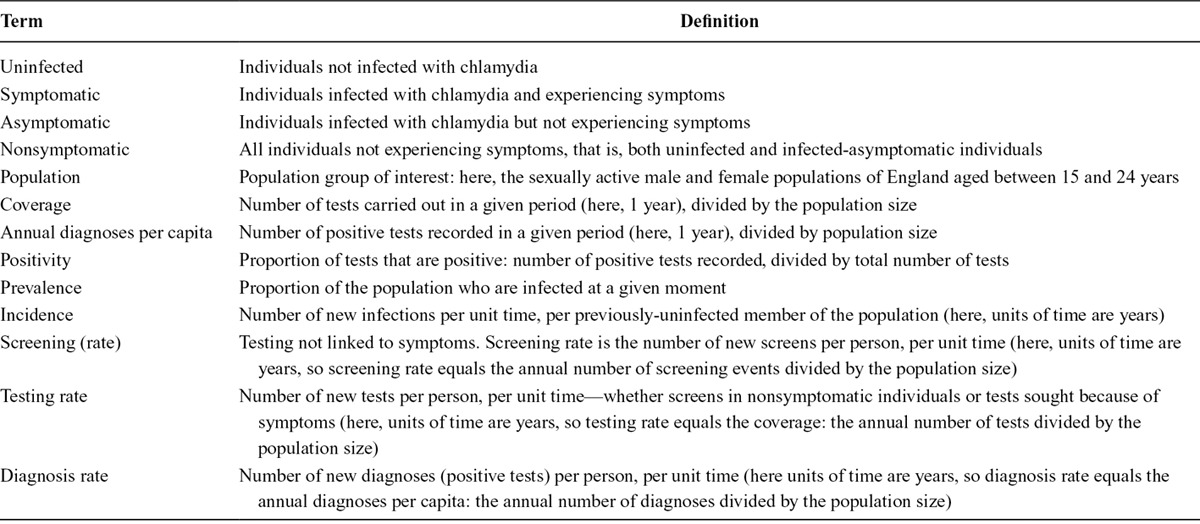
Definitions of Terminology Used in the Text

### A Model of the Surveillance Process

**FIGURE 1.**
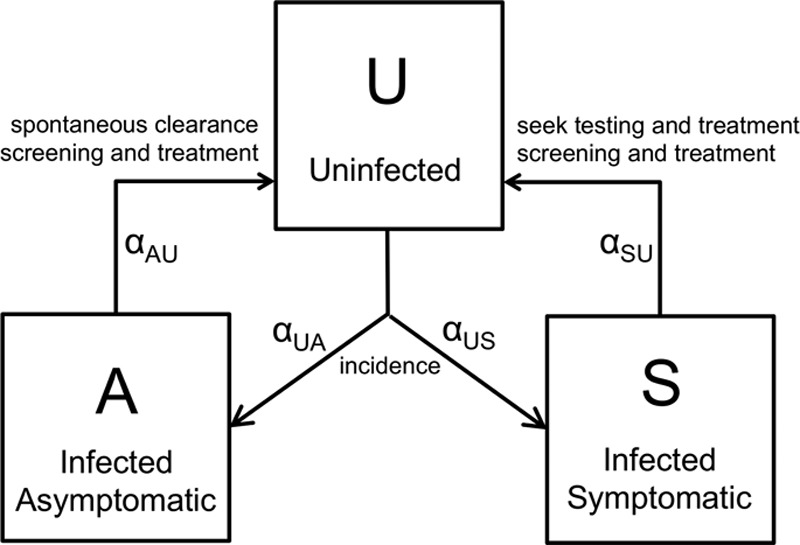
A model of chlamydia infection, clearance, testing, and treatment.


We use a three-compartment model of chlamydia infection and testing of symptomatic and nonsymptomatic individuals, illustrated in Figure 1. Uninfected individuals (*U*) become infected with a constant incidence, and move to either the asymptomatic (*A*) or symptomatic (*S*)-infected pools. Asymptomatic-infected individuals may leave *A* and return to *U* by spontaneous clearance of their infection or by detection and treatment under a national screening program. Symptomatic individuals may similarly be screened and treated, and will also actively seek treatment—typically at a rate much higher than the rates of spontaneous clearance or screening. (We assume that all nonsymptomatic people are tested with the same probability per unit time, regardless of individual risk behavior, as corresponding information is not available from surveillance data.) The system can be described using differential equations:

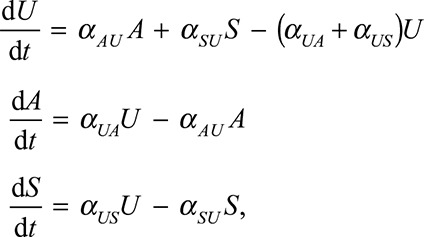
(1)

where


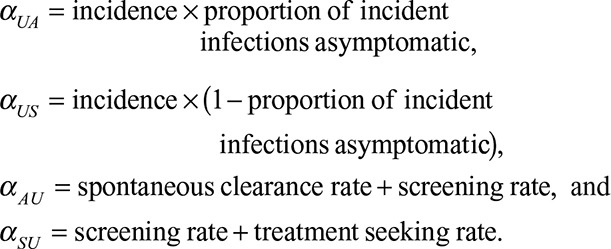


The testing rate is the number of tests conducted per person per year, and is equal to the per-person rate of screening, plus the rate at which new tests are sought in response to symptoms:


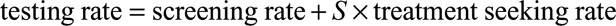


The diagnosis rate is the number of diagnoses made per person per year, and is equal to the screening rate multiplied by the proportion of individuals infected (*A* + *S*), plus the rate at which new diagnoses are made by testing in response to symptoms. With corrections for false-positive and false-negative test results,


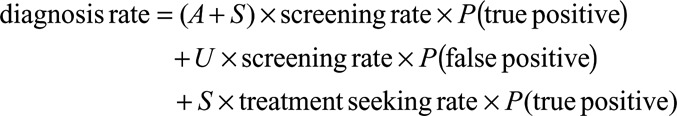


where *P*(true positive) is the probability of a positive test result, given that an individual is infected and *P*(false positive) is the probability of a positive test result, given that an individual is uninfected. As coverage and annual diagnoses per capita are defined in terms of tests and diagnoses recorded over 1 year and our rates are in units of years^−1^, the coverage is numerically equal to the testing rate and the annual diagnoses per capita equals the diagnosis rate.

In all our analyses, we assume that the system represented in Figure 1 is at steady state: that is, that the derivatives given in Equations (1) are all equal to zero. We normalize all results by specifying that the total population size, *U* + *A* + *S* = 1. Under these assumptions, we can solve Equations (1) for the proportion of the population in compartments *U*, *A*, and *S*.

### Estimating Prevalence from Surveillance Data

We used the model to investigate the incidence and screening rates underlying observed numbers of chlamydia tests and diagnoses. This was achieved in two stages. First, we sampled from prior distributions for all the model parameters apart from incidence, screening rate, and the proportion of incident infections which are asymptomatic. We also sampled for the national testing and diagnosis rates from gamma distributions informed by the numbers of tests and diagnoses observed, and for prevalence in 16- to 24-year-old men and women from beta distributions informed by estimates from the third National Study of Sexual Attitudes and Lifestyles (Natsal-3).^2^ We used this to approximate the 15- to -24-year-old prevalence, as no 15-year-olds were included in Natsal-3. Using these three values, we solved the model Equations (1) at steady state for each sample to obtain three unknowns: national incidence, per-capita screening rate, and the proportion of incident infections which do not lead to symptoms (eAppendix 2; http://links.lww.com/EDE/B187, Sections 1.1–1.2).

In the second stage, we used these samples for the proportion of infections which are asymptomatic to generate samples for prevalence in subpopulations defined by sex and age group (15–19 years vs. 20–24 years; eAppendix 2; http://links.lww.com/EDE/B187, Section 1.3), and by sex and local authority (eAppendix 3; http://links.lww.com/EDE/B187, Section 1.2). The procedure was similar: for each subpopulation, we sampled testing and diagnosis rates based on population size and numbers of tests and diagnoses, and solved the model Equations (1) at steady state to obtain the incidence and per-capita screening rates corresponding to the parameter values and the observed data. This provided distributions for the incidence and screening rates, which were used to derive distributions for prevalence. A total of 10,000 samples were drawn from each distribution.

### Investigating the Potential Benefit of Routinely Recording Symptoms

Two-dimensional data (numbers of tests and diagnoses) allow solution of Equations (1) for two unknowns: incidence and screening rate. In the method described above the third parameter, the proportion of incident infections which are asymptomatic, was obtained by calibration to national prevalence. With additional information—for example, the proportion of diagnosed infection that is in asymptomatic cases—this parameter could be estimated directly without the need for calibration.

Although data on symptoms in those tested and diagnosed is not currently recorded routinely in surveillance data for England, we investigated the potential for this information to improve prevalence estimates by proposing for each sampled parameter set that some number between zero and all of the diagnoses observed were in asymptomatic patients. These proposals were drawn from a discrete uniform distribution and provided hypothetical symptomatic and asymptomatic diagnosis rates, related to the model variables and parameters by the equations:


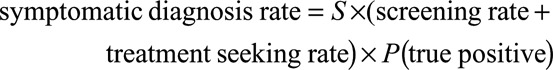


and


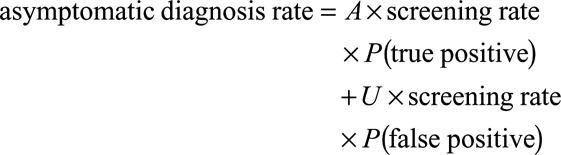


Using these relationships, Equations (1) could be solved at steady state to obtain incidence, screening, and the proportion of incident infections which are asymptomatic, and hence estimate prevalence given the proposed division between symptomatic and asymptomatic diagnoses.

### Data and Prior Distributions

We downloaded numbers of chlamydia tests and diagnoses in men and women ages 15–19, 20–24, and 15–24 years, and the corresponding populations of local authorities in England during 2012, from the NCSP website.^4^ Indices of multiple deprivation for 2010 were downloaded from the UK Official Statistics website.^5^ The index used here is the rank of the average deprivation score, with one indicating the most deprived district in England.

The prior distributions for test sensitivity and specificity were beta distributions parameterized directly from literature studies^6,7^ (Table 2). Distributions for the rates of natural recovery in men and women were sampled by Markov Chain Monte Carlo methods according a mixture-of-exponentials model,^8^ using the STAN software^9^ and informed by literature data.^10–22^ The prior for the rate of treatment seeking by symptomatic cases was also sampled using Markov Chain Monte Carlo, based on published data.^23^ These methods are a way to generate samples from a probability distribution for which direct sampling is difficult—for example, as in this case, because the exact form is not known.^24^ Full details of the sampling are provided in eAppendix 2 (http://links.lww.com/EDE/B187).

**TABLE 2.**
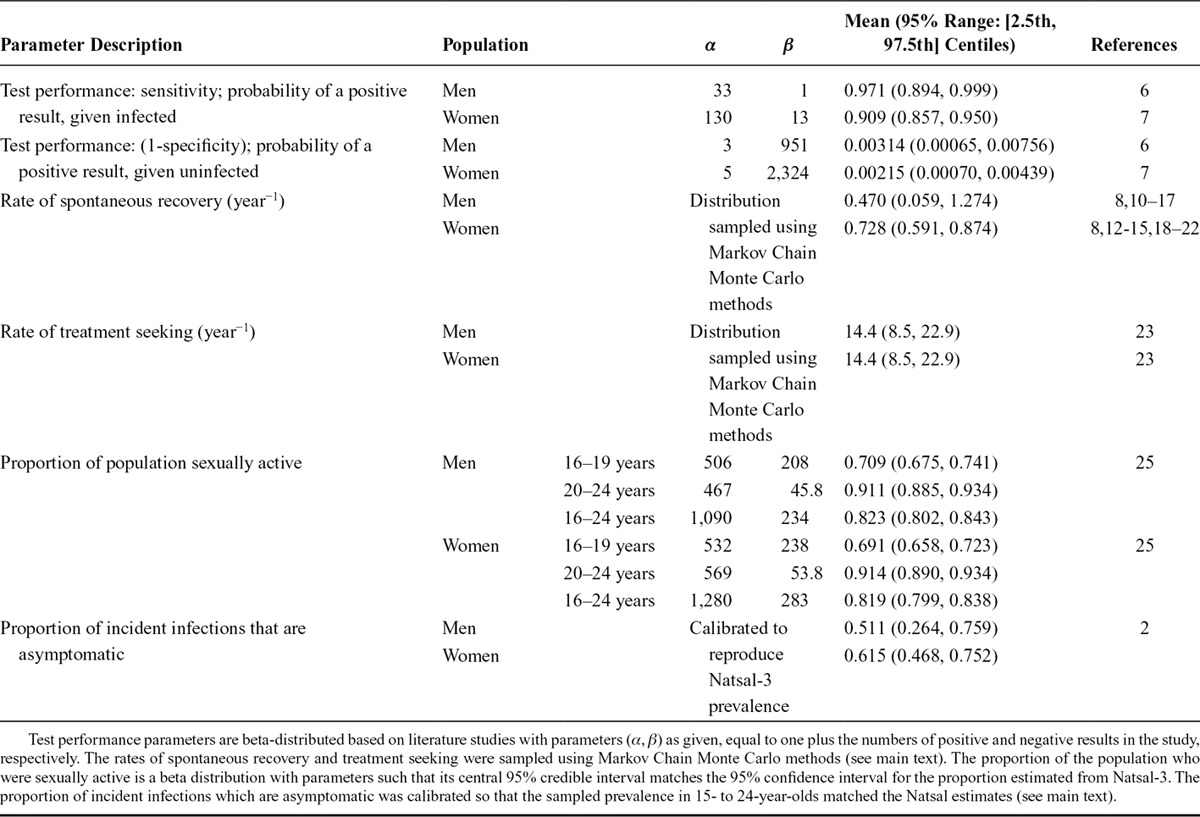
Distributions Used as Priors for Model Parameters

Data from Natsal-3^25^ were used to estimate a 95% confidence interval for the proportion of 16- to 24-year-old men and women who are sexually active, which was then used to parameterize the corresponding beta distribution. Reported prevalence estimates from Natsal-3^2^ were used for validation of our method. Natsal-3 participants were aged 16 or over and came from England, Scotland, and Wales, so information from Natsal on 16- to 24-year-olds was used as the best estimate of equivalent information for English 15- to 24-year-olds (the age groups presented in the NCSP data).

### Software

We carried out our analysis mainly in the Python language. The survey package in the R environment was used for analyzing Natsal-3 data, and the STAN software^9^ for Markov Chain Monte Carlo sampling of spontaneous clearance rates. The code used for analysis and for preparing Figures is available in the Supplemental Digital Content (http://links.lww.com/EDE/B187), and online at https://github.com/joanna-lewis/ct_surveillance.

### Ethical Review

No ethical review was required for this analysis of previously published data.

## RESULTS

### Model Behavior

**FIGURE 2.**
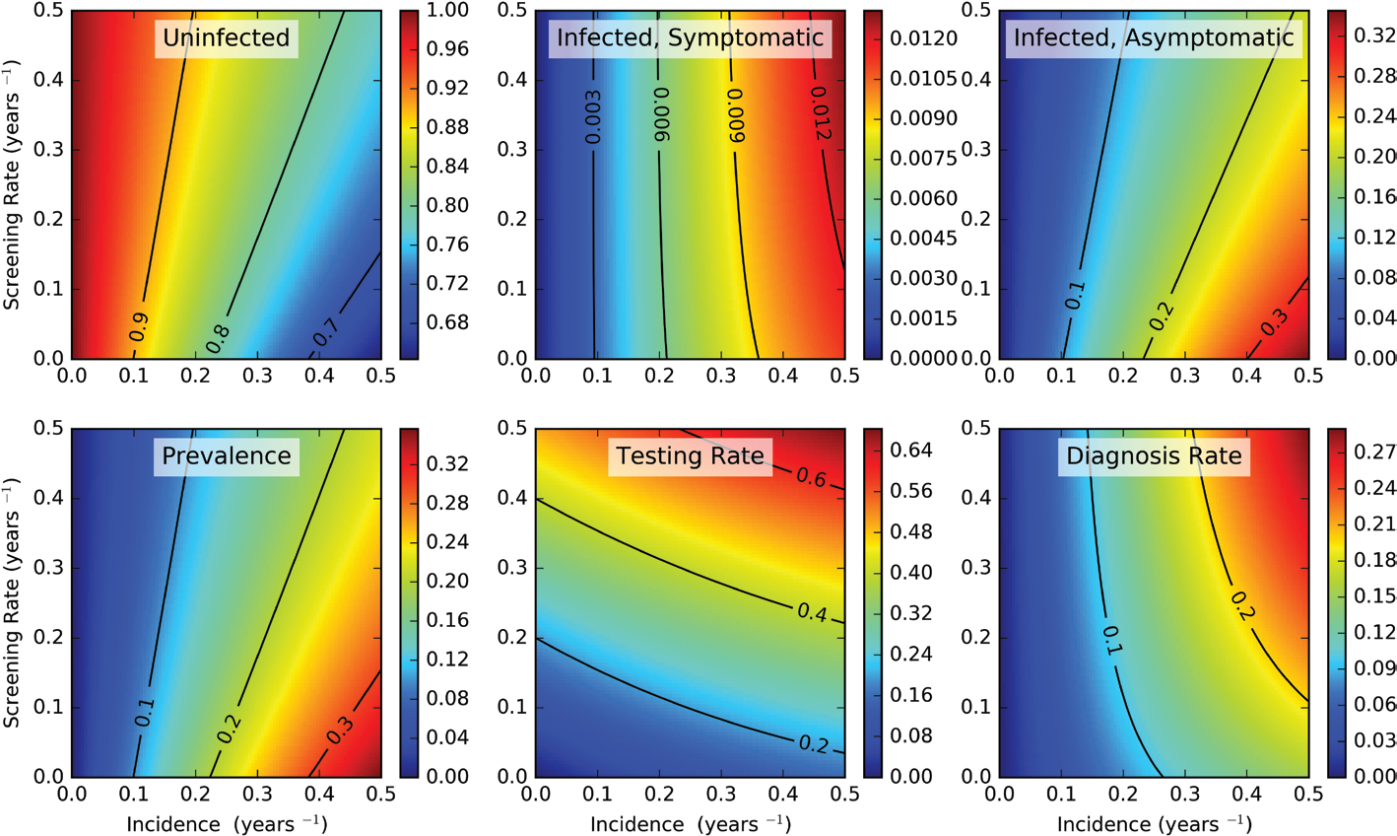
Upper row, The effects of incidence and screening rate on the proportion of individuals who are uninfected (*U*), infected-symptomatic (*S*), and infected-asymptomatic (*A*) in the model at steady state. Because they represent proportions, values of *U*, *S*, and *A* always fall in the interval (0,1). Lower row, Prevalence, testing, and diagnosis rates corresponding to each combination of incidence and screening rate.

To investigate the behavior of the model, we fixed parameter values at the mean of the distributions for men in Table 2 and varied the incidence and screening rate. Figure 2 shows how incidence and screening rate affect the equilibrium proportions of individuals in the uninfected, infected-symptomatic, and infected-asymptomatic compartments. As one would expect, the proportion of uninfected individuals is highest when incidence is low and nonsymptomatic screening rates are high. Conversely, the proportion of asymptomatic-infected individuals is high if incidence is high or screening rates are low. The prevalence of symptomatic infection is generally low because people with symptoms seek treatment quickly, but increases with higher incidence. It is fairly insensitive to screening rate because screening makes only a small contribution to the testing and treatment of these individuals, compared with the role of treatment seeking due to symptoms. Also shown in Figure 2 are the prevalence, testing rate, and diagnosis rate across the same incidence and screening ranges. Prevalence is simply the complement of the proportion of the population uninfected, (1−*U*), and therefore increases with higher incidence and lower screening rates. Testing rate increases with higher incidence, due to increased amounts of symptomatic care seeking, and also with higher screening. Diagnosis rate increases with higher incidence, which raises prevalence, and at higher screening where the increase in case detection more than compensates for the slightly lowered prevalence.

### Validation: Estimated National Chlamydia Prevalence by Age Group Agrees with Estimates from a Population-based Study

**FIGURE 3.**
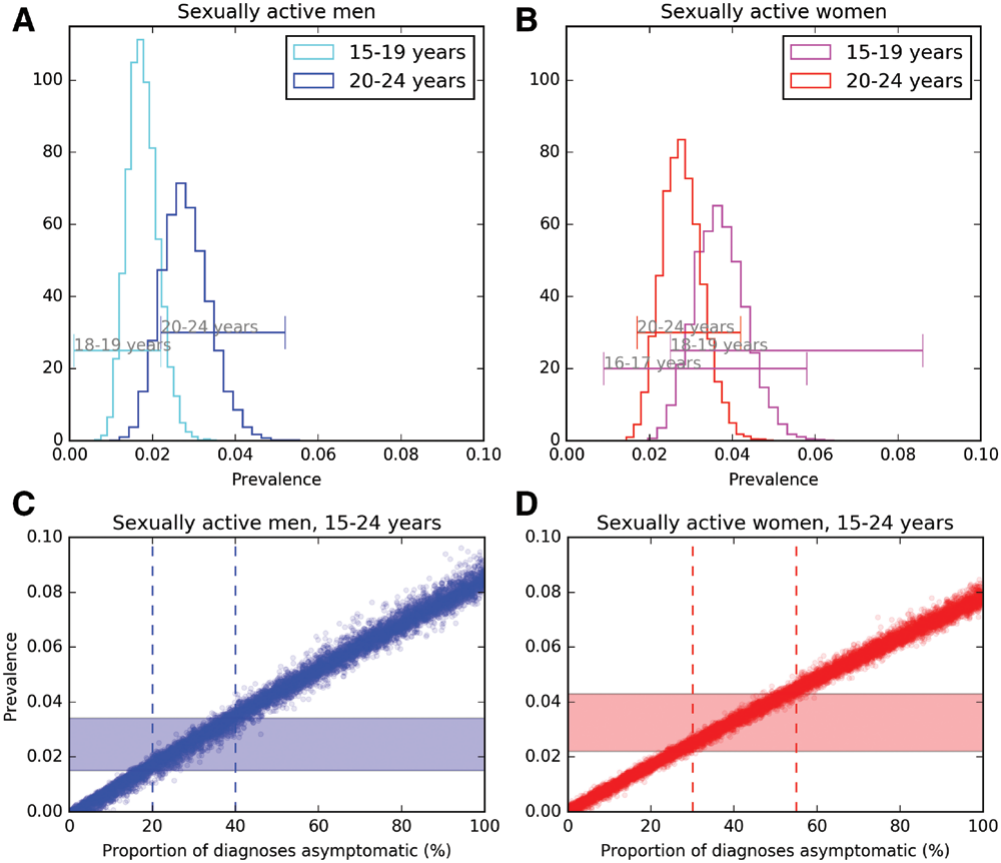
A and B, Sampled distributions for national chlamydia prevalence in England in sexually active 15- to 19- and 20- to 24-year-old men and women, estimated from 2012 testing and diagnosis data using the model. Error bars show the central 95% confidence interval for prevalence in men and women ages 16–17, 18–19, and 20–24 years in Natsal-3. C and D, Estimated prevalence, given different proportions of symptomatic and asymptomatic diagnoses making up the total reported. Shaded areas show the central 95% confidence interval for the prevalence in men and women ages 16–24 years in Natsal-3.

Figure 3A, B shows the sampled distributions for chlamydia prevalence in 15- to 19- and 20- to 24-year-old men and women in England during 2012, which overlap well with the corresponding Natsal-3 estimates.^2^ The calibration of the proportion of infections developing symptoms means that the weighted average of the prevalence estimates will inevitably match the Natsal-3 prevalence. However, the age-specific estimates are free to vary subject to this constraint, so the agreement between estimates and age-specific Natsal-3 observations provides confidence in our method.

### Recording the Numbers of Symptomatic and Asymptomatic Diagnoses Could Improve Estimates

Figure 3C, D shows how additional information about the numbers of symptomatic and asymptomatic diagnoses would affect estimates of prevalence. The larger the proportion of diagnosed cases that are asymptomatic, the higher the estimated prevalence. The number of diagnoses provides a lower bound on the number of infections in the population. If only a small proportion of diagnoses are in asymptomatic patients then we may deduce that a small proportion of incident infections are asymptomatic, and hence that there are few undiagnosed, asymptomatic cases in the population so the total number of infections is near to this lower bound. The observed chlamydia prevalence in Natsal-3 would be consistent with around 20%–40% of diagnoses in men and 30%–55% in women being asymptomatic (Figure 3). By reducing the uncertainty associated with the proportion of infections that are asymptomatic, the additional information improves the precision of prevalence estimates—whether at a national (as illustrated here) or a local level.

### Local Differences in Chlamydia Prevalence

Coverage, annual diagnoses per capita, and positivity by local authority vary substantially across England (coverage 5%–48% in men, 15%–78% in women; annual diagnoses per capita 1%–4% in men, 1%–8% in women; positivity 5%–16% in men, 5%–11% in women). We used the model to investigate whether the variation in diagnoses and positivity corresponds to important differences in prevalence by using local (testing, diagnosis) pairs to estimate local prevalence.

**FIGURE 4.**
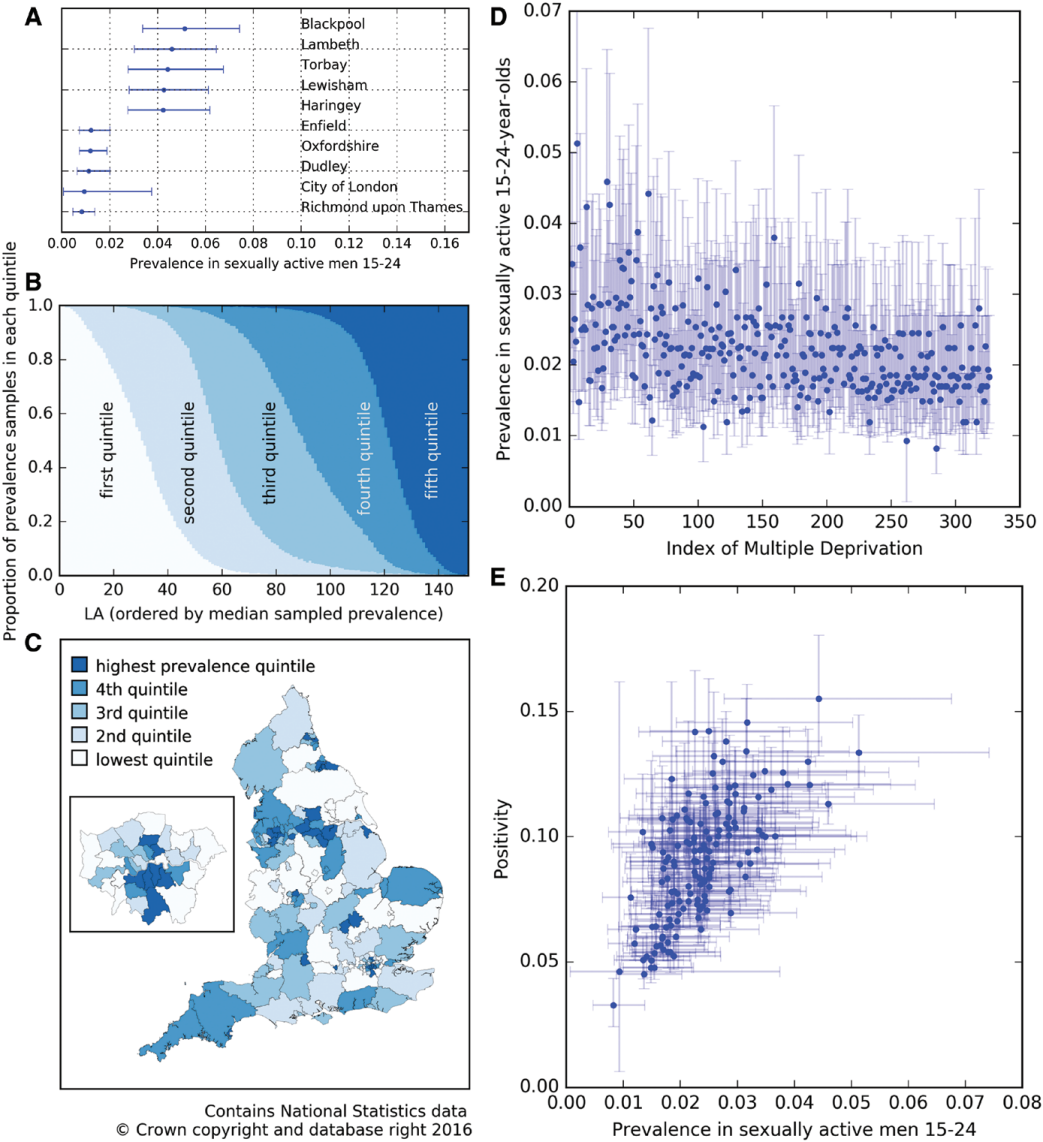
Local variation in chlamydia prevalence in men. A, Estimated prevalence in the local authorities with the five highest and lowest estimated chlamydia prevalence. B, Proportion of samples placing each local authority in quintiles 1–5 for chlamydia prevalence nationwide. C, Local authorities in England, colored by quintile for chlamydia prevalence (determined by the median sample). Inset, The London boroughs. D, Estimated prevalence by local authority, shown against index of multiple deprivation by local authority district, with most deprived districts (lowest index) on the left. Where a local authority consists of more than one district, multiple points with the same prevalence are shown. E, Correlation between estimated prevalence and positivity. In all plots, the markers and error bars indicate the 2.5th, 50th, and 97.5th centiles of the sampled distributions. The Scilly Isles were excluded because of small numbers. Equivalent plots for women may be found in eAppendix 3 (http://links.lww.com/EDE/B187).

Estimated prevalence (median sample) across the local authorities ranges from 0.8% to 5.1% in men and 1.8% to 5.6% in women ages 15–24 years. Figure 4A summarizes the sampled distributions for prevalence in men in the five highest and lowest prevalence of 151 local authorities in England. In general, the 95% credible intervals for the highest and lowest local authorities do not overlap at all, or only slightly, but there are over 100 local authorities with intermediate prevalence in which the distributions do overlap. (A plot showing all local authorities can be obtained using the code supplied.) However, although there is uncertainty in absolute prevalence values due to uncertainty in model parameters that are constant across local authorities (Figure 4A), the rank order of local authorities by prevalence is much more robust (Figure 4B): most local authorities are placed by the model in the same quintile for prevalence by most of the sampled parameter sets.

There is geographic heterogeneity in estimated chlamydia prevalence (Figure 4C), with higher prevalence tending to be found in more deprived areas (Figure 4D). This is consistent with the finding from Natsal-3 that individuals in more deprived areas were more likely to have chlamydia.^2^ We note, however, that there is substantial variation in prevalence between local authorities with similar levels of deprivation.

### Positivity Is Not a Proxy for Prevalence

Figure 4E shows positivity by local authority, plotted against estimated prevalence. Although there is a positive correlation between estimated population prevalence and positivity of those tested, positivity is consistently higher than estimated prevalence because the sample of individuals tested is “enriched” for infection by those seeking treatment because of symptoms. There are also a large number of possible comparisons between local authorities where the authority with the lower positivity has the higher estimated prevalence. This result argues against the use of positivity as a proxy for prevalence if an alternative is available.

### Prevalence in Each Sex Strongly Predicts Incidence in the Other

**FIGURE 5.**
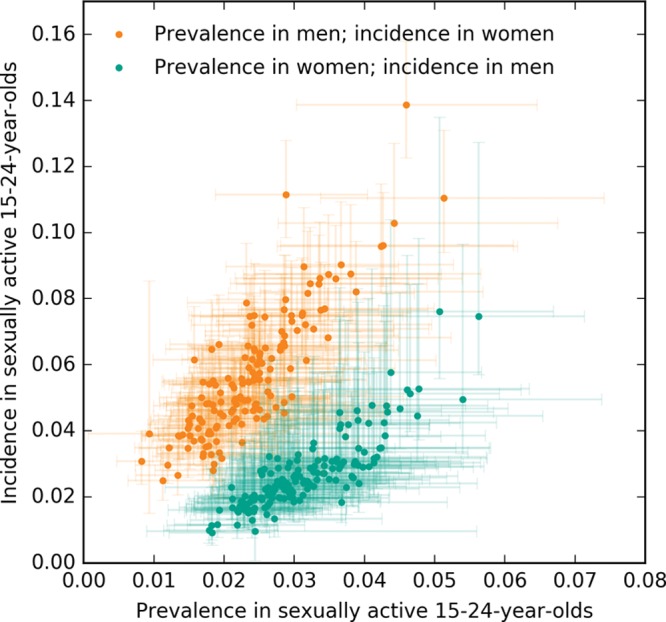
Relationship between incidence in each sex and prevalence in the other. Orange indicates the relationship between prevalence in men and incidence in women, and green shows the relationship between prevalence in women and incidence in men. The error bars give the 2.5th, 50th, and 97.5th centiles of the sampled distributions.

Finally, in Figure 5, we plot estimated incidence in each sex against estimated prevalence in the other. The two are proportional, which is expected if sexual behavior relating to transmission risk is broadly similar across local authorities. The relative gradients of the incidence-prevalence lines suggest that male-to-female transmission rates are higher than female-to-male, consistent with the literature.^26^ Deviation away from the line of constant proportionality might suggest local variation in behavior meriting further investigation, as discussed below.

## DISCUSSION

Population-based surveys of chlamydia prevalence are expensive, so they are infrequent and typically designed to be representative at national but not local level. It is therefore not feasible to examine short-term or local-level variation in prevalence through direct measurement, and so surveillance data are used. Importantly, we have found that local positivity among those tested may not provide a reliable proxy for local population prevalence. We have developed an alternative, synthesizing information from numbers of chlamydia tests and diagnoses and studies of chlamydia’s natural history to estimate the prevalence of infection from single-time-point data, and at a finer spatial scale than has previously been possible. The method is validated by reproducing prevalence by age group (15–19 and 20–24 years) from the Natsal-3 study. The approach is also applicable to other countries that collect data through widespread chlamydia testing programs.

Using our method, we found a range of prevalence across local authorities, but the differences between most local authorities are smaller than the range of uncertainty in individual estimates. Nevertheless, the order of local authorities by prevalence was found to be robust. We estimated higher prevalence in more deprived areas, consistent with Natsal-3’s finding that for individuals, being infected with chlamydia was associated with local deprivation.^2^

There is a linear relationship between prevalence in each sex and incidence in the other (Figure 5). This is the pattern expected if areas have the same sexual transmission risk behavior (partner change rate, mixing patterns, condom use, etc). Deviations away from the line of constant proportionality (i.e., differences in the incidence-prevalence relationship) are small compared with the range of local estimates on each axis, and might suggest local behavioral differences including age-mixing, distributions of partner numbers, or levels of condom use. They could also reflect same-sex transmission and extragenital (pharyngeal or rectal) infection, which would be included in the NCSP figures. Variation along the line of proportionality indicates a difference in prevalence with some other cause. A potential candidate would be differences in screening coverage, but incidence and prevalence are in fact positively correlated with levels of nonsymptomatic screening (eAppendix 3; http://links.lww.com/EDE/B187). A given difference in prevalence has a greater estimated effect on incidence in the male-to-female than the female-to-male direction, perhaps because the per-sex-act transmission risk is higher from male to female, an idea consistent with the literature.^26^ Alternatively, the difference could reflect different behavioral characteristics with, for example, infected men having in general more sexual partners than infected women.

The major limitation of the current analysis, and an important source of uncertainty in the prevalence estimates obtained, is uncertainty in the proportion of incident infections that are asymptomatic. We obtained estimates by calibration to Natsal-3. Research studies to measure this parameter directly are practically and ethically problematic, so inference from fitting models to observational surveillance data is the most likely way to reduce uncertainty and improve estimates for simulation studies. This requires collection of more detailed surveillance data, recording numbers of symptomatic and nonsymptomatic patients tested for and diagnosed with chlamydia. This information is not currently part of England’s chlamydia screening data specification,^27^ although there has been some reporting previously.^28,29^ As illustrated in Figure 3C, D, information on the number of diagnoses which were asymptomatic could considerably improve both the precision and the accuracy of prevalence estimates: precision, because it narrows the credible interval, and accuracy because estimates are clearly sensitive to information about the proportion of infections asymptomatic in each sex. Comparison of Figure 3C, D to the prevalence estimated in Natsal-3 suggested that around 20%–40% of diagnoses in men and 30%–55% in women were asymptomatic.

The model is also sensitive to other parameters informed by the literature, and more detailed surveillance could improve our local prevalence estimates. The rates of treatment seeking in both sexes and spontaneous clearance of infection in men are poorly defined (with a wide distribution), although posterior predictive checks indicate well-specified models (eAppendix 2; http://links.lww.com/EDE/B187). Recording in surveillance data, the duration of symptoms before testing would improve estimates of the rate of treatment seeking. Recently treated cases may be at an increased risk of reinfection from an infected, untreated partner; routine reporting of partner testing and treatment would allow the model to be refined. Other high-risk groups may also be more likely to be tested, but analysis of the model’s properties under such conditions, informed by the magnitude of national-level correlations between risk behavior, testing, and diagnosis^30^ suggest that our estimates are only marginally sensitive even to strong risk factors for infection (eAppendix 1; http://links.lww.com/EDE/B187, Section 1.2); good local data could be used to check these issues for local-level estimates. Although the NCSP data are close to complete, it is possible that the coverage or annual diagnoses per capita might have been slightly underestimated, and Figure 2 shows that we would then infer a different incidence and screening rate, and hence an incorrect prevalence. Finally, we assume a steady state and a closed population when in fact individuals are constantly entering and leaving the population. In this case, the numbers of tests and diagnoses reported in the years following 2012 suggest the approximation is appropriate (eAppendix 1; http://links.lww.com/EDE/B187, Section 1.1), but we recommend that whenever the model is applied the steady-state assumption should be checked as described in Supplemental Digital Content (http://links.lww.com/EDE/B187).

The approach we describe provides a tool to enable important questions about chlamydia epidemiology to be addressed, facilitating greater use of data to inform action and reduce inequality. There is a need to understand the variation in chlamydia prevalence we have identified, particularly the substantial variation between local authorities with similar levels of deprivation. How much of the variation in prevalence is due to variation in characteristics of the local populations and how much is due to some local authorities having more effective programs? More detailed information from surveillance systems about individuals tested for and diagnosed with chlamydia—for example, age in years (rather than categories), ethnicity, and recent sexual behavior including number and sex of partners—would allow separate prevalence estimates in particular subpopulations, as a way of identifying sociodemographic groups at risk and examining the reasons for local variations in prevalence and the correlation with deprivation. To derive the relevant denominator data for behavioral groups—to determine what proportion of each local population belongs to each risk-behavior category—so that not only the number of tests but also the local annual per-person testing rate for a group can be estimated, will require statistical methodological work to be undertaken to synthesize local-level demographic data (available from the UK Office for National Statistics) with data from Natsal-3 on nationally-representative demographic correlates of risk behavior. Finally, there is likely to be geographic heterogeneity in prevalence within local authorities,^31^ and the method could also be applied at a finer spatial scale subject to suitable data being available.

We have described, illustrated, and validated a method for estimating chlamydia incidence and prevalence from numbers of tests and diagnoses: data collected routinely as part of the NCSP and testing programs in some other countries. Our model provides a useful tool for examining risk factors and patterns of infection and assessing and addressing health inequalities across localities and sociodemographic groups.

## ACKNOWLEDGMENTS

We thank the three reviewers for their helpful comments.

## Supplementary Material
