# Supplementary material for: Estimating Local Chlamydia Incidence and Prevalence Using Surveillance Data
Source: Epidemiology. 2017 Jun 1;28(4):492–502. doi: 10.1097/EDE.0000000000000655 (PMC5457828; doi:10.1097/EDE.0000000000000655)
Supplement: Supplementary file 1 [file ede-28-492-s001.pdf]

Estimating local chlamydia incidence and prevalence using surveillance data: eAppendix 1

Joanna Lewis and Peter White

August 23, 2016

Contents

1 A model for chlamydia surveillance data **1**
1.1 Steady-state assumption 6
1.2 Different testing rates in different populations 11

1 A model for chlamydia surveillance data

We propose a three-compartment model of chlamydia infection, testing and screening in a closed population, as illustrated below. Uninfected individuals (U) become infected with a constant incidence, and move to either the asymptomatic-infected (A) or symptomatic-infected (S) pool. Asymptomatic-infected individuals may leave A and return to U by spontaneous clearance of their infection or by detection and treatment under a screening programme. Symptomatic individuals may similarly be screened, but will also seek treatment at a rate which is typically much higher than the rates of spontaneous clearance or screening.

```
In [1]: from IPython.display import Image
        Image(filename="figures/3_comp.png", width=500)
```

Out[1]:Out[1]:

This dynamic model has a steady-state solution which depends on the transition rates α_{UA}, α_{AU}, α_{US} and α_{SU}:

```
In [2]: import sympy as sym
        from sympy import *
        A, U, S = symbols("A U S")
        alpha_UA, alpha_AU, alpha_US, alpha_SU  = symbols("alpha_UA alpha_AU alpha_US alpha_SU")

        model_dyn = [
            alpha_UA*U - alpha_AU*A,
            alpha_AU*A + alpha_SU*S - (alpha_UA + alpha_US)*U,
            alpha_US*U - alpha_SU*S,
            A + U + S - 1 # this equation sets the total population size to 1
            ]

        # steady-state solution
        sol_dyn = solve(model_dyn, A, U, S)

        # functions for calculating the proportion of the population in each compartment at
        # steady state, given transition rates between compartments
        dyn_fun = lambdify((alpha_UA, alpha_AU, alpha_US, alpha_SU), sol_dyn[A] + sol_dyn[S])
        U_fun = lambdify((alpha_UA, alpha_AU, alpha_US, alpha_SU), sol_dyn[U])
        A_fun = lambdify((alpha_UA, alpha_AU, alpha_US, alpha_SU), sol_dyn[A])
        S_fun = lambdify((alpha_UA, alpha_AU, alpha_US, alpha_SU), sol_dyn[S])

        sol_dyn
```

```
Out[2]: {S: alpha_AU*alpha_US/(alpha_AU*alpha_US + alpha_SU*(alpha_AU + alpha_UA)),
         U: alpha_AU*alpha_SU/(alpha_AU*alpha_US + alpha_SU*(alpha_AU + alpha_UA)),
         A: alpha_SU*alpha_UA/(alpha_AU*alpha_US + alpha_SU*(alpha_AU + alpha_UA))}
```

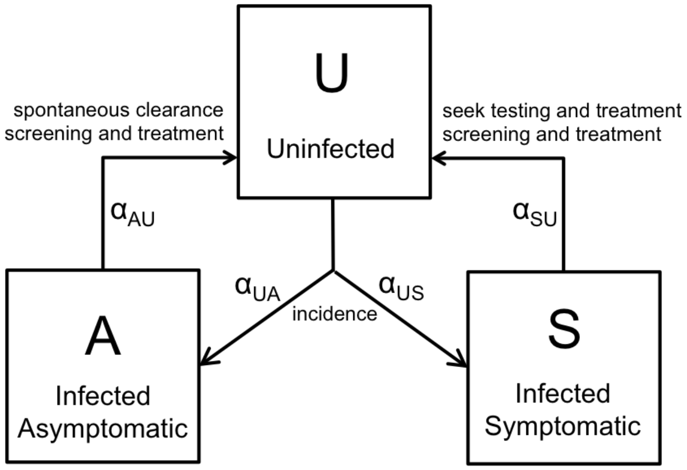

Figure 1: A model of chlamydia infection, clearance, testing and treatment.

The transition rates are functions of parameters describing behaviour and the natural history of infection:

$$\begin{aligned}
\alpha_{UA} &= \text{incidence} \times (1 - p_{symptomatic}) \\
\alpha_{AU} &= \text{rate of spontaneous clearance} + \text{rate of screening} \times p_{truepositive} \\
\alpha_{US} &= \text{incidence} \times p_{symptomatic} \\
\alpha_{SU} &= (\text{rate of screening} + \text{rate of symptomatic testing}) \times p_{truepositive}
\end{aligned}$$

Assuming all tests conducted are included in the surveillance data, the number of tests reported per unit time will be:

$$\text{rate of testing} = \text{rate of screening} + S \times \text{rate of symptomatic testing}$$

And the number of diagnoses per unit time will be:

$$\begin{aligned}
\text{rate of new diagnoses} = (A + S) \times (\text{rate of screening} \times p_{truepositive}) \\
+ (U \times \text{rate of screening} \times p_{falsepositive}) \\
+ (S \times \text{rate of symptomatic testing} \times p_{truepositive})
\end{aligned}$$

Let's assume (based on mean sampled values for men; see Table 2 of main text) that:

- 51.0% of incident infections are asymptomatic.
- Infections (whether symptomatic or not) clear spontaneously or through background antibiotic use at a rate 0.47 per year.
- Symptomatic cases seek and obtain testing and treatment at a rate 14.4 per year.
- 97.1% of tests in infected individuals return a positive result.
- 0.314% of tests in uninfected individuals return a positive result.

In [3]:
```
p_asymp = 0.510
sc = 0.47
att_symp = 14.4
p_true_pos = 0.971
p_false_pos = 0.00314
```

It is then possible to calculate the steady-state proportion of the population in each compartment given the rate of screening and incidence, and from these proportions to calculate the total prevalence, and the rates of new tests and diagnoses.

In [4]:
```
%matplotlib inline
from numpy import *
import matplotlib.pyplot as plt

inc = linspace(0, 0.5, 101) # incidence
scr = linspace(0, 0.5, 101) # screening
inc,scr = meshgrid(inc, scr)

# proportion of population in each compartment
ZU = U_fun(inc*p_asymp, sc + scr*p_true_pos, inc*(1-p_asymp), scr*p_true_pos + att_symp*p_true_pos)
ZA = A_fun(inc*p_asymp, sc + scr*p_true_pos, inc*(1-p_asymp), scr*p_true_pos + att_symp*p_true_pos)
ZS = S_fun(inc*p_asymp, sc + scr*p_true_pos, inc*(1-p_asymp), scr*p_true_pos + att_symp*p_true_pos)

Zprev = 1 - ZU
Ztest = scr + ZS*att_symp
Zdiag = (ZA+ZS)*scr*p_true_pos + ZU*scr*p_false_pos + ZS*att_symp*p_true_pos
```

In [5]:
```
fig = plt.figure(figsize = (12, 7))

ax1 = fig.add_subplot(231)
p = ax1.pcolor(inc,scr, ZU)
c = ax1.contour(inc,scr, ZU, [0.6,0.7,0.8,0.9], colors=['k','k','k','k'])
plt.clabel(c, manual = [(0.1,0.05), (0.2,0.05), (0.4,0.05)], fmt='%1.1f')
cb = fig.colorbar(p, ax=ax1)
#ax1.set_xlabel('Incidence')
ax1.set_ylabel('Screening Rate (years $^{-1}$)')
t = ax1.text(0.25, 0.45, 'Uninfected', ha='center', size='large')
t.set_bbox(dict(facecolor='white', alpha=0.7, edgecolor='None'))
ax1.set_ylim(0, 0.5)
ax1.set_xlim(0, 0.5)

ax2 = fig.add_subplot(232)
p = ax2.pcolor(inc,scr, ZS)
c = ax2.contour(inc,scr, ZS, (0.003,0.006,0.009,0.012), colors='k', manual=True)
plt.clabel(c, manual = [(0.1,0.35), (0.2,0.35), (0.35,0.35), (0.45,0.35)])
cb = fig.colorbar(p, ax=ax2)
t = ax2.text(0.25, 0.45, 'Infected, Symptomatic', ha='center', size='large')
t.set_bbox(dict(facecolor='white', alpha=0.7, edgecolor='None'))
ax2.set_ylim(0, 0.5)
ax2.set_xlim(0, 0.5)

ax3 = fig.add_subplot(233)
p = ax3.pcolor(inc,scr, ZA)
c = ax3.contour(inc,scr, ZA, (0.1,0.2,0.3), colors='k')
plt.clabel(c, manual = [(0.1,0.1), (0.2,0.1), (0.4,0.1)], fmt='%1.1f')
cb = fig.colorbar(p, ax=ax3)
t = ax3.text(0.25, 0.45, 'Infected, Asymptomatic', ha='center', size='large')
t.set_bbox(dict(facecolor='white', alpha=0.7, edgecolor='None'))
ax3.set_ylim(0, 0.5)
ax3.set_xlim(0, 0.5)

ax4 = fig.add_subplot(234)
p = ax4.pcolor(inc,scr, Zprev)
c = ax4.contour(inc,scr, Zprev, (0.1,0.2,0.3), colors='k')
plt.clabel(c, manual = [(0.1,0.1), (0.2,0.1), (0.4,0.1)], fmt='%1.1f')
cb = fig.colorbar(p, ax=ax4)
ax4.set_xlabel('Incidence  (years $^{-1}$)')
ax4.set_ylabel('Screening Rate (years $^{-1}$)')
t = ax4.text(0.25, 0.45, 'Prevalence', ha='center', size='large')
t.set_bbox(dict(facecolor='white', alpha=0.7, edgecolor='None'))
ax4.set_ylim(0, 0.5)
ax4.set_xlim(0, 0.5)

ax5 = fig.add_subplot(235)
p = ax5.pcolor(inc,scr, Ztest)
c = ax5.contour(inc,scr, Ztest, (0.2,0.4,0.6), colors='k')
plt.clabel(c, manual = [(0.45,0.05), (0.45,0.25), (0.45,0.45)], fmt='%1.1f')
cb = fig.colorbar(p, ax=ax5)
ax5.set_xlabel('Incidence  (years $^{-1}$)')
```

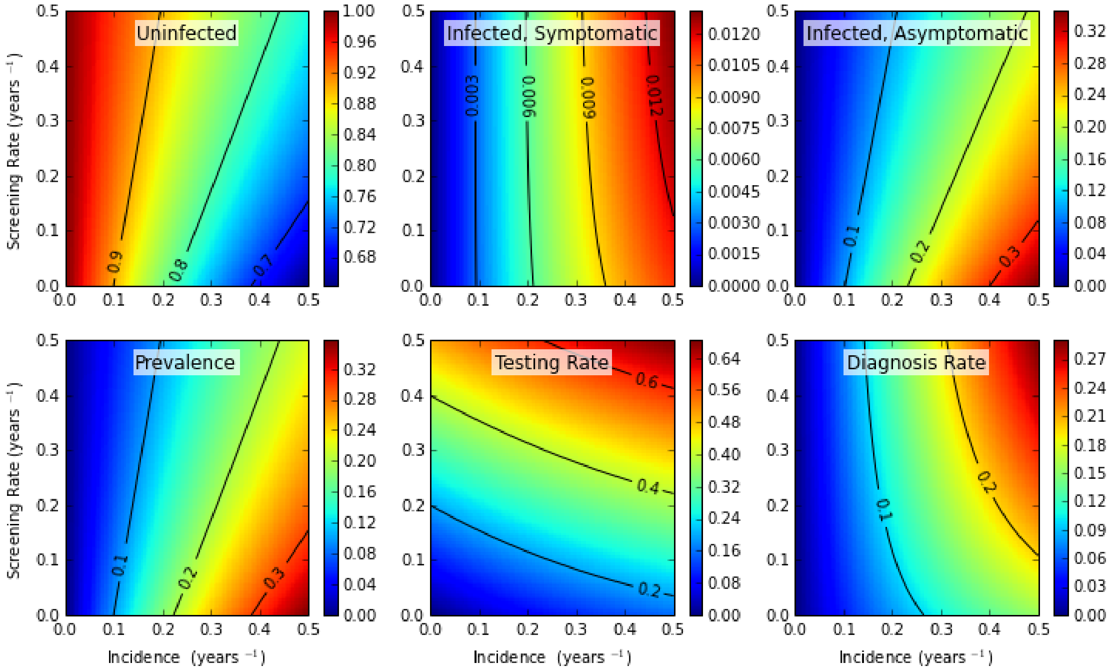

Figure 2: Upper row: the effects of incidence and screening rate on the proportion of individuals who are uninfected, infected-symptomatic and infected-asymptomatic in the model at steady state. Lower row: prevalence, testing and diagnosis rates corresponding to each combination of incidence and screening rate.

```
t = ax5.text(0.25, 0.45, ’Testing Rate’, ha=’center’, size=’large’)
t.set_bbox(dict(facecolor=’white’, alpha=0.7, edgecolor=’None’))
ax5.set_ylim(0, 0.5)
ax5.set_xlim(0, 0.5)

ax6 = fig.add_subplot(236)
p = ax6.pcolor(inc,scr, Zdiag)
c = ax6.contour(inc,scr, Zdiag, (0.1,0.2), colors=’k’)
plt.clabel(c, manual = [(0.2,0.2), (0.4,0.25)], fmt=’%1.1f’)
cb = fig.colorbar(p, ax=ax6)
ax6.set_xlabel(’Incidence (years $^{-1}$)’)
t = ax6.text(0.25, 0.45, ’Diagnosis Rate’, ha=’center’, size=’large’)
t.set_bbox(dict(facecolor=’white’, alpha=0.7, edgecolor=’None’))
ax6.set_ylim(0, 0.5)
ax6.set_xlim(0, 0.5)

plt.show()
```

From the figures, it is clear that a particular pair of observed testing and diagnosis rates corresponds to a single point in the (incidence, screening rate) plane, which in turn corresponds to a particular prevalence. Note, however, that this mapping depends on the parameter values which have been assumed.

We also produce the same plot, focussing on the lower part of the incidence range:

```
In [6]: inc = linspace(0, 0.1, 101) # incidence - different range
        scr = linspace(0, 0.5, 101) # screening
        inc,scr = meshgrid(inc, scr)

        # proportion of population in each compartment
        ZU = U_fun(inc*p_asymp, sc + scr*p_true_pos, inc*(1-p_asymp), scr*p_true_pos + att_symp*p_true_pos)
        ZA = A_fun(inc*p_asymp, sc + scr*p_true_pos, inc*(1-p_asymp), scr*p_true_pos + att_symp*p_true_pos)
        ZS = S_fun(inc*p_asymp, sc + scr*p_true_pos, inc*(1-p_asymp), scr*p_true_pos + att_symp*p_true_pos)

        Zprev = 1 - ZU
```

```
Ztest = scr + ZS*att_symp
Zdiag = (ZA+ZS)*scr*p_true_pos + ZU*scr*p_false_pos + ZS*att_symp*p_true_pos
```

In [7]:
```
fig = plt.figure(figsize = (12, 7))

ax1 = fig.add_subplot(231)
p = ax1.pcolor(inc,scr, ZU)
c = ax1.contour(inc,scr, ZU, [0.92,0.94,0.96,0.98], colors=['k','k','k','k'])
plt.clabel(c, manual = [(0.02,0.25), (0.05,0.25), (0.07,0.15), (0.09,0.05)], fmt='%1.2f')
cb = fig.colorbar(p, ax=ax1)
#ax1.set_xlabel('Incidence')
ax1.set_ylabel('Screening Rate (years $^{-1}$)')
t = ax1.text(0.05, 0.45, 'Uninfected', ha='center', size='large')
t.set_bbox(dict(facecolor='white', alpha=0.7, edgecolor='None'))
ax1.set_ylim(0, 0.5)
ax1.set_xlim(0, 0.1)

ax2 = fig.add_subplot(232)
p = ax2.pcolor(inc,scr, ZS)
c = ax2.contour(inc,scr, ZS, (0.001,0.002,0.003), colors='k', manual=True)
plt.clabel(c, manual = [(0.03,0.15), (0.06,0.15), (0.09,0.15)], fmt='%1.3f')
cb = fig.colorbar(p, ax=ax2)
t = ax2.text(0.05, 0.45, 'Infected, Symptomatic', ha='center', size='large')
t.set_bbox(dict(facecolor='white', alpha=0.7, edgecolor='None'))
ax2.set_ylim(0, 0.5)
ax2.set_xlim(0, 0.1)

ax3 = fig.add_subplot(233)
p = ax3.pcolor(inc,scr, ZA)
c = ax3.contour(inc,scr, ZA, (0.02,0.04,0.06,0.08), colors='k')
plt.clabel(c, manual = [(0.02,0.25), (0.05,0.25), (0.07,0.15), (0.09,0.05)], fmt='%1.2f')
cb = fig.colorbar(p, ax=ax3)
t = ax3.text(0.05, 0.45, 'Infected, Asymptomatic', ha='center', size='large')
t.set_bbox(dict(facecolor='white', alpha=0.7, edgecolor='None'))
ax3.set_ylim(0, 0.5)
ax3.set_xlim(0, 0.1)

ax4 = fig.add_subplot(234)
p = ax4.pcolor(inc,scr, Zprev)
c = ax4.contour(inc,scr, Zprev, (0.02,0.04,0.06,0.08), colors='k')
plt.clabel(c, manual = [(0.02,0.25), (0.05,0.25), (0.07,0.15), (0.09, 0.05)], fmt='%1.2f')
cb = fig.colorbar(p, ax=ax4)
ax4.set_xlabel('Incidence  (years $^{-1}$)')
ax4.set_ylabel('Screening Rate (years $^{-1}$)')
t = ax4.text(0.05, 0.45, 'Prevalence', ha='center', size='large')
t.set_bbox(dict(facecolor='white', alpha=0.7, edgecolor='None'))
ax4.set_ylim(0, 0.5)
ax4.set_xlim(0, 0.1)

ax5 = fig.add_subplot(235)
p = ax5.pcolor(inc,scr, Ztest)
c = ax5.contour(inc,scr, Ztest, (0.2,0.4), colors='k')
plt.clabel(c, manual = [(0.045,0.2), (0.045,0.5)], fmt='%1.1f')
cb = fig.colorbar(p, ax=ax5)
ax5.set_xlabel('Incidence  (years $^{-1}$)')
t = ax5.text(0.05, 0.45, 'Testing Rate', ha='center', size='large')
t.set_bbox(dict(facecolor='white', alpha=0.7, edgecolor='None'))
ax5.set_ylim(0, 0.5)
ax5.set_xlim(0, 0.1)

ax6 = fig.add_subplot(236)
p = ax6.pcolor(inc,scr, Zdiag)
c = ax6.contour(inc,scr, Zdiag, (0.02,0.04,0.06), colors='k')
plt.clabel(c, manual = [(0.04,0.2), (0.06,0.4), (0.09,0.35)], fmt='%1.2f')
cb = fig.colorbar(p, ax=ax6)
ax6.set_xlabel('Incidence (years $^{-1}$)')
t = ax6.text(0.05, 0.45, 'Diagnosis Rate', ha='center', size='large')
t.set_bbox(dict(facecolor='white', alpha=0.7, edgecolor='None'))
ax6.set_ylim(0, 0.5)
```

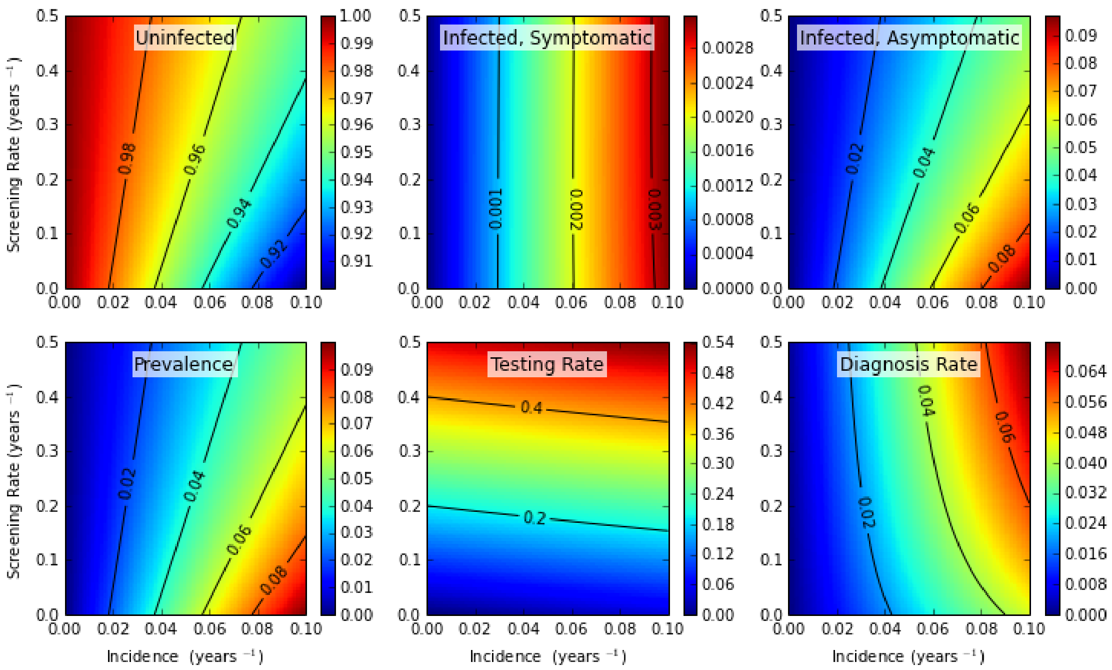

Figure 3: Upper row: the effects of incidence and screening rate on the proportion of individuals who are uninfected, infected-symptomatic and infected-asymptomatic in the model at steady state. Lower row: prevalence, testing and diagnosis rates corresponding to each combination of incidence and screening rate.

```
ax6.set_xlim(0, 0.1)

plt.show()
```

1.1 Steady-state assumption

In using the model to interpret testing and diagnosis data, we assume the system is at steady state. We investigate to what extent this assumption is valid by perturbing the system and observing the return to equilibrium.

First, we use the national coverage and diagnoses per capita in men in the years 2012 - 2015. The analysis proceeds as follows:

1. Begin by estimating the steady state in 2012, using 2012 data.
2. Assuming a (potentially different) steady state in 2013, estimate incidence and screening rate in 2013.
3. Starting at the 2012 steady state, simulate the evolution of the system for one year with 2013 incidence and screening figures. Compare the prevalence after one year with the steady-state prevalence estimated for 2013 from that year's surveillance data, to see how closely they agree.
4. Repeat steps 2 and 3 for 2014 and 2015, each time starting the system in the state it had reached at the end of the previous one-year period.

The results of the simulations are then plotted.

In [8]:
```
from scipy.optimize import fsolve

tsym, dsym, ssym, test_sym = symbols('tsym dsym ssym test_sym')

model_test_diag = [
    tsym - ( ssym + (1 - A - U)*test_sym ),
    dsym - ( A*ssym*p_true_pos + U*ssym*p_false_pos + (1 - A - U)*test_sym*p_true_pos )
```

```
    ]

sol_test_diag = solve(model_test_diag, tsym, dsym)
test_fun = lambdify((A, U, ssym, test_sym), sol_test_diag[tsym])
diag_fun = lambdify((A, U, ssym, test_sym), sol_test_diag[dsym])

def test_diag_fun(parms):
    # parms = (incidence, screening rate)
    inc = parms[0]
    scr = parms[1]

    A = A_fun(inc*p_asymp, sc + scr*p_true_pos, inc*(1 - p_asymp), scr*p_true_pos + att_symp*p_true_pos)
    U = U_fun(inc*p_asymp, sc + scr*p_true_pos, inc*(1 - p_asymp), scr*p_true_pos + att_symp*p_true_pos)
    return [test_fun(A, U, scr, att_symp), diag_fun(A, U, scr, att_symp)]


# set up a function to simulate system dynamics when perturbed from steady state
from scipy.integrate import odeint

def dydt(y, t, parms):
    return([
    parms[1]*y[1] + parms[3]*y[2] - (parms[0] + parms[2])*y[0],
    parms[0]*y[0] - parms[1]*y[1],
    parms[2]*y[0] - parms[3]*y[2]
    ])
```

In [9]:
```
# find steady state based on 2012 data

cov_2012 = 566908. / 3519015.
adpc_2012 = 48387. / 3519015.
[incsol, scrsol] = fsolve(
    lambda x: [test_diag_fun(x)[0] - cov_2012, test_diag_fun(x)[1] - adpc_2012],
    [0.09, 0.25]
    )

U_2012 = U_fun(
    incsol*p_asymp, sc + scrsol*p_true_pos, incsol*(1-p_asymp), scrsol*p_true_pos + att_symp*p_true_pos
    )
A_2012 = A_fun(
    incsol*p_asymp, sc + scrsol*p_true_pos, incsol*(1-p_asymp), scrsol*p_true_pos + att_symp*p_true_pos
    )
S_2012 = S_fun(
    incsol*p_asymp, sc + scrsol*p_true_pos, incsol*(1-p_asymp), scrsol*p_true_pos + att_symp*p_true_pos
    )


# find incidence and screening based on 2013 data
cov_2013 = 531428. / 3519015.
adpc_2013 = 48825. / 3519015.
[incsol, scrsol] = fsolve(
    lambda x: [test_diag_fun(x)[0] - cov_2013, test_diag_fun(x)[1] - adpc_2013],
    [0.09, 0.25]
    )

# solve, 2012-2013
inc = incsol
scr = scrsol
parms = \
    [incsol*p_asymp, sc + scrsol*p_true_pos, incsol*(1-p_asymp), scrsol*p_true_pos + att_symp*p_true_pos]

sol_12_13 = odeint(dydt,
       [U_2012,A_2012,S_2012],
       linspace(0,10,1000),
       args = (parms,)
      )
```

In [10]:
```
# incidence and screening based on 2014 data
cov_2014 = 493327. / 3500026.
adpc_2014 = 47437. / 3500026.
```

```
[incsol, scrsol] = fsolve(
    lambda x: [test_diag_fun(x)[0] - cov_2014, test_diag_fun(x)[1] - adpc_2014],
    [0.09, 0.25]
    )
inc = incsol
scr = scrsol
parms = \
    [incsol*p_asymp, sc + scrsol*p_true_pos, incsol*(1-p_asymp), scrsol*p_true_pos + att_symp*p_true_pos]

# solve, 2013-2014
sol_13_14 = odeint(dydt,
       sol_12_13[999,:],
       linspace(0,10,1000),
       args = (parms,)
      )
```

In [11]:
```
# incidence and screening based on 2015 data
cov_2015 = 446279. / 3496125.
adpc_2015 = 44609. / 3496125.
[incsol, scrsol] = fsolve(
    lambda x: [test_diag_fun(x)[0] - cov_2015, test_diag_fun(x)[1] - adpc_2015],
    [0.09, 0.25]
    )
inc = incsol
scr = scrsol
parms = \
    [incsol*p_asymp, sc + scrsol*p_true_pos, incsol*(1-p_asymp), scrsol*p_true_pos + att_symp*p_true_pos]

# solve, 2013-2014
sol_14_15 = odeint(dydt,
       sol_13_14[999,:],
       linspace(0,10,1000),
       args = (parms,)
      )
```

In [12]:
```
# plot solutions
plt.plot(linspace(2012,2013,1000), sol_12_13[:,1]+sol_12_13[:,2], label='2012-2013')
plt.plot(linspace(2013,2014,1000), sol_13_14[:,1]+sol_13_14[:,2], label='2013-2014')
plt.plot(linspace(2014,2015,1000), sol_14_15[:,1]+sol_14_15[:,2], label='2014-2015')
plt.ylim(0,0.025)
plt.ylabel('Prevalence')
plt.xticks([2012,2013,2014,2015], ['2012','2013','2014','2015'])
plt.legend(loc=4)
```

Out[12]: <matplotlib.legend.Legend at 0x10a9ae850>

The plot shows that prevalence was very close to the steady state, with differences being very small compared to the uncertainty in prevalence estimates illustrated in the Figures in the main text.

To investigate the validity of the steady state assumption at a local level, we identified the local authorities with the largest changes in prevalence between 2012 and 2013:

	Test rate	(year^{-1})	Diagnosis rate	(year^{-1})	Prevalence	
Year	2012	2013	2012	2013	2012	2013
North Lincolnshire	0.101	0.173	0.022	0.011	2.1%	3.9%
Haringey	0.267	0.191	0.035	0.018	4.2%	2.7%
Dudley	0.075	0.239	0.020	0.006	1.1%	2.4%

In [13]:
```
# North Lincolnshire
# find steady state based on 2012 data

cov_2012 = 0.100807801953
adpc_2012 = 0.0111652211547
[incsol, scrsol] = fsolve(
    lambda x: [test_diag_fun(x)[0] - cov_2012, test_diag_fun(x)[1] - adpc_2012],
    [0.09, 0.25]
    )
```

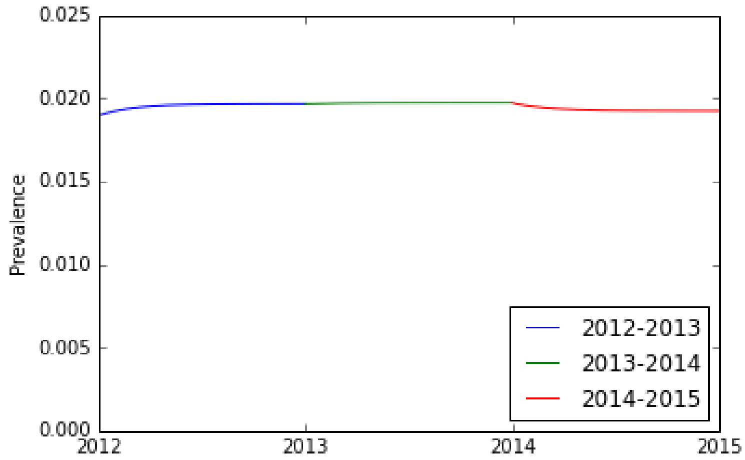

Figure 4: Simulated dynamics of national prevalence in 15-24-year-old men, 2012-2015.

```
U_2012 = U_fun(
    incsol*p_asymp, sc + scrsol*p_true_pos, incsol*(1-p_asymp), scrsol*p_true_pos + att_symp*p_true_pos
    )
A_2012 = A_fun(
    incsol*p_asymp, sc + scrsol*p_true_pos, incsol*(1-p_asymp), scrsol*p_true_pos + att_symp*p_true_pos
    )
S_2012 = S_fun(
    incsol*p_asymp, sc + scrsol*p_true_pos, incsol*(1-p_asymp), scrsol*p_true_pos + att_symp*p_true_pos
    )


# find incidence and screening based on 2013 data
cov_2013 = 0.173269822929
adpc_2013 = 0.0216211803756
[incsol, scrsol] = fsolve(
    lambda x: [test_diag_fun(x)[0] - cov_2013, test_diag_fun(x)[1] - adpc_2013],
    [0.09, 0.25]
    )

# solve, 2012-2013
inc = incsol
scr = scrsol
parms = \
    [incsol*p_asymp, sc + scrsol*p_true_pos, incsol*(1-p_asymp), scrsol*p_true_pos + att_symp*p_true_pos]

sol_n_lincs = odeint(dydt,
       [U_2012,A_2012,S_2012],
       linspace(0,10,1000),
       args = (parms,)
      )
```

In [14]:
```
# Haringey
# find steady state based on 2012 data

cov_2012 = 0.267007002375
adpc_2012 = 0.0346976493046
[incsol, scrsol] = fsolve(
    lambda x: [test_diag_fun(x)[0] - cov_2012, test_diag_fun(x)[1] - adpc_2012],
    [0.09, 0.25]
    )

U_2012 = U_fun(
    incsol*p_asymp, sc + scrsol*p_true_pos, incsol*(1-p_asymp), scrsol*p_true_pos + att_symp*p_true_pos
```

```
    )
A_2012 = A_fun(
    incsol*p_asymp, sc + scrsol*p_true_pos, incsol*(1-p_asymp), scrsol*p_true_pos + att_symp*p_true_pos
    )
S_2012 = S_fun(
    incsol*p_asymp, sc + scrsol*p_true_pos, incsol*(1-p_asymp), scrsol*p_true_pos + att_symp*p_true_pos
    )


# find incidence and screening based on 2013 data
cov_2013 = 0.190544970144
adpc_2013 = 0.0184872060681
[incsol, scrsol] = fsolve(
    lambda x: [test_diag_fun(x)[0] - cov_2013, test_diag_fun(x)[1] - adpc_2013],
    [0.09, 0.25]
    )

# solve, 2012-2013
inc = incsol
scr = scrsol
parms = \
    [incsol*p_asymp, sc + scrsol*p_true_pos, incsol*(1-p_asymp), scrsol*p_true_pos + att_symp*p_true_pos]

sol_haringey = odeint(dydt,
       [U_2012,A_2012,S_2012],
       linspace(0,10,1000),
       args = (parms,)
      )
```

In [15]:
```
# Dudley
# find steady state based on 2012 data

cov_2012 = 0.0750667240187
adpc_2012 = 0.0057129570304
[incsol, scrsol] = fsolve(
    lambda x: [test_diag_fun(x)[0] - cov_2012, test_diag_fun(x)[1] - adpc_2012],
    [0.09, 0.25]
    )

U_2012 = U_fun(
    incsol*p_asymp, sc + scrsol*p_true_pos, incsol*(1-p_asymp), scrsol*p_true_pos + att_symp*p_true_pos
    )
A_2012 = A_fun(
    incsol*p_asymp, sc + scrsol*p_true_pos, incsol*(1-p_asymp), scrsol*p_true_pos + att_symp*p_true_pos
    )
S_2012 = S_fun(
    incsol*p_asymp, sc + scrsol*p_true_pos, incsol*(1-p_asymp), scrsol*p_true_pos + att_symp*p_true_pos
    )


# find incidence and screening based on 2013 data
cov_2013 = 0.238873910562
adpc_2013 = 0.0199612670162
[incsol, scrsol] = fsolve(
    lambda x: [test_diag_fun(x)[0] - cov_2013, test_diag_fun(x)[1] - adpc_2013],
    [0.09, 0.25]
    )

# solve, 2012-2013
inc = incsol
scr = scrsol
parms = \
    [incsol*p_asymp, sc + scrsol*p_true_pos, incsol*(1-p_asymp), scrsol*p_true_pos + att_symp*p_true_pos]

sol_dudley = odeint(dydt,
       [U_2012,A_2012,S_2012],
       linspace(0,10,1000),
       args = (parms,)
      )
```

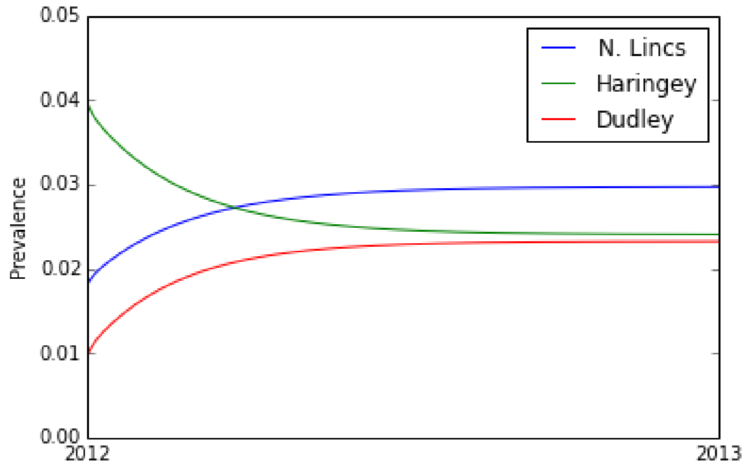

Figure 5: Simulated dynamics of local authority prevalence in 15-24-year-old men, 2012-2013.

```
In [16]: # plot solutions
         plt.plot(linspace(2012,2013,1000), sol_n_lincs[:,1]+sol_n_lincs[:,2], label='N. Lincs')
         plt.plot(linspace(2012,2013,1000), sol_haringey[:,1]+sol_haringey[:,2], label = 'Haringey')
         plt.plot(linspace(2012,2013,1000), sol_dudley[:,1]+sol_dudley[:,2], label = 'Dudley')
         plt.ylim(0,0.05)
         plt.xlim(2012,2013)
         plt.ylabel('Prevalence')
         plt.xticks([2012,2013], ['2012','2013'])
         plt.legend()

Out[16]: <matplotlib.legend.Legend at 0x10b738c90>
```

At local level changes in prevalence can be more pronounced than at national level, but even with the largest changes in prevalence the new steady state is reached after much less than a year.

1.2 Different testing rates in different populations

We also investigate the sensitivity of the model to different testing rates in subpoulations with different prevalences. This analysis makes use of results reported in Woodhall *Sex. Transm. Infect.* **92**:21-227 (2016) for the proportion of 16-24-year-old men in Natsal-3 reporting different risk behaviours and chlamydia testing and diagnosis in the last year.

Taking each risk factor in turn, we estimate prevalence for each risk level and take the weighted average as an estimate of population prevalence. We also estimate prevalence from the proportion tested and and diagnosed in the whole population, for comparison.

```
In [17]: # analysis by identified risk factors for prevalent infection:
         # age group, deprivation index and lifetime number of sexual partners
         factors = ['Age group', 'Deprivation', 'Age left school',
                     'Age at first heterosexual sex', 'Sexual partners, last year',
                    'New sexual parterns, last year', 'Sexual partners without a condom, last year',
                    'Lifetime sexual partners', 'Condom use at most recent sex',
                    'Concurrent partnerships, last year', 'Binge drinking',
                    'Same sex experience/contact, ever']

         # proportion of those surveyed reporting each risk factor level
         n3_props =[[0.373, 0.627],
                    [0.369,0.182,0.449],
                    [0.754, 0.246],
                    [0.351, 0.261, 0.388],
                    [0.573, 0.187, 0.136, 0.104],
                    [0.421, 0.326, 0.253],
                    [0.331, 0.475, 0.194],
```

```
             [0.527, 0.224, 0.249],
             [0.517, 0.483],
             [0.712, 0.143, 0.145],
             [0.526, 0.202, 0.273],
             [0.08, 0.92]]

# proportion reporting testing in the last year, by risk factor level
n3_test = [[0.404, 0.311],
           [0.345, 0.333, 0.352],
           [0.336, 0.378],
           [0.256, 0.334, 0.453],
           [0.26, 0.403, 0.43, 0.609],
           [0.26, 0.367, 0.463],
           [0.270, 0.343, 0.488],
           [0.253,0.396,0.492],
           [0.335, 0.38],
           [0.329, 0.497, 0.385],
           [0.281, 0.399, 0.432],
           [0.339, 0.424]]

# proportion reporting diagnosis in the last year, by risk factor level
n3_diag = [[0.404*0.047, 0.311*0.067],
           [0.345*0.052, 0.333*0.05, 0.352*0.065],
           [0.336*0.053, 0.378*0.072],
           [0.256*0.028, 0.334*0.047, 0.453*0.078],
           [0.26*0.034, 0.403*0.009, 0.43*0.015, 0.609*0.212],
           [0.26*0.055, 0.367*0.04, 0.463*0.08],
           [0.270*0.018, 0.343*0.049, 0.488*0.109],
           [0.253*0.01,0.396*0.038,0.492*0.123],
           [0.335*0.043, 0.38*0.074],
           [0.329*0.067, 0.497*0.065, 0.385*0.013],
           [0.281*0.035, 0.399*0.04, 0.432*0.098],
           [0.339*0.058, 0.424*0.051]]
```

In [18]:
```
plt.figure(figsize=(8,6))

for j in xrange(len(n3_test)):
    wav = 0
    wav_pos = 0
    plt.plot([0,0.09],[2*(j+1), 2*(j+1)], '0.8')
    for i in xrange(len(n3_test[j])):
        cov = -log(1 - n3_test[j][i])
        adpc = - log(1 - n3_diag[j][i])
        [incsol, scrsol] = fsolve(
            lambda x: [test_diag_fun(x)[0] - cov, test_diag_fun(x)[1] - adpc], [0.09, 0.25]
        )
        prev = 1 - U_fun(incsol*p_asymp,
                         sc + scrsol*p_true_pos,
                         incsol*(1-p_asymp),
                         scrsol*p_true_pos + att_symp*p_true_pos
                        )
        plt.plot(prev, 2*(j+1), 'ob', markerfacecolor='None', markersize=20*sqrt(n3_props[j][i]))
        wav = wav + n3_props[j][i]*prev
        wav_pos = wav_pos + n3_props[j][i]*adpc/cov

    plt.plot(wav, 2*(j+1), 'ob')
    plt.text(0.082, 2*(j+1), factors[j], verticalalignment='center')

# overall
cov = -log(1 - 0.346)
adpc = - log(1 - 0.02)
[incsol, scrsol] = fsolve(
    lambda x: [test_diag_fun(x)[0] - cov, test_diag_fun(x)[1] - adpc], [0.09, 0.25]
)
prev = 1 - U_fun(
    incsol*p_asymp, sc + scrsol*p_true_pos, incsol*(1-p_asymp), scrsol*p_true_pos + att_symp*p_true_pos
    )

plt.plot([prev,prev],[0,100])
```

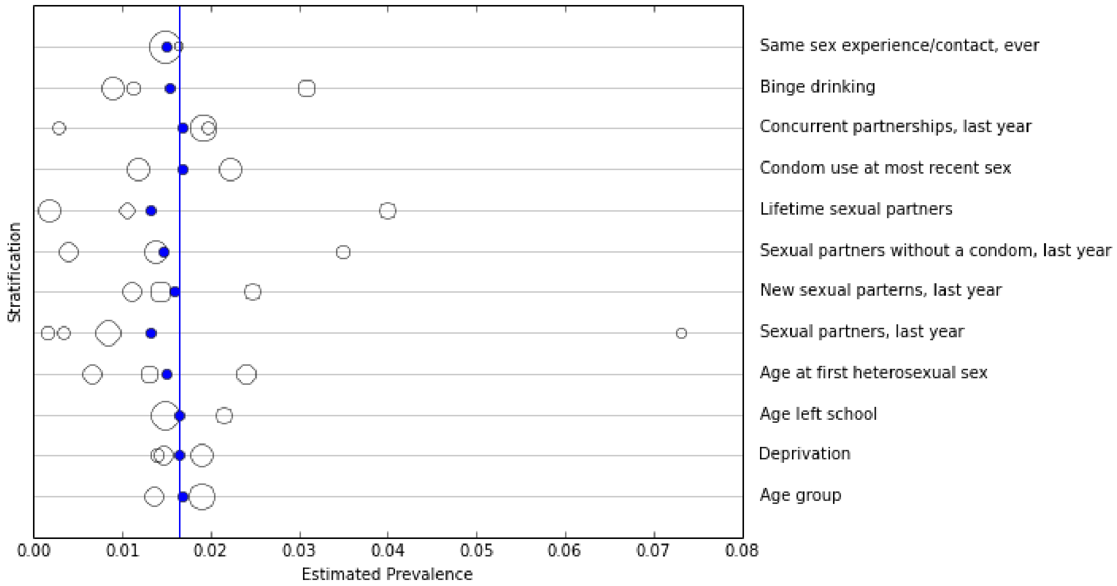

Figure 6: Sensitivity of prevalence estimates in 16-24-year-old men to risk-dependent differences in testing. Hollow markers: risk-level-specific estimates. Marker area is proportional to the proportion of the population in each risk category. Solid markers: weighted mean of level-specific estimates. Vertical line: estimate using aggregated proportions tested and diagnosed.

```
cur_axes = plt.gca()
cur_axes.axes.get_yaxis().set_ticks([])
plt.xlim([0,0.08])
plt.ylim([0,26])
plt.xlabel('Estimated Prevalence')
plt.ylabel('Stratification')
```

Out[18]: <matplotlib.text.Text at 0x10a5f7890>

In this figure, hollow markers show risk-level-specific prevalence estimates and their area represents the proportion of the population in each risk group. Large markers show the weighted average of these level-specific estimates. The solid line shows prevalence estimated from aggregated testing and diagnosis (ie. not stratified by risk). It should be emphasised that due to limitations of the data, the analysis is intended as an illustration of the model's theoretical properties rather than an accurate estimate of prevalence in the different risk categories. The data from Natsal-3 is some of the best available, but nonetheless relies on participants' recall and accurate self-reporting. It was collected at a national level, and equivalent information is not available at a local level for incorporation into local-level prevalence estimates.

Although aggregating across the population does affect prevalence estimates, the differences are small compared with the 1-2% uncertainty which we found in our analyses of the surveillance data.

In [ ]:

Estimating local chlamydia incidence and prevalence using surveillance data: eAppendix 2

Joanna Lewis and Peter White

August 19, 2016

Contents

1 Example: Chlamydia in England, 2012 1
1.1 Sampling for testing and diagnosis rates . . . 1
1.2 Sampling natural history, behavioural and other parameters . . . 3
1.2.1 Test performance . . . 3
1.2.2 Rate of treatment seeking by symptomatic cases . . . 3
1.2.3 Rate of spontaneous clearance of infection . . . 6
1.2.4 Proportion of incident infections asymptomatic . . . 7
1.3 Estimating national prevalence . . . 9
1.4 Symptomatic and asymptomatic diagnoses . . . 12

1 Example: Chlamydia in England, 2012

This example illustrates a method for using chlamydia surveillance data to estimate prevalence. Surveillance data on chlamydia testing and diagnosis rates in England in 2012 were downloaded from: http://www.chlamydiascreening.nhs.uk/ps/data.asp (downloaded 9 February 2016).

	Men			Women		
	15-19 years	20-24 years	Total	15-19 years	20-24 years	Total
Population	1685620	1833395	3519015	1600686	1788156	3388842
Tests	232668	334240	566908	520358	685538	1205896
Diagnoses	15213	33174	48387	42874	45227	88101

Data on sexual behaviour from the third National Study of Sexual Attitudes and Lifestyles (Natsal-3) are available from the UK data service: https://www.ukdataservice.ac.uk/ (downloaded 23 September 2015). These were used to infer 95% confidence intervals for the proportions of men and women, aged 16-19 and 20-24, who were sexually active (see the accompanying R script; note that no 15-year-olds were recruited to Natsal-3). These 95% confidence intervals were in turn used to derive beta-distribution priors for the proportion sexually active within each sex and age group.

1.1 Sampling for testing and diagnosis rates

```
In [1]: import numpy as np
        from numpy import *
        from scipy.stats import beta
        from scipy.optimize import fsolve


        ######################
        # parameters of beta distributions representing the proportion of the population sexually
```

```
# active, by sex and age group
######################

# men, 16-19
[alpha_m_16_19, beta_m_16_19] = fsolve(
    lambda x: array(beta.interval(0.95, x[0], x[1], loc=0, scale=1))
    - (0.6747424, 0.741327698),
    [1,1]
    )
# men, 20-24
[alpha_m_20_24, beta_m_20_24] = fsolve(
    lambda x: array(beta.interval(0.95, x[0], x[1], loc=0, scale=1))
    - (0.8844970, 0.933759842),
    [1,1]
    )
# men, 16-24
[alpha_m_16_24, beta_m_16_24] = fsolve(
    lambda x: array(beta.interval(0.95, x[0], x[1], loc=0, scale=1))
    - (0.8023836019, 0.843403825),
    [1,1]
    )
# women, 16-19
[alpha_f_16_19, beta_f_16_19] = fsolve(
    lambda x: array(beta.interval(0.95, x[0], x[1], loc=0, scale=1))
    - (0.6583593, 0.723554878),
    [1,1]
    )
# women, 20-24
[alpha_f_20_24, beta_f_20_24] = fsolve(
    lambda x: array(beta.interval(0.95, x[0], x[1], loc=0, scale=1))
    - (0.8904135, 0.934417684),
    [1,1]
    )
# women, 16-24
[alpha_f_16_24, beta_f_16_24] = fsolve(
    lambda x: array(beta.interval(0.95, x[0], x[1], loc=0, scale=1))
    - (0.7998634469, 0.837979601),
    [1,1]
    )
```

Next, sample from distributions for the probability of being sexually active, the size of the sexually active population and the testing and diagnosis rates per person per year.

```
In [2]: from scipy.stats import gamma
        from numpy.random import normal
        rs = random.RandomState(12345)

        n_sample = 10000

        # sexually-active populations:
        p_active_m_16_19 = rs.beta(alpha_m_16_19, beta_m_16_19, size=n_sample) # 16-19 yo only
        pop_active_m_15_19 = rs.binomial(1685620, p_active_m_16_19, size=n_sample)

        p_active_m_20_24 = rs.beta(alpha_m_20_24, beta_m_20_24, size=n_sample) # 20-24 yo only
        pop_active_m_20_24 = rs.binomial(1833395, p_active_m_20_24, size=n_sample)

        p_active_m_16_24 = rs.beta(alpha_m_16_24, beta_m_16_24, size=n_sample) # 16-24 yo only
        pop_active_m_15_24 = rs.binomial(3519015, p_active_m_16_24, size=n_sample)

        p_active_f_16_19 = rs.beta(alpha_f_16_19, beta_f_16_19, size=n_sample) # 16-19 yo only
        pop_active_f_15_19 = rs.binomial(1600686, p_active_f_16_19, size=n_sample)

        p_active_f_20_24 = rs.beta(alpha_f_20_24, beta_f_20_24, size=n_sample) # 20-24 yo only
        pop_active_f_20_24 = rs.binomial(1788156, p_active_f_20_24, size=n_sample)

        p_active_f_16_24 = rs.beta(alpha_f_16_24, beta_f_16_24, size=n_sample) # 16-24 yo only
        pop_active_f_15_24 = rs.binomial(3388842, p_active_f_16_24, size=n_sample)

        # testing and diagnosis rates, per person per year
```

```
test_rate_m_15_19 = rs.gamma(232668, 1, size=n_sample)/pop_active_m_15_19
test_rate_m_20_24 = rs.gamma(334240, 1, size=n_sample)/pop_active_m_20_24
test_rate_m_15_24 = rs.gamma(566908, 1, size=n_sample)/pop_active_m_15_24

diag_rate_m_15_19 = rs.gamma(15213, 1, size=n_sample)/pop_active_m_15_19
diag_rate_m_20_24 = rs.gamma(33174, 1, size=n_sample)/pop_active_m_20_24
diag_rate_m_15_24 = rs.gamma(48387, 1, size=n_sample)/pop_active_m_15_24

diag_rate_f_15_19 = rs.gamma(42874, 1, size=n_sample)/pop_active_f_15_19
diag_rate_f_20_24 = rs.gamma(45227, 1, size=n_sample)/pop_active_f_20_24
diag_rate_f_15_24 = rs.gamma(88101, 1, size=n_sample)/pop_active_f_15_24

test_rate_f_15_19 = rs.gamma(520358, 1, size=n_sample)/pop_active_f_15_19
test_rate_f_20_24 = rs.gamma(685538, 1, size=n_sample)/pop_active_f_20_24
test_rate_f_15_24 = rs.gamma(1205896, 1, size=n_sample)/pop_active_f_15_24
```

```
In [3]: print percentile(test_rate_m_15_24,50)
        print percentile(diag_rate_m_15_24,50)
```

```
0.195629009259
0.016699659345
```

1.2 Sampling natural history, behavioural and other parameters

1.2.1 Test performance

Priors for the test performance parameters are beta distributions parameterised directly from literature studies.

```
In [4]: # test performance

        # Horner J. Clin. Microbiol (2005): 32 of 32 infected samples tested +ve
        p_true_pos_m = rs.beta(32+1, 0+1, size=n_sample)
        # Horner J. Clin. Microbiol (2005): 2 of 952 uninfected samples tested +ve
        p_false_pos_m = rs.beta(2+1, 950+1, size=n_sample)
        # Low Health Technol Assess (2007): 129 of 141 infected samples tested +ve
        p_true_pos_f = rs.beta(129+1, 12+1, size=n_sample)
        # Low Health Technol Assess (2007): 4 of 2327 uninfected samples tested +ve
        p_false_pos_f = rs.beta(4+1, 2323+1, size=n_sample)
```

1.2.2 Rate of treatment seeking by symptomatic cases

We use a Metropolis-Hastings algorithm to sample for the rate of treatment following onset of symptoms, assuming a constant hazard of treatment beginning with the onset of symptoms. Data consist of the estimated proportion of GUM clinic patients with symptoms whose symptoms had started < 1, 1-2, 2-4, 4-6 and > 6 weeks previously (Mercer *et al.*, *Sex. Transm. Infect.* **83**:400-405; 2007).

	Proportion	
	Estimate	95% Confidence Interval
$<$ 1 week	26.7%	(14.4, 44.2)%
7-13 days	14.4%	(6.1, 30.2)%
14-27 days	20.8%	(13.3, 31.0)%
4-6 weeks	16.6%	(8.5, 29.9)%
$>$ 6 weeks	21.5%	(5.5, 56.4)%

```
In [5]: # function for calculating likelihood of multinomial data
        %run multinomial_pmf.py
```

```
In [6]: # Find beta distributions corresponding to 95% CIs reported in
        # Mercer Sex. Transm. Infect. (2007) (see table above).

        a = empty(5)
        b = empty(5)
```

```python
    # < 1 week
    [a[0], b[0]] = fsolve(
        lambda x: array(beta.interval(0.95, x[0], x[1], loc=0, scale=1))
        - (0.144, 0.442),
        [1,1]
        )

    # 7-13 days
    [a[1], b[1]] = fsolve(
        lambda x: array(beta.interval(0.95, x[0], x[1], loc=0, scale=1))
        - (0.061, 0.302),
        [1,1]
        )

    # 14-27 days
    [a[2], b[2]] = fsolve(
        lambda x: array(beta.interval(0.95, x[0], x[1], loc=0, scale=1))
        - (0.133, 0.310),
        [1,1]
        )

    # 28-41 days
    [a[3], b[3]] = fsolve(
        lambda x: array(beta.interval(0.95, x[0], x[1], loc=0, scale=1))
        - (0.085, 0.299),
        [1,1]
        )

    # 42 days and over
    [a[4], b[4]] = fsolve(
        lambda x: array(beta.interval(0.95, x[0], x[1], loc=0, scale=1))
        - (0.055, 0.564),
        [1,1]
        )
```

In [7]:
```python
# Metropolis-Hastings to get a sample for rate of treatment

i = 0
att_symp = empty(n_sample+1000) # testing rate per person per year. Allow 1000 extra samples for burn-in
ll = empty(n_sample+1000) # log-likelihood
props = empty([n_sample+1000, 5]) # simulated data, for posterior predictive check
old = 0.04 # starting sample value
new = 0.04 # starting sample value

# simulate probabilities corresponding to data

# proportion expected in each time window
tps = array([0., 7., 14., 28., 42., Inf])
simp_old = exp(-old*tps[:5]) - exp(-old*tps[1:])
simp_new = exp(-new*tps[:5]) - exp(-new*tps[1:])

acc=0.
while i < n_sample+1000: # to do samples for p_test_symp

    new = rs.normal(old, 0.05) # generate a sample from normal distribution

    if new < 0:
        att_symp[i] = old # reject
        ll[i] = -1e10
    else:
        simp_old = exp(-old*tps[:5]) - exp(-old*tps[1:])
        simp_new = exp(-new*tps[:5]) - exp(-new*tps[1:])

        if sum(simp_new > 0) != len(tps) - 1:
            att_symp[i] = old # reject
            ll[i] = -1e10
        else:
            # simulate probabilities corresponding to the data
```

```
            log_ratio = \
                sum(beta.logpdf(simp_new, a, b, loc=0, scale=1)) \
                - sum(beta.logpdf(simp_old, a, b, loc=0, scale=1))

            if log(rs.uniform(0,1)) <  log_ratio:
                att_symp[i] = new # accept
                ll[i] = sum(beta.logpdf(simp_new, a, b, loc=0, scale=1))
                old = new
                acc = acc+1
            else:
                att_symp[i] = old # reject
                ll[i] = sum(beta.logpdf(simp_old, a, b, loc=0, scale=1))

    props[i] = simp_old
    i = i+1

att_symp = att_symp[1000:] # remove burn-in samples
ll = ll[1000:] # log-likelihood

print acc/(n_sample+1000) # print the proportion of samples accepted
print mean(att_symp)*365.25
print array(percentile(att_symp, [2.5, 97.5]))*365.25

att_symp = att_symp*365.25 # convert rate from day^-1 to year^-1
```

```
0.226545454545
14.4054933827
[  8.59839927  22.22498957]
```

In [8]:
```
# Figure 1
# diagnostics and posterior predictive checks

import matplotlib.pyplot as plt
%matplotlib inline

from numpy.random import multinomial

fig = plt.figure(figsize = (10,10))

ax1 = fig.add_subplot(221)
ax1.plot(att_symp, alpha=0.5)

ax2 = fig.add_subplot(222)

ax2.plot(range(43),median(att_symp/365.25)*exp(-median(att_symp/365.25)*array(range(43))),'b')
#plt.plot(range(50),percentile(att_symp, 2.5)*exp(-percentile(att_symp, 2.5)*array(range(50))),'b--')
#plt.plot(range(50),percentile(att_symp, 97.5)*exp(-percentile(att_symp, 97.5)*array(range(50))),'b--')

#ax2.set_ylim([0,0.1])
ax2.set_xlim([0,50])
ax2.errorbar([3.5,10.5,21,35, 46],
            [0.267/7, 0.144/7, 0.208/14, 0.166/14, 0.215/10],
            abs(array([[0.144/7, 0.061/7, 0.133/14, 0.085/14, 0.055/10],
                       [0.442/7, 0.302/7, 0.310/14, 0.299/14, 0.564/10]]
                      ) - array([0.267/7, 0.144/7, 0.208/14, 0.166/14, 0.215/10])
    ), color = 'r', fmt='.')

ax2.plot([0,7,7,14,14,28,28,42], repeat(percentile(props[:,:4],50,0)/array([7,7,14,14]),2), 'b--')
ax2.plot([42,50], repeat(percentile(props[:,4],50,0)/array([10]),2), 'b--')
ax2.fill_between(
    [0,7,7,14,14,28,28,42,42,50],
    repeat(percentile(props,2.5,0)/array([7,7,14,14,10]),2),
    repeat(percentile(props,97.5,0)/array([7,7,14,14,10]),2),
    alpha=0.5
)

ax1.set_xlabel('Sample')
ax1.set_ylabel('Rate of seeking treatment (year^-1)')
```

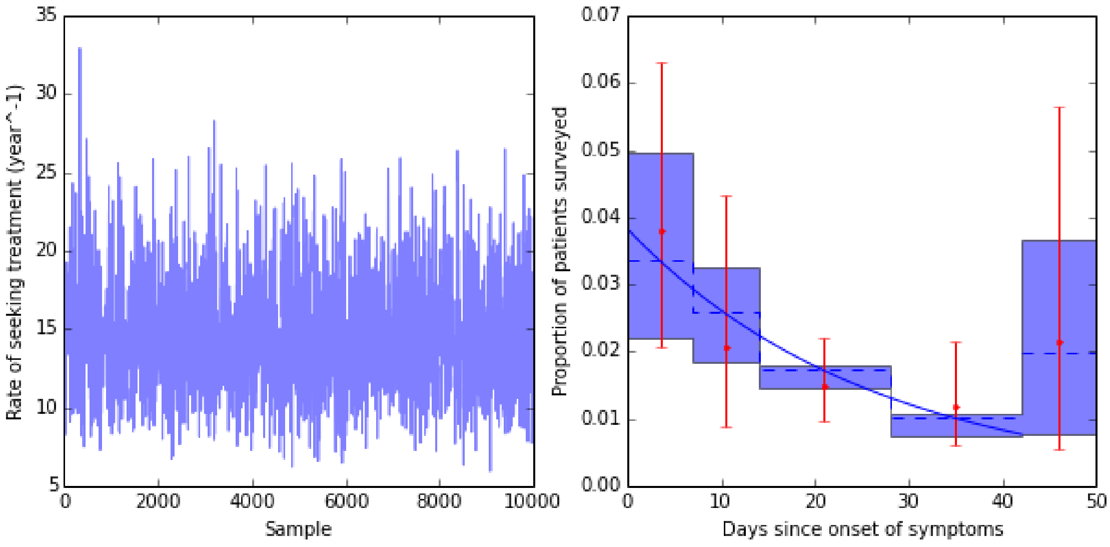

Figure 1: Diagnostic plots for MCMC sampling of treatment seeking rate in symptomatic patients. Left: MCMC chain. Right: posterior predictive check (for description, see text.)

```
        ax2.set_xlabel('Days since onset of symptoms')
        ax2.set_ylabel('Proportion of patients surveyed')
```

Out[8]: <matplotlib.text.Text at 0x1061006d0>

The MCMC chain is illustrated in the left-hand panel, and seems to have converged well.

The right-hand panel shows the probability density of the time between onset of symptoms and attending the GUM clinic where patients were surveyed, to 42 days (solid blue line). The blue shaded area and dashed line show the central 95% and median of simulated histograms for waiting times to clinic, with bins corresponding to time windows reported in the data. The last bin contains all times longer than six weeks and has been divided by 10 (as opposed to the width of the window) to make it readable. For comparison, red error bars show the reported proportions of patients with treatment-seeking times within each time window (estimate and 95% CI), normalised to be on the same scale as the predictions (blue). The good predictive properties of the model are indicated by the agreement between the data, in red, and the posterior predictions in blue.

1.2.3 Rate of spontaneous clearance of infection

Rates of spontaneous clearance of infection in men and women were sampled using MCMC and the STAN software (see accompanying `R` scripts, STAN model files and references), following the model presented by Price *et al.* in *Stat. Med.* **32**:1547-1560.

In [9]:
```
# Figure 2

import csv
sc_m = empty(n_sample) # clearance rate per person per year
with open('stan/chlamydia_two_exponentials_men.csv', 'rU') as m:
    reader = csv.reader(m)
    i=0
    next(reader) # skip the header row
    for row in reader:
        sc_m[i] = row[0]
        i = i+1

sc_f = empty(n_sample) # clearance rate per person per year
with open('stan/chlamydia_two_exponentials_women.csv', 'rU') as f:
    reader = csv.reader(f)
```

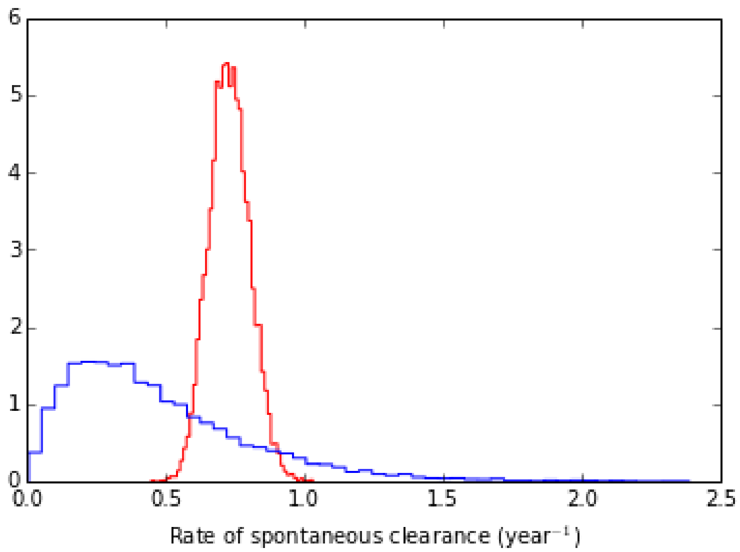

Figure 2: Sampled rates of spontaneous chlamydia clearance in men (blue) and women (red).

```
    i=0
    next(reader) # skip the header row
    for row in reader:
        sc_f[i] = row[0]
        i = i+1

h=plt.hist(sc_f, bins=50, histtype='step', normed=True, color='r')
h=plt.hist(sc_m, bins=50, histtype='step', normed=True, color='b')
plt.xlabel('Rate of spontaneous clearance (year$^{-1}$)')

print 'Mean spontaneous clearance rate in men:', mean(sc_m)
print 'Median (central 95% credible interval) for spontaneous clearance rate in men: \n \t', \
    percentile(sc_m, 50), percentile(sc_m, (2.5,97.5))
print 'Mean spontaneous clearance rate in women:', mean(sc_f)
print 'Median (central 95% credible interval) for spontaneous clearance rate in women:\n \t', \
    percentile(sc_f, 50), percentile(sc_f, (2.5,97.5))
```

```
Mean spontaneous clearance rate in men: 0.469884686724
Median (central 95% credible interval) for spontaneous clearance rate in men:
         0.395824502635 [0.058796284132470837, 1.2738753854360512]
Mean spontaneous clearance rate in women: 0.727835970463
Median (central 95% credible interval) for spontaneous clearance rate in women:
         0.725636286054 [0.59114145020803666, 0.87428940522833309]
```

1.2.4 Proportion of incident infections asymptomatic

Finally, we infer the proportion of infections which are asymptomatic by calibrating to the Natsal-3 prevalence estimates in 16-25-year-old men and women.

```
In [10]: from scipy.stats import beta

         [alpha_prev_m, beta_prev_m] = fsolve(
             lambda x: array(beta.interval(0.95, x[0], x[1], loc=0, scale=1))
             - (0.015, 0.034), # Natsal-3 prevalence in men
             [1,1]
             )
```

```
    prev_m = rs.beta(alpha_prev_m, beta_prev_m, size=n_sample)

    # generate samples for prevalence
    [alpha_prev_f, beta_prev_f] = fsolve(
        lambda x: array(beta.interval(0.95, x[0], x[1], loc=0, scale=1))
        - (0.022, 0.043), # Natsal-3 prevalence in women
        [1,1]
        )

    prev_f = rs.beta(alpha_prev_f, beta_prev_f, size=n_sample)
```

In [11]:
```
# This script also contains the functions linking observed tests, symptomatic/asymptomatic/toal diagnoses,
# incidence, prevalence, screening and other model parameters
# Running it takes a little while because of all the symbolic algebra
%run test_diag_fun.py
```

In [12]:
```
# incidence, screening and proportion of incident infections asymptomatic in men

inc_m = np.zeros(n_sample)
scr_m = np.zeros(n_sample)
p_asymp_m = np.zeros(n_sample)

for i in xrange(n_sample):
    def tmpfun(inc, scr, p_asymp):
        [tr, dr] = test_diag_fun(
            array([
                inc,
                scr,
                1-p_asymp, # proportion of incident infections which are symptomatic
                sc_m[i], # rate of self-clear
                att_symp[i],
                p_true_pos_m[i],
                p_false_pos_m[i]
                ]))
        prev = dyn_fun(
            inc*p_asymp,
            sc_m[i] + scr*p_true_pos_m[i],
            inc*(1-p_asymp),
            scr*p_true_pos_m[i] + att_symp[i]*p_true_pos_m[i]
        )
        return (tr - test_rate_m_15_24[i],
                dr - diag_rate_m_15_24[i],
                prev - prev_m[i])

    [inc_m[i], scr_m[i], p_asymp_m[i]] = fsolve(lambda x: tmpfun(x[0], x[1], x[2]), [0.09, 0.25, 0.9] )
```

In [13]:
```
# Figure 3
# incidence, screening and proportion of incident infections asymptomatic in women

inc_f = np.zeros(n_sample)
scr_f = np.zeros(n_sample)
p_asymp_f = np.zeros(n_sample)

for i in xrange(n_sample):
    def tmpfun(inc, scr, p_asymp):
        [tr, dr] = test_diag_fun(
            array([
                inc,
                scr,
                1-p_asymp, # proportion of incident infections which are symptomatic
                sc_f[i], # rate of self-clear
                att_symp[i],
                p_true_pos_f[i],
                p_false_pos_f[i]
                ]))
        prev = dyn_fun(
            inc*p_asymp,
            sc_f[i] + scr*p_true_pos_f[i],
            inc*(1-p_asymp),
            scr*p_true_pos_f[i] + att_symp[i]*p_true_pos_f[i]
```

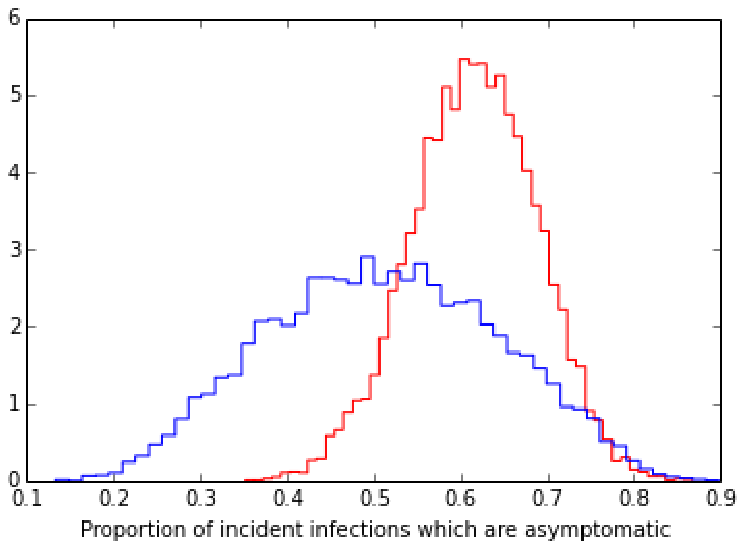

Figure 3: Samples for the proportion of incident infections which are asymptomatic in men (blue) and women (red), calibrated to Natsal-3 prevalence estimates in 16-24-year-olds.

```
            )
            return (tr - test_rate_f_15_24[i],
                    dr - diag_rate_f_15_24[i],
                    prev - prev_f[i])

        [inc_f[i], scr_f[i], p_asymp_f[i]] = fsolve(lambda x: tmpfun(x[0], x[1], x[2]), [0.09, 0.25, 0.9] )
```

```
In [14]: h=plt.hist(p_asymp_f, bins=50, histtype='step', normed=True, color='r')
         h=plt.hist(p_asymp_m, bins=50, histtype='step', normed=True, color='b')
         plt.xlabel('Proportion of incident infections which are asymptomatic')

         print 'Mean proportion asymptomatic in men:', mean(p_asymp_m)
         print 'Median (central 95% credible interval) for proportion asymptomatic in men: \n \t', \
             percentile(p_asymp_m, 50), percentile(p_asymp_m, (2.5,97.5))
         print 'Mean proportion asymptomatic in women:', mean(p_asymp_f)
         print 'Median (central 95% credible interval) for proportion asymptomatic in women:\n \t', \
             percentile(p_asymp_f, 50), percentile(p_asymp_f, (2.5,97.5))
```

```
Mean proportion asymptomatic in men: 0.510862240434
Median (central 95% credible interval) for proportion asymptomatic in men:
        0.509889142897 [0.26393408139570879, 0.75872661515902395]
Mean proportion asymptomatic in women: 0.615291469484
Median (central 95% credible interval) for proportion asymptomatic in women:
        0.616465413009 [0.46763845173602908, 0.75205517865264992]
```

1.3 Estimating national prevalence

The sampled parameter values are now used to infer prevalence in men and women in different age groups.

```
In [15]: from scipy.optimize import fsolve
```

```
In [16]: # men first...
         prev_m_15_19 = np.zeros(n_sample)
         inc_m_15_19 = np.zeros(n_sample)
         scr_m_15_19 = np.zeros(n_sample)

         for i in xrange(n_sample):
```

```
        [inc_m_15_19[i], scr_m_15_19[i]] = fsolve(lambda x: test_diag_fun(concatenate([
                        x, array([
                                1-p_asymp_m[i], # proportion of incident infections which are symptomatic
                                sc_m[i], # rate of self-clear
                                att_symp[i],
                                p_true_pos_m[i],
                                p_false_pos_m[i]
                            ])])) - array([test_rate_m_15_19[i],diag_rate_m_15_19[i]]), [0.09, 0.25])
        prev_m_15_19[i] = dyn_fun(
            inc_m_15_19[i]*p_asymp_m[i],
            sc_m[i] + scr_m_15_19[i]*p_true_pos_m[i],
            inc_m_15_19[i]*(1-p_asymp_m[i]),
            scr_m_15_19[i]*p_true_pos_m[i] + att_symp[i]*p_true_pos_m[i]
        )
```

In [17]:
```
prev_m_20_24 = np.zeros(n_sample)
inc_m_20_24 = np.zeros(n_sample)
scr_m_20_24 = np.zeros(n_sample)

for i in xrange(n_sample):
    [inc_m_20_24[i], scr_m_20_24[i]] = fsolve(lambda x: test_diag_fun(concatenate([
                    x, array([
                            1-p_asymp_m[i], # proportion of incident infections which are symptomatic
                            sc_m[i], # rate of self-clear
                            att_symp[i],
                            p_true_pos_m[i],
                            p_false_pos_m[i]
                        ])])) - array([test_rate_m_20_24[i],diag_rate_m_20_24[i]]), [0.09, 0.25])
    prev_m_20_24[i] = dyn_fun(
        inc_m_20_24[i]*p_asymp_m[i],
        sc_m[i] + scr_m_20_24[i]*p_true_pos_m[i],
        inc_m_20_24[i]*(1-p_asymp_m[i]),
        att_symp[i]*p_true_pos_m[i]
    )
```

In [18]:
```
# ... then women
prev_f_15_19 = np.zeros(n_sample)
inc_f_15_19 = np.zeros(n_sample)
scr_f_15_19 = np.zeros(n_sample)

for i in xrange(n_sample):
    [inc_f_15_19[i], scr_f_15_19[i]] = fsolve(lambda x: test_diag_fun(concatenate([
                    x, array([
                            1-p_asymp_f[i], # proportion of incident infections which are symptomatic
                            sc_f[i], # rate of self-clear
                            att_symp[i],
                            p_true_pos_f[i],
                            p_false_pos_f[i]
                        ])])) - array([test_rate_f_15_19[i],diag_rate_f_15_19[i]]), [0.03, 0.44])
    prev_f_15_19[i] = dyn_fun(
        inc_f_15_19[i]*p_asymp_f[i],
        sc_f[i] + scr_f_15_19[i]*p_true_pos_f[i],
        inc_f_15_19[i]*(1-p_asymp_f[i]),
        scr_f_15_19[i]*p_true_pos_f[i] + att_symp[i]*p_true_pos_f[i]
    )
```

In [19]:
```
prev_f_20_24 = np.zeros(n_sample)
inc_f_20_24 = np.zeros(n_sample)
scr_f_20_24 = np.zeros(n_sample)

for i in xrange(n_sample):
    [inc_f_20_24[i], scr_f_20_24[i]] = fsolve(lambda x: test_diag_fun(concatenate([
                    x, array([
                            1-p_asymp_f[i], # proportion of incident infections which are symptomatic
                            sc_f[i], # rate of self-clear
                            att_symp[i],
                            p_true_pos_f[i],
                            p_false_pos_f[i]
                        ])])) - array([test_rate_f_20_24[i],diag_rate_f_20_24[i]]), [0.03, 0.44])
    prev_f_20_24[i] = dyn_fun(
```

```
            inc_f_20_24[i]*p_asymp_f[i],
            sc_f[i] + scr_f_20_24[i]*p_true_pos_f[i],
            inc_f_20_24[i]*(1-p_asymp_f[i]),
            scr_f_20_24[i]*p_true_pos_f[i] + att_symp[i]*p_true_pos_f[i]
        )
```

In [20]:
```
# Figure 4
# ...and now plot sampled prevalence by age group

fig = plt.figure(figsize = (10,10))

ax1 = fig.add_subplot(221)
h_2012_m_15_19 = ax1.hist(
    prev_m_15_19, bins=20, normed=true, histtype='step', color='cyan', label='15-19 years')
h_2012_m_20_24 = ax1.hist(
    prev_m_20_24, bins=20, normed=true, histtype='step', color='blue', label='20-24 years')
ax1.errorbar(0.001, 25, xerr=[[0],[0.022-0.001]], ecolor='cyan', capsize=10)
ax1.errorbar(0.022, 30, xerr=[[0],[0.052-0.022]], ecolor='blue', capsize=10)
ax1.annotate('18-19 years', [0.001, 25], color='0.5')
ax1.annotate('20-24 years', [0.022, 30], color='0.5')
ax1.set_xlabel('Prevalence')
ax1.set_xlim(0,0.1)
ax1.set_ylim(0,115)
ax1.set_title('Sexually active men')
ax1.legend()

ax2 = fig.add_subplot(222)
h_2012_f_15_19 = ax2.hist(
    prev_f_15_19, bins=20, normed=true, histtype='step', color='fuchsia', label='15-19 years')
h_2012_f_20_24 = ax2.hist(
    prev_f_20_24, bins=20, normed=true, histtype='step', color='r', label='20-24 years')
ax2.errorbar(0.009, 20, xerr=[[0],[0.058-0.009]], ecolor='fuchsia', capsize=10)
ax2.errorbar(0.025, 25, xerr=[[0],[0.086-0.025]], ecolor='fuchsia', capsize=10)
ax2.errorbar(0.017, 30, xerr=[[0],[0.042-0.017]], ecolor='r', capsize=10)
ax2.annotate('16-17 years', [0.009, 20], color='0.5')
ax2.annotate('18-19 years', [0.025, 25], color='0.5')
ax2.annotate('20-24 years', [0.017, 30], color='0.5')
ax2.set_xlabel('Prevalence')
ax2.set_xlim(0,0.1)
ax2.set_ylim(0,115)
ax2.set_title('Sexually active women')
ax2.legend()

print 'Central 95% credible interval for sexually active men, 15-19 years: \n \t', \
    percentile(prev_m_15_19, (2.5, 97.5))
print 'Central 95% credible interval for sexually active men, 20-24 years: \n \t', \
    percentile(prev_m_20_24, (2.5, 97.5))
print 'Central 95% credible interval for sexually active women, 15-19 years: \n \t', \
    percentile(prev_f_15_19, (2.5, 97.5))
print 'Central 95% credible interval for sexually active women, 20-24 years: \n \t', \
    percentile(prev_f_20_24, (2.5, 97.5))
```

```
Central 95% credible interval for sexually active men, 15-19 years:
         [0.011220320772663614, 0.025540614007783301]
Central 95% credible interval for sexually active men, 20-24 years:
         [0.017855916166134259, 0.040426434826233246]
Central 95% credible interval for sexually active women, 15-19 years:
         [0.02590103035920896, 0.050182593529829664]
Central 95% credible interval for sexually active women, 20-24 years:
         [0.019236585373852842, 0.038203370665675189]
```

In these plots, step histograms show the sampled values for prevalence in men and women, by age group. The horizontal bars give 95% confidence intervals for prevalence in comparable age groups, estimated from Natsal-3. They show the agreement between our surveillance-based method and the population-based survey.

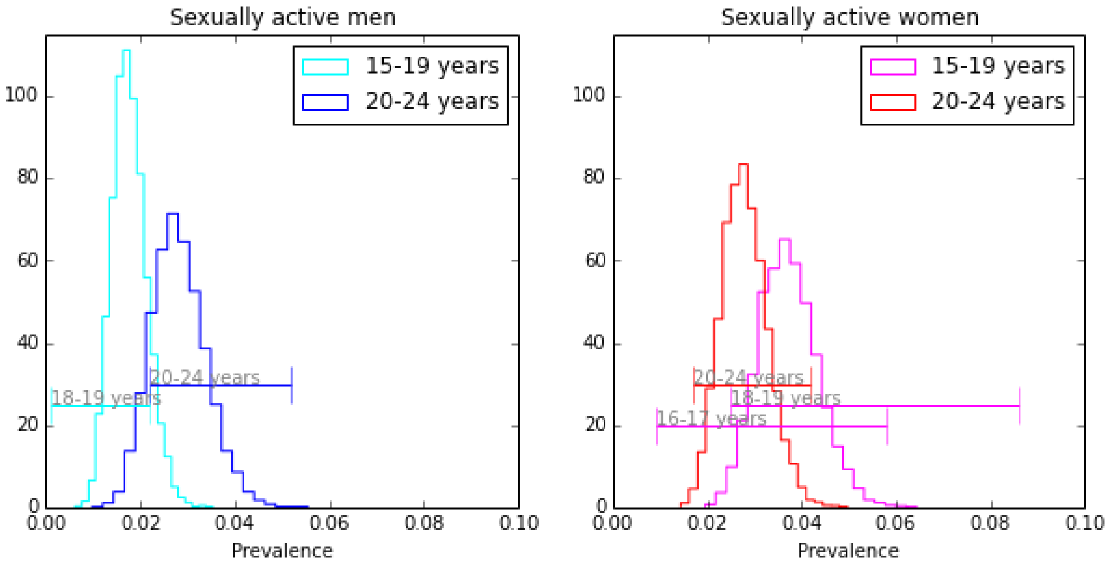

Figure 4: Sampled chlamydia prevalence in men (left) and women (right), by age group. Stepped histograms show samples. Horizontal bars give 95% confidence intervals for prevalence in comparable age groups, estimated from Natsal-3.

1.4 Symptomatic and asymptomatic diagnoses

Although the data does not report the number of diagnoses that were in symptomatic and asymptomatic cases, we can propose different possible numbers of symptomatic and asymptomatic diagnoses and examine the inferences which would have followed in each case.

In [21]:
```
# men first...
prev_m = np.zeros(n_sample)
inc_m = np.zeros(n_sample)
scr_m = np.zeros(n_sample)
p_symp_m = np.zeros(n_sample)

# there were 48387 diagnoses in men aged 15-24
# don't allow all symptomatic or all asymptomatic - messes with gamma distributions
sample_symp_m = ceil(48386*rs.uniform(size = n_sample))
diag_rate_symp_m_15_24 = rs.gamma(sample_symp_m, 1, size=n_sample)/pop_active_m_15_24

sample_asymp_m = 48387 - sample_symp_m
diag_rate_asymp_m_15_24 = rs.gamma(sample_asymp_m, 1, size=n_sample)/pop_active_m_15_24

for i in xrange(n_sample):
    [inc_m[i], scr_m[i], p_symp_m[i]] = fsolve(lambda x: test_diag_sym_asym_fun(concatenate([
                    x, array([
                            sc_m[i], # rate of self-clear
                            att_symp[i],
                            p_true_pos_m[i],
                            p_false_pos_m[i]
                        ])])) - \
                        array([
                            test_rate_m_15_24[i],
                            diag_rate_symp_m_15_24[i],
                            diag_rate_asymp_m_15_24[i]
                            ]),
                        [0.01, 0.3, 0.21])
    prev_m[i] = dyn_fun(
        inc_m[i]*(1-p_symp_m[i]),
        sc_m[i] + scr_m[i]*p_true_pos_m[i],
```

```
        inc_m[i]*p_symp_m[i],
        sc_m[i] + scr_m[i]*p_true_pos_m[i] + att_symp[i]*p_true_pos_m[i])
```

In [22]:
```
# ...then women
prev_f = np.zeros(n_sample)
inc_f = np.zeros(n_sample)
scr_f = np.zeros(n_sample)
p_symp_f = np.zeros(n_sample)

# there were 88101 diagnoses in women aged 15-24
# don't allow all symptomatic or all asymptomatic - messes with gamma distributions
sample_symp_f = ceil(88100*rs.uniform(size = n_sample))
diag_rate_symp_f_15_24 = rs.gamma(sample_symp_f, 1, size=n_sample)/pop_active_f_15_24

sample_asymp_f = 88101 - sample_symp_f
diag_rate_asymp_f_15_24 = rs.gamma(sample_asymp_f, 1, size=n_sample)/pop_active_f_15_24

for i in xrange(n_sample):
    [inc_f[i], scr_f[i], p_symp_f[i]] = fsolve(lambda x: test_diag_sym_asym_fun(concatenate([
                x, array([
                        sc_f[i], # rate of self-clear
                        att_symp[i],
                        p_true_pos_f[i],
                        p_false_pos_f[i]
                    ])])) - \
                    array([
                        test_rate_f_15_24[i],
                        diag_rate_symp_f_15_24[i],
                        diag_rate_asymp_f_15_24[i]
                        ]),
                    [0.01, 0.3, 0.21])
    prev_f[i] = dyn_fun(
        inc_f[i]*(1-p_symp_f[i]),
        sc_f[i] + scr_f[i]*p_true_pos_f[i],
        inc_f[i]*p_symp_f[i],
        sc_f[i] + scr_f[i]*p_true_pos_f[i] + att_symp[i]*p_true_pos_f[i])
```

In [23]:
```
# Figure 5

fig = plt.figure(figsize = (10,12))
xtk_m = [0, 10000, 20000, 30000, 40000] # x-axis ticks for men
xtk_f = [0, 20000, 40000, 60000, 80000] # x-axis ticks for women

ax1 = fig.add_subplot(421)
ax1.plot(100*(1-sample_symp_m/48387), prev_m, ".", alpha = 0.1)
ax1.fill_between([0,50000], 0.015, 0.034, facecolor="b", alpha=0.3)
ax1.plot([40,40],[0,1],"--b")
ax1.plot([20,20],[0,1],"--b")
ax1.set_xlim([0,100])
ax1.set_ylim([0,0.1])
ax1.set_ylabel("Prevalence")
ax1.set_title("Sexually active men, 15-24 years")

ax2 = fig.add_subplot(422)
ax2.plot(100*(1-sample_symp_f/88101), prev_f, ".r", alpha = 0.1)
ax2.fill_between([0,100000], 0.022, 0.043, facecolor="r", alpha=0.3)
ax2.plot([55,55],[0,1],"--r")
ax2.plot([30,30],[0,1],"--r")
ax2.set_xlim([0,100])
ax2.set_ylim([0,0.1])
ax2.set_title("Sexually active women, 15-24 years")

ax3 = fig.add_subplot(423)
ax3.plot(100*(1-sample_symp_m/48387), inc_m, ".", alpha = 0.1)
ax3.plot([40,40],[0,1.2],"--b")
ax3.plot([20,20],[0,1.2],"--b")
ax3.set_xlim([0,100])
ax3.set_ylim([0,0.2])
ax3.set_ylabel("Incidence")
```

```
ax4 = fig.add_subplot(424)
ax4.plot(100*(1-sample_symp_f/88101), inc_f, ".r", alpha = 0.1)
ax4.plot([55,55],[0,1.2],"--r")
ax4.plot([30,30],[0,1.2],"--r")
ax4.set_xlim([0,100])
ax4.set_ylim([0,0.2])

ax5 = fig.add_subplot(425)
ax5.plot(100*(1-sample_symp_m/48387), scr_m, ".", alpha = 0.1)
ax5.plot([40,40],[0,1],"--b")
ax5.plot([20,20],[0,1],"--b")
ax5.set_xlim([0,100])
ax5.set_ylim([0,0.5])
ax5.set_ylabel("Screening")

ax6 = fig.add_subplot(426)
ax6.plot(100*(1-sample_symp_f/88101), scr_f, ".r", alpha = 0.1)
ax6.plot([55,55],[0,1],"--r")
ax6.plot([30,30],[0,1],"--r")
ax6.set_xlim([0,100])
ax6.set_ylim([0,0.5])

ax7 = fig.add_subplot(427)
ax7.plot(100*(1-sample_symp_m/48387), 1 - p_symp_m, ".", alpha = 0.1)
ax7.plot([40,40],[0,1],"--b")
ax7.plot([20,20],[0,1],"--b")
ax7.plot([0,100],[0.76,0.76],"--b")
ax7.plot([0,100],[0.26,0.26],"--b")
ax7.set_xlim([0,100])
ax7.set_ylim([0,1])
ax7.set_xlabel("Proportion of diagnoses asymptomatic (%)")
ax7.set_ylabel("Proportion of incident infections asymptomatic")

ax8 = fig.add_subplot(428)
ax8.plot(100*(1-sample_symp_f/88101), 1 - p_symp_f, ".r", alpha = 0.1)
ax8.plot([55,55],[0,1],"--r")
ax8.plot([30,30],[0,1],"--r")
ax8.plot([0,100],[0.75,0.75],"--r")
ax8.plot([0,100],[0.47,0.47],"--r")
ax8.set_xlim([0,100])
ax8.set_ylim([0,1])
ax8.set_xlabel("Proportion of diagnoses asymptomatic (%)")
```

Out[23]: <matplotlib.text.Text at 0x107dc5510>

The dashed lines are intended as a guide to the eye, to indicate scenarios roughly compatible with the Natsal-3 prevalence estimates. The observed chlamydia prevalence in Natsal-3 would be consistent with around 60-80% of diagnoses in men and 45-70% in women being symptomatic.

In [24]:
```
# Figure 6
# plot top pair only, for figure in paper

fig = plt.figure(figsize = (10,3))

xtk_m = [0, 10000, 20000, 30000, 40000] # x-axis ticks for men
xtk_f = [0, 20000, 40000, 60000, 80000] # x-axis ticks for women

ax1 = fig.add_subplot(121)
ax1.plot(100*(1-sample_symp_m/48387), prev_m, '.', alpha = 0.1)
ax1.fill_between([0,50000], 0.015, 0.034, facecolor='b', alpha=0.3)
ax1.plot([40,40],[0,1],'--b')
ax1.plot([20,20],[0,1],'--b')
ax1.set_xlim([0,100])
ax1.set_ylim([0,0.1])
ax1.set_xlabel('Proportion of diagnoses asymptomatic (%)')
ax1.set_ylabel('Prevalence')
ax1.set_title('Sexually active men, 15-24 years')
```

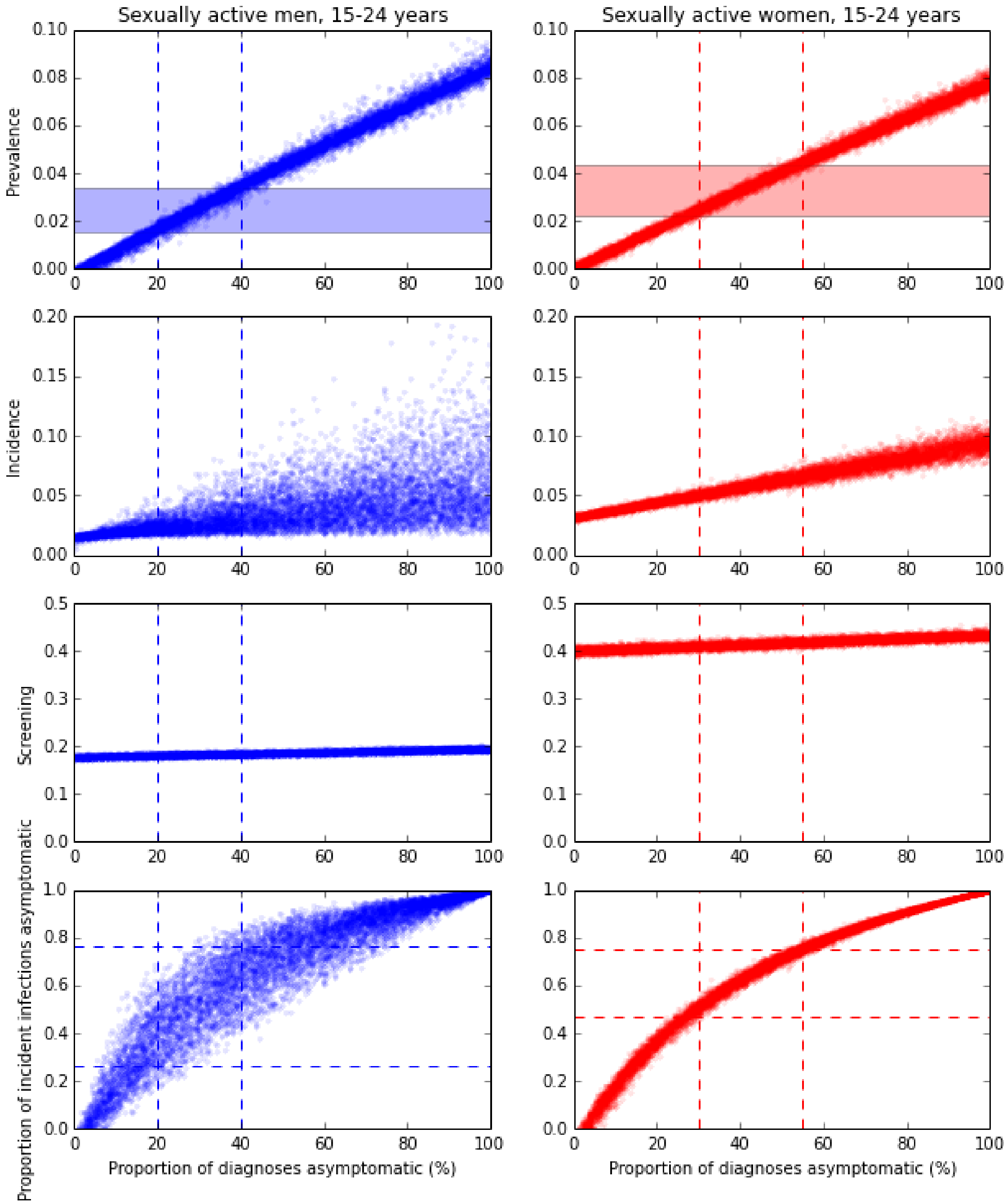

Figure 5: Samples for prevalence, incidence, screening rate and proportion of infections which are symptomatic, assuming different proportions of diagnoses made as a result of symptomms. The dashed lines are intended as a guide to the eye, to indicate scenarios roughly compatible with the Natsal-3 prevalence estimates.

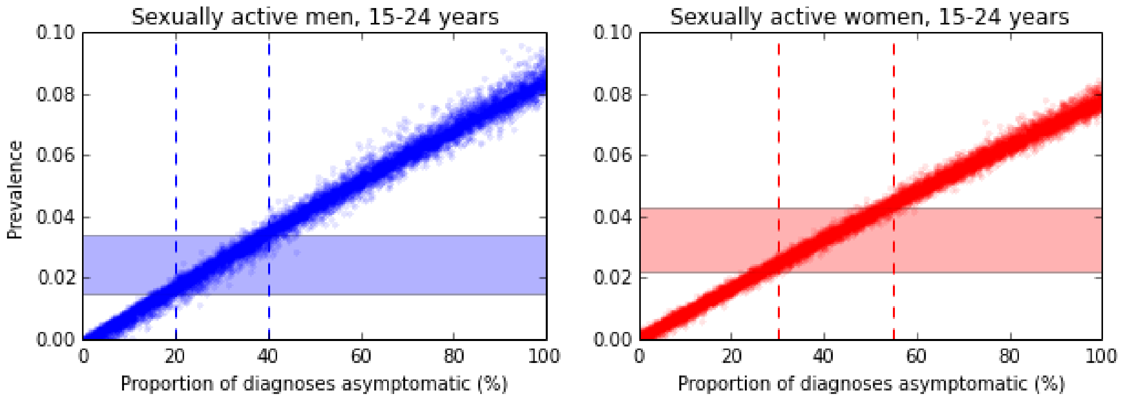

Figure 6: The upper two panels from the previous figure.

```
ax2 = fig.add_subplot(122)
ax2.plot(100*(1-sample_symp_f/88101), prev_f, '.r', alpha = 0.1)
ax2.fill_between([0,100000], 0.022, 0.043, facecolor='r', alpha=0.3)
ax2.plot([55,55],[0,1],'--r')
ax2.plot([30,30],[0,1],'--r')
ax2.set_xlim([0,100])
ax2.set_ylim([0,0.1])
ax2.set_xlabel('Proportion of diagnoses asymptomatic (%)')
ax2.set_title('Sexually active women, 15-24 years')
```

```
Out[24]: <matplotlib.text.Text at 0x1082fd5d0>
```

```
In [ ]:
```

Estimating local chlamydia incidence and prevalence using surveillance data: eAppendix 3

Joanna Lewis and Peter White

August 23, 2016

Contents

1 Local differences in chlamydia prevalence, proportion diagnosed and positivity 1
1.1 Testing and diagnosis rates 2
1.2 Positivity and prevalence 3
1.3 Local differences in prevalence 10
1.4 Prevalence and incidence 19

1 Local differences in chlamydia prevalence, proportion diagnosed and positivity

In this example, we use local numbers of chlamydia tests and diagnoses recorded during 2012 to investigate local differences in incidence, prevalence and screening in men and women.

```
In [1]: # This script also contains the functions linking observed tests, symptomatic/asymptomatic/toal diagnoses,
        # incidence, prevalence, screening and other model parameters
        # Running it takes a little while because of all the symbolic algebra
        %run -i test_diag_fun.py

        # This script provides a function for calculating the likelihood of categorical data.
        %run -i multinomial_pmf.py

        # This script samples model parameters from prior distributions, following the method in england.ipynb.
        %run -i sample_parameters.py
```

Surveillance data on chlamydia testing and diagnosis rates by English local authority (LA) in 2012 were downloaded from: http://www.chlamydiascreening.nhs.uk/ps/data.asp (downloaded 9 February 2016). Numbers of tests and diagnoses were copied into the csv file included with this notebook.

```
In [2]: # now read in the local testing and diagnosis rates
        import pandas as pd
        from pandas import *
        pd.options.mode.chained_assignment = None  # default='warn'

        alldata = pd.read_csv('2012_age_sex_LA.csv')
        alldata = alldata[alldata.la != 'Isles of Scilly'] # remove Scilly Isles because of small numbers
        alldata.index = range(len(alldata))
        print alldata[['la','tests.male.15-19','positives.male.15-19', 'population.male.15-19']][:10]

        # la: Local Authority (Upper Tier)
        # gor: Government Office Region
        # phec: Public Health England Region
        # pher: Public Health England Centre
```

```
la  tests.male.15-19  positives.male.15-19  \
0  Barking and Dagenham              1741                    83
1                Barnet               491                    46
```

```
2              Bexley              631              55
3               Brent             1209              98
4             Bromley             1049              59
5              Camden             1225              91
6      City of London               12               0
7             Croydon             1570             146
8              Ealing             1126              47
9             Enfield              609              44

   population.male.15-19
0                   6672
1                  10694
2                   7850
3                   9809
4                   9289
5                   5915
6                    113
7                  12161
8                   9660
9                  10808
```

Tests, diagnoses and population sizs for men aged 15-19 in ten LAs are printed above, to provide examples of the data used.

1.1 Testing and diagnosis rates

Samples for the testing and diagnosis rates for 16-24-year-old men and women in each LA were generated from gamma distributions based on the data.

```
In [3]: # NB random state (rs) is set in sample_parameters.py, above.

        # set up arrays to store, for each LA:
        test_sample_m = empty([n_sample, len(alldata)]) # testing rate
        test_sample_f = empty([n_sample, len(alldata)])
        diag_sample_m = empty([n_sample, len(alldata)]) # observed diagnosis rate
        diag_sample_f = empty([n_sample, len(alldata)])
        diag_m_la = empty([n_sample, len(alldata)]) # predicted diagnosis rate
        diag_f_la = empty([n_sample, len(alldata)])

        for i in xrange(len(alldata.index)):

            #####
            # men
            #####
            # sample for the testing rate, per sexually active 15-24-year-old
            test_sample_m[:,i] = rs.gamma(alldata['tests.male.total'][i],1,size = n_sample)/ \
                rs.binomial(alldata['population.male.15-19'][i] + alldata['population.male.20-24'][i],
                                p_active_m_16_24, size=n_sample)
            diag_sample_m[:,i] = rs.gamma(alldata['positives.male.total'][i],1,size = n_sample)/ \
                rs.binomial(alldata['population.male.15-19'][i] + alldata['population.male.20-24'][i],
                                p_active_m_16_24, size=n_sample)

            #####
            # women
            #####
            # sample for the testing rate, per sexually active 15-24-year-old
            test_sample_f[:,i] = rs.gamma(alldata['tests.female.total'][i],1,size = n_sample)/ \
                rs.binomial(alldata['population.female.15-19'][i] + alldata['population.female.20-24'][i],
                                p_active_f_16_24, size=n_sample)
            diag_sample_f[:,i] = rs.gamma(alldata['positives.female.total'][i],1,size = n_sample)/ \
                rs.binomial(alldata['population.female.15-19'][i] + alldata['population.female.20-24'][i],
                                p_active_f_16_24, size=n_sample)
```

We now examine the correlation between local proportions tested and diagnosed, for men and women separately.

```
In [4]: # Figure 1:
        # plot testing and diagnosis rates to examine correlation
        import matplotlib.pyplot as plt
        %matplotlib inline

        def plt_ppc(ax, xsample, ysample, index, ci, col, alpha=1):
            # ci is the confidence interval required, as a %
            ax.errorbar(percentile(xsample, 50, index),
                        percentile(ysample, 50, index),
                        xerr=squeeze(
                            array([[percentile(xsample,50, index) - percentile(xsample, (100.-ci)/2, index)],
                                   [percentile(xsample, (100.+ci)/2, index) - percentile(xsample,50, index)]])
                    ),
                        yerr=squeeze(
                            array([[percentile(ysample,50, index) - percentile(ysample, (100.-ci)/2, index)],
                                   [percentile(ysample, (100.+ci)/2, index) - percentile(ysample,50, index)]])
                    ),
                        linestyle = 'None', color = col, alpha=alpha)

        fig = plt.figure(figsize = (10,5))
        ax1 = fig.add_subplot(121)
        ax2 = fig.add_subplot(122)

        plt_ppc(ax1, test_sample_m, diag_sample_m, 0, 95, 'b', alpha=0.3)
        ax1.plot(percentile(test_sample_m, 50, 0), percentile(diag_sample_m, 50, 0), '.b')
        plt_ppc(ax2, test_sample_f, diag_sample_f, 0, 95, 'r', alpha=0.3)
        ax2.plot(percentile(test_sample_f, 50, 0), percentile(diag_sample_f, 50, 0), '.r')

        ax1.set_title('Sexually active men, 15-24'); ax2.set_title('Sexually active women, 15-24')

        ax1.set_xlabel('Proportion tested'); ax2.set_xlabel('Proportion tested')
        ax1.set_ylabel('Proportion diagnosed'); ax2.set_ylabel('Proportion diagnosed')

        ax1.set_xlim([0,1]); ax2.set_xlim([0,1])
        ax1.set_ylim([0,0.1]); ax2.set_ylim([0,0.1])

Out[4]: (0, 0.1)
```

Plotting the proportion of the sexually active population tested for chlamydia against the proportion diagnosed shows clearly the correlation between the two: as more tests are conducted, more infections are discovered. In these (and all subsequent) plots, markers show the median of the sampled distributions, and error bars the 2.5th and 97.5th centiles.

1.2 Positivity and prevalence

Using the sampled proportions tested and diagnosed, we now calculate prevalence in men and women in each LA and then examine the correlation between observed positivity and our estimated prevalence.

```
In [5]: # set up arrays to store, for each LA:
        scr_m_la = empty([n_sample, len(alldata)]) # screening (estimated for each LA separately)
        scr_f_la = empty([n_sample, len(alldata)])
        inc_m_la = empty([n_sample, len(alldata)])  # estimated incidence
        inc_f_la = empty([n_sample, len(alldata)])
        prev_m_la = empty([n_sample, len(alldata)]) # estimated prevalence
        prev_f_la = empty([n_sample, len(alldata)])

        for i in xrange(len(alldata.index)):

            # keep track of whether stuff is happening
            if fmod(i,10)==0:
                print i, alldata.la[i]

            #####
            # men
            #####
```

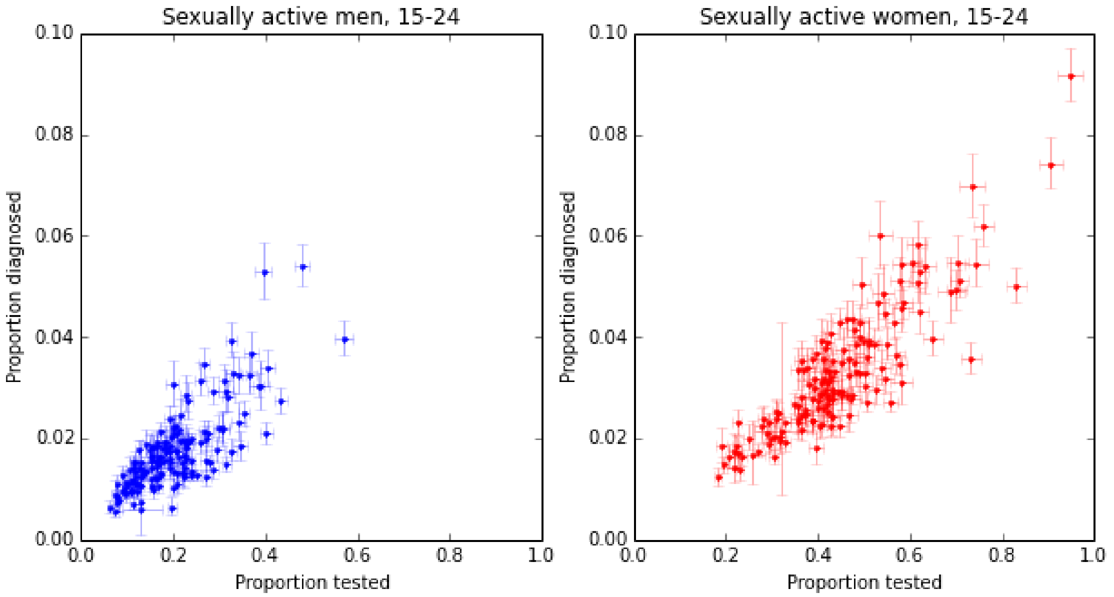

Figure 1: Correlations between the proportions of 16-24-year-old men (left) and women (right) in each local authority who were tested for and diagnosed with chlamydia in 2012.

```
# screening and diagnosis rates
for j in xrange(n_sample):
    # local screening and incidence, given local testing and diagnoses
    [inc_m_la[j,i], scr_m_la[j,i]] = fsolve(lambda x: test_diag_fun(concatenate([
                x, array([
                        1-p_asymp_m[j], # proportion of incident infections which are symptomatic
                        sc_m[j], # rate of self-clear
                        att_symp[j],
                        p_true_pos_m[j],
                        p_false_pos_m[j]
                    ])])) - array([test_sample_m[j,i],diag_sample_m[j,i]]), [0.09, 0.25])
    # local prevalence, calculated from local screening and incidence
    prev_m_la[j,i] = dyn_fun(
        inc_m_la[j,i]*p_asymp_m[j],
        sc_m[j] + scr_m_la[j,i]*p_true_pos_m[j],
        inc_m_la[j,i]*(1-p_asymp_m[j]),
        scr_m_la[j,i]*p_true_pos_m[j] + att_symp[j]*p_true_pos_m[j]
    )

#####
# women
#####

# screening and diagnosis rates
diag_f_la[:,i] = zeros(n_sample)
for j in xrange(n_sample):
    # local screening and incidence, given local testing and diagnoses
    [inc_f_la[j,i], scr_f_la[j,i]] = fsolve(lambda x: test_diag_fun(concatenate([
                x, array([
                        1-p_asymp_f[j], # proportion of incident infections which are symptomatic
                        sc_f[j], # rate of self-clear
                        att_symp[j],
                        p_true_pos_f[j],
                        p_false_pos_f[j]
                    ])])) - array([test_sample_f[j,i],diag_sample_f[j,i]]), [0.09, 0.25])
    # local prevalence, calculated from local screening and incidence
```

```
            prev_f_la[j,i] = dyn_fun(
                inc_f_la[j,i]*p_asymp_f[j],
                sc_f[j] + scr_f_la[j,i]*p_true_pos_f[j],
                inc_f_la[j,i]*(1-p_asymp_f[j]),
                scr_f_la[j,i]*p_true_pos_f[j] + att_symp[j]*p_true_pos_f[j]
            )
```

```
0 Barking and Dagenham
10 Greenwich
20 Kingston upon Thames
30 Waltham Forest
40 Derbyshire
50 Peterborough
60 Solihull
70 Halton
80 Lancashire
90 Wigan
100 South Tyneside
110 Leeds
120 Gloucestershire
130 Bournemouth
140 Medway
150 Wokingham
```

In [6]:
```
# Figure 2

fig = plt.figure(figsize = (10,5))
ax1 = fig.add_subplot(121)
ax2 = fig.add_subplot(122)

# positivity
pos_m_la = diag_sample_m/test_sample_m
pos_f_la = diag_sample_f/test_sample_f

# add to plot
plt_ppc(ax1, prev_m_la, pos_m_la, 0, 95, 'b', alpha=0.2)
ax1.plot(percentile(prev_m_la, 50, 0), percentile(pos_m_la, 50, 0), '.b')
plt_ppc(ax2, prev_f_la, pos_f_la, 0, 95, 'r', alpha=0.2)
ax2.plot(percentile(prev_f_la, 50, 0), percentile(pos_f_la, 50, 0), '.r')

ax1.set_xlim([0,0.08]); ax1.set_ylim([0,0.2])
ax1.set_xlabel('Prevalence in sexually active men 15-24')
ax1.set_ylabel('Positivity')
#ax1.set_title('Sexually active men 15-24')
ax2.set_xlim([0,0.08]); ax2.set_ylim([0,0.2])
ax2.set_xlabel('Prevalence in sexually active women 15-24')
#ax2.set_ylabel('Positivity')
#ax2.set_title('Sexually active women 15-24')
```

Out[6]: <matplotlib.text.Text at 0x11a355d90>

Although there is a positive correlation between prevalence and positivity, positivity is consistently higher because the sample of individuals tested is enriched with infected individuals seeking treatment because of symptoms. There are also a large number of possible pairs of local authorities in which the authority with the lower positivity has the higher prevalence.

The confidence intervals on the positivity and prevalence estimates are wide, but much of this uncertainty stems from weak information on the model's natural history parameters. To understand the correlation better, we estimate Spearman's rho separately for each multivariate sample of model parameters, testing and diagnosis rates:

In [7]:
```
from scipy import stats

# examine the Spearman correlation by sample
```

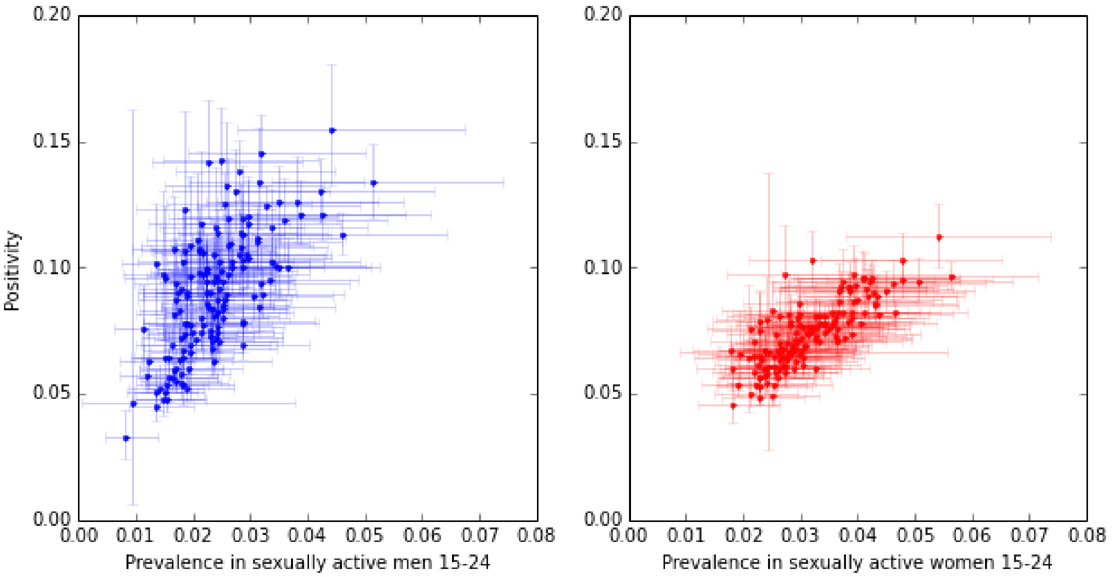

Figure 2: Estimated chlamydia prevalence and observed positivity in 15-24-year-old men (left) and women (right) in English LAs.

```
spearman = empty([shape(pos_m_la)[0],2])
p_val = empty([shape(pos_m_la)[0],2])

for i in xrange(shape(pos_m_la)[0]):
    spearman[i,0] = stats.spearmanr(prev_m_la[i], pos_m_la[i])[0]
    spearman[i,1] = stats.spearmanr(prev_f_la[i], pos_m_la[i])[0]
    p_val[i,0] = stats.spearmanr(prev_m_la[i], pos_m_la[i])[1]
    p_val[i,1] = stats.spearmanr(prev_f_la[i], pos_m_la[i])[1]

# find the (0, 2.5, 25, 50, 97.5, 100)th centiles of the p-values,
# for men (left) and women (right)
percentile(p_val, [0,2.5,25,50,75,97.5,100], axis=0)
```

```
Out[7]: [array([  1.04755350e-173,   2.06354452e-014]),
         array([  1.29156539e-65,   4.90912283e-10]),
         array([  2.89673284e-29,   4.20692935e-08]),
         array([  5.76022005e-21,   3.17066706e-07]),
         array([  5.88944724e-16,   2.00062271e-06]),
         array([  1.49526226e-10,   3.80620189e-05]),
         array([  9.51334495e-07,   2.51852969e-03])]
```

```
In [8]: # Figure 3

        # Set the default color cycle
        import matplotlib as mpl
        mpl.rcParams['axes.color_cycle'] = ['b','r']

        # histogram of the Spearman correlation values
        h=plt.hist(spearman, 20, histtype='step')
        plt.xlabel('Spearman correlation coefficient')
        plt.ylabel('Frequency')
```

```
Out[8]: <matplotlib.text.Text at 0x116876550>
```

For the samples drawn, the correlation between prevalence and positivity (measured by Spearman's ρ) was always positive and statistically significant ($p < 0.05$). However, the correlations - especially for women - were sometimes weak (see histograms).

Next, we examine the relationship between local prevalence and observed diagnoses per capita (without allowing for the fact that not all 15-24-year-olds have become sexually active).

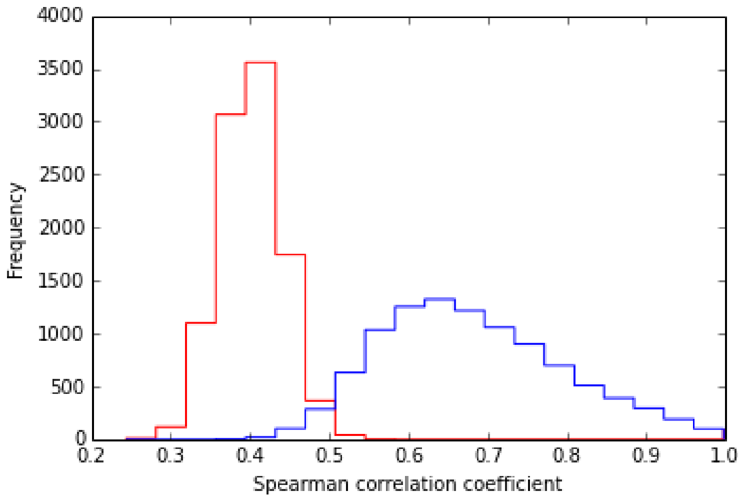

Figure 3: Histogram of Spearman correlations between local positivity and estimated prevalence, calculated at each of 10000 parameter samples.

In [9]:
```
# prevalence vs diagnosis rate

fig = plt.figure(figsize = (10,5))
ax1 = fig.add_subplot(121)
ax2 = fig.add_subplot(122)

ax1.errorbar(
    percentile(prev_m_la, 50, 0),
    alldata['positives.male.total']/(alldata['population.male.15-19'] + alldata['population.male.20-24']),
    xerr=squeeze(
        array([[percentile(prev_m_la,50, 0) - percentile(prev_m_la, 2.5, 0)],
            [percentile(prev_m_la, 97.5, 0) - percentile(prev_m_la,50, 0)]])),
    linestyle='None', color='b', alpha=0.2)
ax1.plot(
    percentile(prev_m_la, 50, 0),
    alldata['positives.male.total']/(alldata['population.male.15-19'] + alldata['population.male.20-24']),
    '.b')
ax2.errorbar(
    percentile(prev_f_la, 50, 0),
    alldata['positives.female.total']/(alldata['population.female.15-19'] + alldata['population.female.20-24']),
    xerr=squeeze(
        array([[percentile(prev_f_la,50, 0) - percentile(prev_f_la, 2.5, 0)],
            [percentile(prev_f_la, 97.5, 0) - percentile(prev_f_la,50, 0)]])),
    linestyle='None', color='r', alpha=0.2)
ax2.plot(
    percentile(prev_f_la, 50, 0),
    alldata['positives.female.total']/(alldata['population.female.15-19'] + alldata['population.female.20-24']),
    '.r')

ax1.set_xlim([0,0.08]); ax1.set_ylim([0,0.08])
ax1.set_xlabel('Prevalence in sexually active men 15-24')
ax1.set_ylabel('ADPC')
#ax1.set_title('Sexually active men 15-24')
ax2.set_xlim([0,0.08]); ax2.set_ylim([0,0.08])
ax2.set_xlabel('Prevalence in sexually active women 15-24')
#ax2.set_ylabel('Positivity')
#ax2.set_title('Sexually active women 15-24')

ax1.plot([0,1],[0,1], 'k')
ax2.plot([0,1],[0,1], 'k')
```

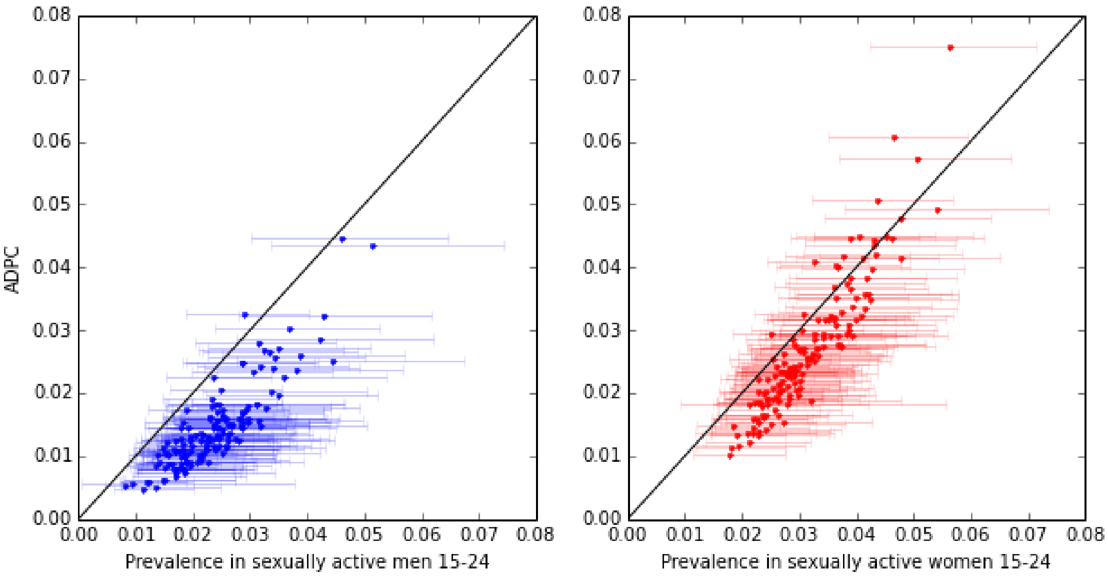

Figure 4: Estimated chlamydia prevalence and observed positivity in 15-24-year-old men (left) and women (right) in English LAs.

```
Out[9]: [<matplotlib.lines.Line2D at 0x11c796510>]
```

Incorporating an estimate for the proportion of each age/sex group who are sexually active, the picture is similar: prevalence is again correlated with, but not equal to, diagnosis rate.

```
In [10]: fig = plt.figure(figsize = (10,5))
         ax1 = fig.add_subplot(121)
         ax2 = fig.add_subplot(122)

         # add to plot
         plt_ppc(ax1, prev_m_la, diag_sample_m, 0, 95, 'b', alpha=0.2)
         ax1.plot(percentile(prev_m_la, 50, 0), percentile(diag_sample_m, 50, 0), '.b')
         plt_ppc(ax2, prev_f_la, diag_sample_f, 0, 95, 'r', alpha=0.2)
         ax2.plot(percentile(prev_f_la, 50, 0), percentile(diag_sample_f, 50, 0), '.r')

         ax1.set_xlim([0,0.08]); ax1.set_ylim([0,0.1])
         ax1.set_xlabel('Prevalence in sexually active men 15-24')
         ax1.set_ylabel('Diagnosis rate')
         #ax1.set_title('Sexually active men 15-24')
         ax2.set_xlim([0,0.08]); ax2.set_ylim([0,0.1])
         ax2.set_xlabel('Prevalence in sexually active women 15-24')
         #ax2.set_ylabel('Positivity')
         #ax2.set_title('Sexually active women 15-24')

         ax1.plot([0,1],[0,1], 'k')
         ax2.plot([0,1],[0,1], 'k')

Out[10]: [<matplotlib.lines.Line2D at 0x11c991c10>]
```

As a consistency check, we calculate weighted averages of the prevalence estimates by LA, and compare these to estimates made from the aggregated national numbers of tests and diagnoses.

```
In [11]: # Figure 4

         pop_active_m = empty([n_sample,len(alldata.index)])
         pop_active_f = empty([n_sample,len(alldata.index)])

         for i in xrange(len(alldata.index)):
             pop_active_m[:,i] = rs.binomial(alldata['population.male.15-19'][i] \
```

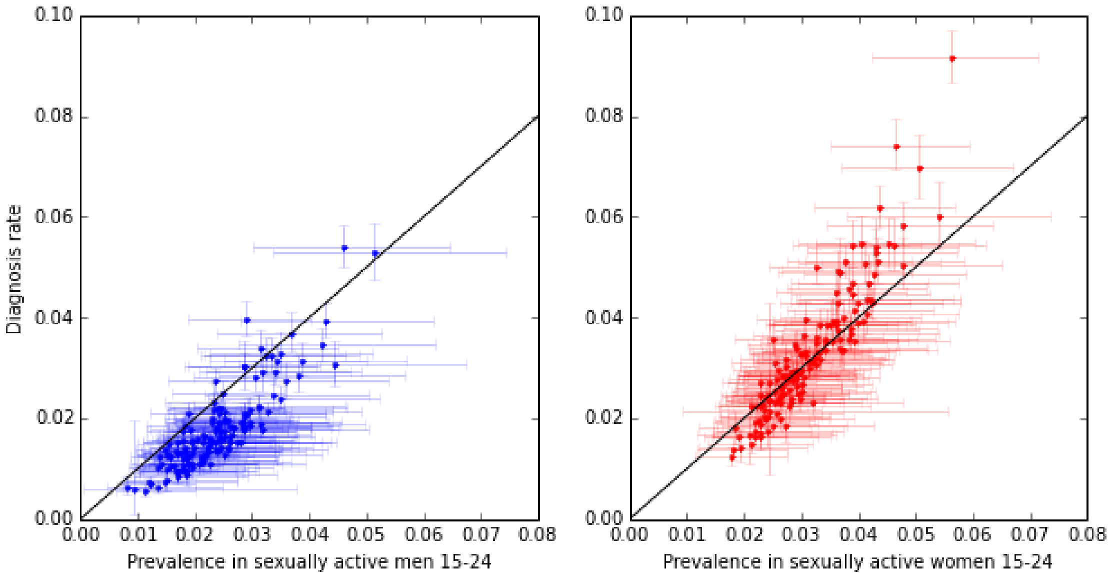

Figure 5:

```
                                + alldata['population.male.20-24'][i],
                          p_active_m_16_24, size=n_sample)
    pop_active_f[:,i] = rs.binomial(alldata['population.female.15-19'][i] \
                                + alldata['population.female.20-24'][i],
                          p_active_f_16_24, size=n_sample)

# testing and diagnosis rates sampled as in england.ipynb
test_rate_m_15_24 = rs.gamma(566908, 1, size=n_sample)/pop_active_m_15_24
diag_rate_m_15_24 = rs.gamma(48387, 1, size=n_sample)/pop_active_m_15_24
test_rate_f_15_24 = rs.gamma(1205896, 1, size=n_sample)/pop_active_f_15_24
diag_rate_f_15_24 = rs.gamma(88101, 1, size=n_sample)/pop_active_f_15_24

inc_m = empty(n_sample); scr_m = empty(n_sample); prev_m = empty(n_sample);
inc_f = empty(n_sample); scr_f = empty(n_sample); prev_f = empty(n_sample);

for j in xrange(n_sample):
    # local screening and incidence, given local testing and diagnoses
    [inc_m[j], scr_m[j]] = fsolve(lambda x: test_diag_fun(concatenate([
                    x, array([
                            1-p_asymp_m[j], # proportion of incident infections which are symptomatic
                            sc_m[j], # rate of self-clear
                            att_symp[j],
                            p_true_pos_m[j],
                            p_false_pos_m[j]
                        ])])) - array([test_rate_m_15_24[j],diag_rate_m_15_24[j]]), [0.09, 0.25])
    # local prevalence, calculated from local screening and incidence
    prev_m[j] = dyn_fun(
        inc_m[j]*p_asymp_m[j],
        sc_m[j] + scr_m[j]*p_true_pos_m[j],
        inc_m[j]*(1-p_asymp_m[j]),
        scr_m[j]*p_true_pos_m[j] + att_symp[j]*p_true_pos_m[j]
    )
    # local screening and incidence, given local testing and diagnoses
    [inc_f[j], scr_f[j]] = fsolve(lambda x: test_diag_fun(concatenate([
                    x, array([
                            1-p_asymp_f[j], # proportion of incident infections which are symptomatic
                            sc_f[j], # rate of self-clear
                            att_symp[j],
                            p_true_pos_f[j],
```

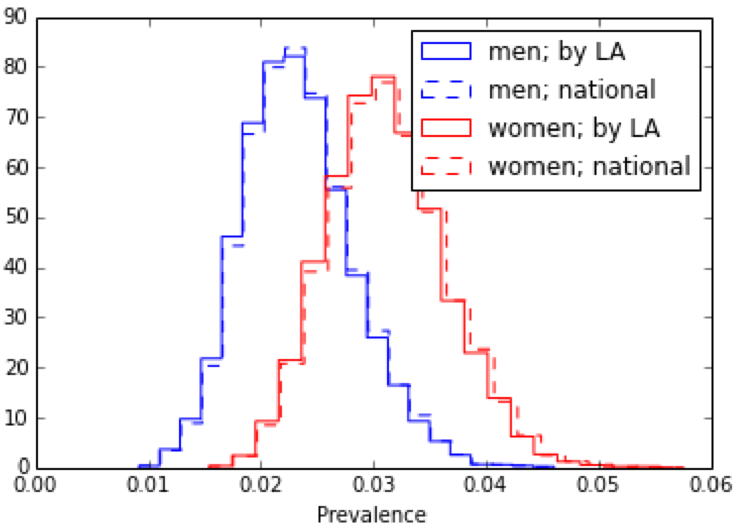

Figure 6: Weighted average of sampled prevalences, by LA (solid lines), and sample based on national numbers of tests and diagnoses (dashed lines).

```
                                p_false_pos_f[j]
                            ])])) - array([test_rate_f_15_24[j],diag_rate_f_15_24[j]]), [0.09, 0.25])
        # local prevalence, calculated from local screening and incidence
        prev_f[j] = dyn_fun(
            inc_f[j]*p_asymp_f[j],
            sc_f[j] + scr_f[j]*p_true_pos_f[j],
            inc_f[j]*(1-p_asymp_f[j]),
            sc_f[j] + scr_f[j]*p_true_pos_f[j] + att_symp[j]*p_true_pos_f[j]
        )

    hm_las=plt.hist(
        sum(prev_m_la*pop_active_m, axis=1)/sum(pop_active_m, axis=1),
        20, histtype='step', normed='true',
        label = 'men; by LA')
    hm_total=plt.hist(prev_m, 20, linestyle='dashed', histtype='step', normed='true', color='b',
        label = 'men; national')
    hf_las=plt.hist(
        sum(prev_f_la*pop_active_f, axis=1)/sum(pop_active_f, axis=1),
        20, histtype='step', color='r', normed='true',
        label = 'women; by LA')
    hf_total=plt.hist(prev_f, 20, linestyle='dashed', histtype='step', normed='true', color='r',
        label = 'women; national')

    plt.xlabel('Prevalence')
    plt.legend()
```

```
Out[11]: <matplotlib.legend.Legend at 0x1167df390>
```

The sampled prevalence distributions are very close, giving confidence in our method.

1.3 Local differences in prevalence

We now use our samples to compare prevalence by local authority.

```
In [12]: shape(prev_m_la)
```

```
Out[12]: (10000, 151)
```

```
In [13]: # Figure 5

         fig = plt.figure(figsize = (12,3))
```

```
ax1 = fig.add_subplot(121)
ax2 = fig.add_subplot(122)

order_m = argsort(percentile(prev_m_la,50,axis=0)) # order by prevalence in men
# Comment-out the next line to plot all LAs. You will also need to adjust axis sizes.
order_m = order_m[append(range(0,5),range(146,151))]
ax1.errorbar(
    y = range(len(order_m)),
    x = (percentile(prev_m_la,50,axis=0))[order_m],
    xerr=array([percentile(prev_m_la[:,order_m],50,axis=0) - percentile(prev_m_la[:,order_m],2.5,axis=0),
            percentile(prev_m_la[:,order_m],97.5,axis=0) - percentile(prev_m_la[:,order_m],50,axis=0)]
            ),
    fmt='.')

ax1.set_ylim(-1, len(order_m)); ax1.set_xlim(0, 0.17)
ax1.set_xlabel('Prevalence in sexually active men 15-24')
ax1.grid(True)
ax1.set_yticklabels([])

print 'Lowest prevalence in men (median sample):', (percentile(prev_m_la,50,axis=0))[order_m[0]]
print 'Highest prevalence in men (median sample):', (percentile(prev_m_la,50,axis=0))[order_m[-1]]

order_f = argsort(percentile(prev_f_la,50,axis=0)) # order by prevalence in women
# Comment-out the next line to plot all LAs. You will also need to adjust axis sizes.
order_f = order_f[append(range(0,5),range(146,151))]
ax2.errorbar(
    y = range(len(order_f)),
    x = (percentile(prev_f_la,50,axis=0))[order_f],
    xerr=array([percentile(prev_f_la[:,order_f],50,axis=0) - percentile(prev_f_la[:,order_f],2.5,axis=0),
            percentile(prev_f_la[:,order_f],97.5,axis=0) - percentile(prev_f_la[:,order_f],50,axis=0)]
            ),
    color='r',fmt='.')

for i in xrange(10):
    ax1.text(0.1, i, alldata.la[order_m[i]])
    ax2.text(0.1, i, alldata.la[order_f[i]])

ax2.set_ylim(-1, len(order_f)); ax2.set_xlim(0, 0.17)
ax2.set_xlabel('Prevalence in sexually active women 15-24')
ax2.grid(True)
ax2.set_yticklabels([])

print 'Lowest prevalence in women (median sample):', (percentile(prev_f_la,50,axis=0))[order_f[0]]
print 'Highest prevalence in women (median sample):', (percentile(prev_f_la,50,axis=0))[order_f[-1]]
```

```
Lowest prevalence in men (median sample): 0.00819755429809
Highest prevalence in men (median sample): 0.0513318116948
Lowest prevalence in women (median sample): 0.0178619507471
Highest prevalence in women (median sample): 0.0561422126184
```

In general, the 95% credible intervals for the highest and lowest LAs do not overlap at all, or only slightly. However there is a large group of over 100 LAs with intermediate prevalence, each with a confidence interval overlapping with all the others in the group. (A plot showing all LAs can be obtained by commenting-out the lines indicated above.) Although there are local differences in prevalence, they are generally small compared with the uncertainty in our estimates. Only in the most extreme cases can differences be clearly resolved. However, the rank order of LAs is robust: we examine consistency in rank order below.

We also plot inferred prevalence against deprivation (rank of average score from the English Indices of Deprivation 2010):

```
In [14]: # Figure 6

         # lookup table for local authority coding in NCSP vs deprivation data
         # Contains National Statistics data © Crown copyright and database right 2016
         district_key = pd.read_csv('LAD12_CTY12_EN_LU.csv')

         # indices of deprivation downloaded from
```

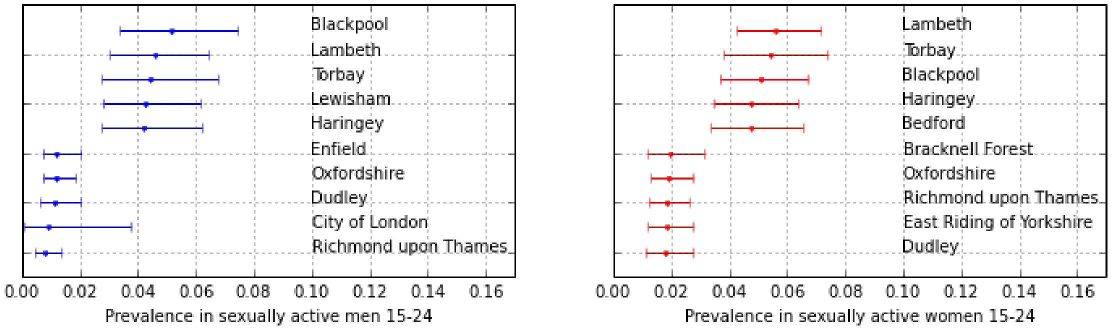

Figure 7: Median and central 95% credible intervals for chlamydia prevalence in the LAs with the five highest and five lowest estimated prevalences for men (left) and women (right).

```
# https://www.gov.uk/government/statistics/english-indices-of-deprivation-2010 1 December 2010
# Contains public sector information licensed under the Open Government Licence v3.0;
# http://www.nationalarchives.gov.uk/doc/open-government-licence/version/3/

# code equivalents downloaded from
# https://data.gov.uk/dataset/local-authority-districts-uk-2012-names-and-codes 4 January 2016
code_key = pd.read_csv('code_equivalents.csv')
deprivation = pd.read_csv('deprivation_indices_2010.csv')

fig = plt.figure(figsize = (10,5))
ax1 = fig.add_subplot(121)
ax2 = fig.add_subplot(122)

quantiles_m = percentile(prev_m_la, [50,2.5,97.5], 0)
quantiles_f = percentile(prev_f_la, [50,2.5,97.5], 0)

for i in deprivation.index:

    old_code = deprivation[u'LA CODE'][i]
    new_code = code_key['Current code'][code_key['Former code'] == old_code].tolist()[0]

    # special case for Northumberland, because a new code was allocated when boundaries changed:
    if new_code == 'E06000048': # Northumberland
        new_code = 'E06000057'

    if new_code in alldata.la_code.tolist(): # if LA can be found in NCSP data using new code
        ax1.plot(deprivation[u'Rank of Average Score'][i],
                quantiles_m[0][where(alldata.la_code == new_code)],
                '.b')
        ax1.errorbar(deprivation[u'Rank of Average Score'][i],
                quantiles_m[0][where(alldata.la_code == new_code)],
                yerr = array([(quantiles_m[0]-quantiles_m[1])[where(alldata.la_code == new_code)],
                             (quantiles_m[2]-quantiles_m[0])[where(alldata.la_code == new_code)]]),
                color='b', alpha=0.2)
        ax2.plot(deprivation[u'Rank of Average Score'][i],
                quantiles_f[0][where(alldata.la_code == new_code)],
                '.r')
        ax2.errorbar(deprivation[u'Rank of Average Score'][i],
                quantiles_f[0][where(alldata.la_code == new_code)],
                yerr = array([(quantiles_f[0]-quantiles_f[1])[where(alldata.la_code == new_code)],
                             (quantiles_f[2]-quantiles_f[0])[where(alldata.la_code == new_code)]]),
                color='r', alpha=0.2)
    elif old_code in district_key['LAD12CDO'].tolist(): # if LA can be found in list of districts
        new_code = district_key['CTY12CD'][district_key['LAD12CDO']==old_code].tolist()[0]
        # special case for Gateshead, because a new code was allocated when boundaries changed:
        if old_code == '00CH':
            new_code = 'E08000037'
```

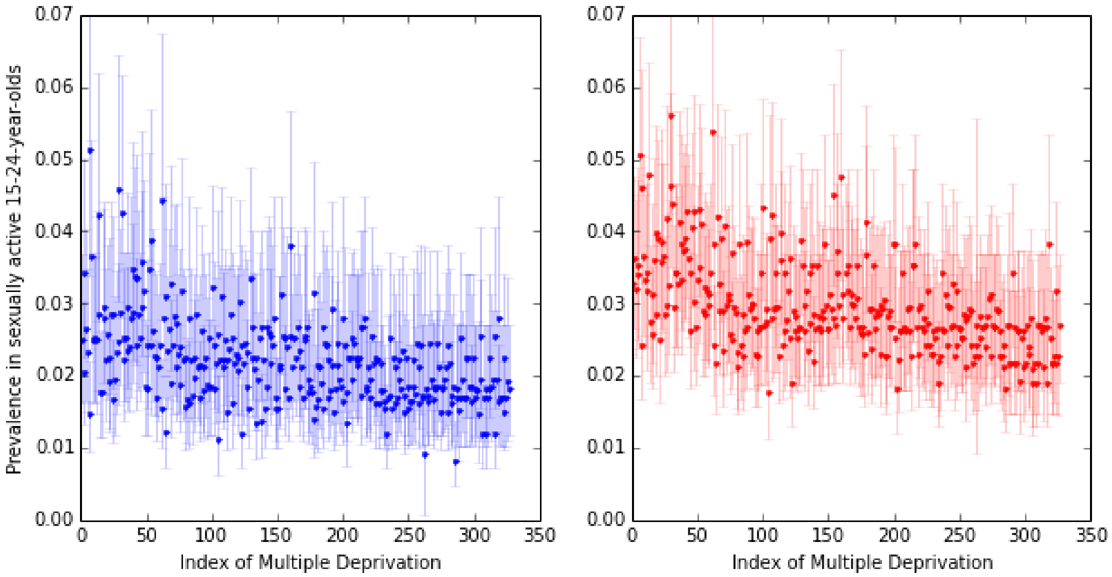

Figure 8: Estimated chlamydia prevalence with rank of average deprivation score (lowest rank is most deprived district), for men (left) and women (right) aged 15-24. Points and error bars give median and central 95% credible interval sampled prevalence.

```
            ax1.plot(deprivation[u'Rank of Average Score'][i],
                quantiles_m[0][where(alldata.la_code == new_code)],
                '.b')
            ax1.errorbar(deprivation[u'Rank of Average Score'][i],
                    quantiles_m[0][where(alldata.la_code == new_code)],
                    yerr = array([(quantiles_m[0]-quantiles_m[1])[where(alldata.la_code == new_code)],
                                 (quantiles_m[2]-quantiles_m[0])[where(alldata.la_code == new_code)]]),
                    color='b', alpha=0.2)
            ax2.plot(deprivation[u'Rank of Average Score'][i],
                quantiles_f[0][where(alldata.la_code == new_code)],
                '.r')
            ax2.errorbar(deprivation[u'Rank of Average Score'][i],
                    quantiles_f[0][where(alldata.la_code == new_code)],
                    yerr = array([(quantiles_f[0]-quantiles_f[1])[where(alldata.la_code == new_code)],
                                 (quantiles_f[2]-quantiles_f[0])[where(alldata.la_code == new_code)]]),
                    color='r', alpha=0.2)
        else:
            # Scilly Isles not plotted because excluded due to low numbers
            print 'no', deprivation[u'LA NAME'][i], old_code, new_code

ax1.set_xlim([0,350]); ax1.set_ylim([0,0.07])
ax1.set_xlabel('Index of Multiple Deprivation')
ax1.set_ylabel('Prevalence in sexually active 15-24-year-olds')
#ax1.set_title('Sexually active men 15-24 years')
ax2.set_xlim([0,350]); ax2.set_ylim([0,0.07])
ax2.set_xlabel('Index of Multiple Deprivation')
#ax2.set_ylabel('Prevalence')
#ax2.set_title('Sexually active women 15-24 years')
```

```
no Isles of Scilly 00HF E06000053
```

```
Out[14]: <matplotlib.text.Text at 0x11e92b510>
```

The pattern shown, of higher prevalence in more deprived areas, agrees with primary analysis of Natsal-3 (Sonnenberg *et al.*, 2013) which identified index of multiple deprivation quintile as a risk factor for chlamydia infection.

We can also show local prevalence on a map:

In [15]:
```
# Figure 7

import shapefile
import matplotlib.pyplot as plt
import matplotlib.patches as patches
from matplotlib.patches import Polygon
from matplotlib.collections import PatchCollection
from mpl_toolkits.axes_grid1.inset_locator import inset_axes

sf = shapefile.Reader(
    "County_and_unitary_authorities_E+W_2013_Boundaries_Generalised_Clipped/CTYUA_DEC_2013_EW_BGC"
    )
recs    = sf.records()
shapes  = sf.shapes()

blues = plt.get_cmap('Blues')  # this returns a colormap
reds = plt.get_cmap('Reds')  # this returns a colormap

key_ys = array([5.2, 5.6, 6, 6.4, 6.8])*10**5 # y-co-ordinates for key
key_labels = ['lowest quintile','2nd quintile','3rd quintile','4th quintile','highest prevalence quintile']

fig = plt.figure(figsize = (10,5))
ax1 = fig.add_subplot(121)
ains1 = inset_axes(ax1, width='40%', height='30%', loc=6)
ax2 = fig.add_subplot(122)
ains2 = inset_axes(ax2, width='40%', height='30%', loc=6)

n_quantile = 5 # how many different colours do you want to plot?

def tickpar(ax):
    ax.tick_params(
        axis='both', # changes apply to
        which='both', # both major and minor ticks are affected
        bottom='off', # ticks along the bottom edge are off
        top='off', # ticks along the top edge are off
        left='off',
        right='off',
        labelbottom='off',
        labelleft='off') # labels along the left edge are off

#######################
# plot prevalence in men
#######################

cNorm  = plt.Normalize(vmin=0, vmax=n_quantile)
scalarMap = plt.cm.ScalarMappable(norm=cNorm, cmap=blues)
colors = argsort(percentile(prev_m_la,50,0))
ranks = argsort(colors)
#patches = []

for nshp in alldata.index:


    # code for this LA
    thiscode = alldata.la_code[nshp]
    # index to find the right shape file for this la:
    shpin = where( map(lambda x: thiscode == x, [recs[i][0] for i in range(len(recs))]) )
    shpin = int(shpin[0])

    ptchs   = []
    ptchs_l   = [] # for london
    pts     = array(shapes[shpin].points)
    prt     = shapes[shpin].parts
    par     = list(prt) + [pts.shape[0]]

    colorVal = scalarMap.to_rgba(n_quantile*ranks[nshp]/151)
```

```
    for pij in xrange(len(prt)):
        ptchs.append(Polygon(pts[par[pij]:par[pij+1]]))

        p = PatchCollection(ptchs, facecolor=colorVal, edgecolor='k', linewidth=0.1)
        p.set_clim([0,151])
        ax1.add_collection(p)

        if alldata.gor[nshp] == 'london':
            p = PatchCollection(ptchs, facecolor=colorVal, edgecolor='k', linewidth=0.1)
            ains1.add_collection(p)

for i in xrange(5):
    ax1.add_patch(patches.Rectangle((0.2*10**5, key_ys[i]), 0.25*10**5, 0.25*10**5, fc=blues(0.2*i)))
    ax1.text(0.6*10**5, key_ys[i], key_labels[i])

ax1.text(0.6*10**5, 6.8*10**5, 'highest prevalence quintile')
ax1.text(0.6*10**5, 6.4*10**5, '4th quintile')
ax1.text(0.6*10**5, 6*10**5, '3rd quintile')
ax1.text(0.6*10**5, 5.6*10**5, '2nd quintile')
ax1.text(0.6*10**5, 5.2*10**5, 'lowest quintile')

ax1.set_xlim(0, 0.7*10**6)
ax1.set_ylim(0, 0.7*10**6)
ax1.set_aspect('equal', 'datalim')
tickpar(ax1)
ains1.set_xlim(0.5*10**6, 0.565*10**6)
ains1.set_ylim(1.55*10**5, 2.05*10**5)
ains1.set_aspect('equal', 'datalim')
tickpar(ains1)
p = PatchCollection(ptchs, cmap=blues)
p = PatchCollection(ptchs_l, cmap=blues)

#######################
# plot prevalence in women
#######################

cNorm  = plt.Normalize(vmin=0, vmax=n_quantile)
scalarMap = plt.cm.ScalarMappable(norm=cNorm, cmap=reds)
colors = argsort(percentile(prev_f_la,50,0))
ranks = argsort(colors)
#patches = []

for nshp in alldata.index:

    # code for this LA
    thiscode = alldata.la_code[nshp]
    # index to find the right shape file for this la:
    shpin = where( map(lambda x: thiscode == x, [recs[i][0] for i in range(len(recs))]) )
    shpin = int(shpin[0])

    ptchs   = []
    ptchs_l   = []
    pts     = array(shapes[shpin].points)
    prt     = shapes[shpin].parts
    par     = list(prt) + [pts.shape[0]]

    colorVal = scalarMap.to_rgba(n_quantile*ranks[nshp]/151)

    for pij in xrange(len(prt)):
        ptchs.append(Polygon(pts[par[pij]:par[pij+1]]))

        p = PatchCollection(ptchs, facecolor=colorVal, edgecolor='k', linewidth=0.1)
        p.set_clim([0,151])
        ax2.add_collection(p)

        if alldata.gor[nshp] == 'london':
            p = PatchCollection(ptchs, facecolor=colorVal, edgecolor='k', linewidth=0.1)
            ains2.add_collection(p)
```

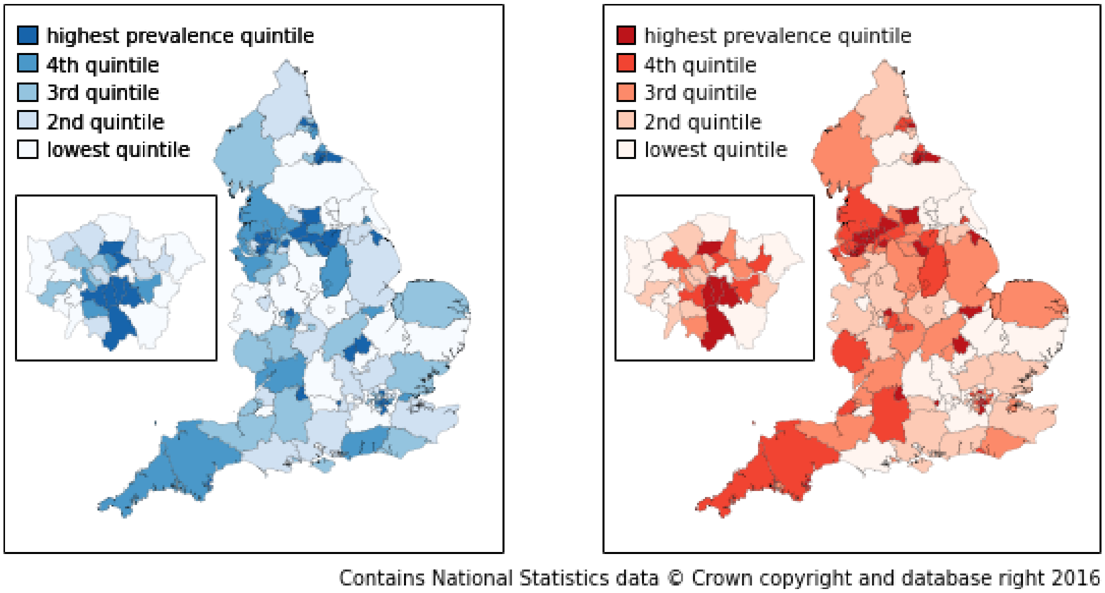

Figure 9: English local authorities, coloured by quintile for estimated chlamydia prevalence in men (left) and women (right). The inset panel shows the London boroughs.

```
for i in xrange(5):
    ax2.add_patch(patches.Rectangle((0.2*10**5, key_ys[i]), 0.25*10**5, 0.25*10**5, fc=reds(0.2*i)))
    ax2.text(0.6*10**5, key_ys[i], key_labels[i])

ax2.set_xlim(0, 0.7*10**6)
ax2.set_ylim(0, 0.7*10**6)
ax2.set_aspect('equal', 'datalim')
ains2.set_xlim(0.5*10**6, 0.565*10**6)
ains2.set_ylim(1.55*10**5, 2.05*10**5)
ains2.set_aspect('equal', 'datalim')
tickpar(ax2)
tickpar(ains2)
p = PatchCollection(ptchs, cmap=reds)

# Crown Copyright statement required by ONS.
fig.text(0.9,0.1,
    u'Contains National Statistics data \N{COPYRIGHT SIGN} Crown copyright and database right 2016',
    ha='right', va='top')
# alternative, if plotting for men only
#fig.text(0.48,0.1,
#    u'Contains National Statistics data \n\N{COPYRIGHT SIGN} Crown copyright and database right 2016',
#    ha='right', va='top')
```

Out[15]: <matplotlib.text.Text at 0x123da0290>

A proportion of the uncertainty in absolute prevalence values is due to uncertainty in model parameters that do not vary across LAs; to make comparisons of relative prevalence across LAs while controlling for this uncertainty in parameters, we compare the prevalence calculated for each LA, at each sampled set of model parameters.

In [16]:
```
# Figure 8

import matplotlib.colors as colors
import matplotlib.cm as cmx

fig = plt.figure(figsize = (10,5))
```

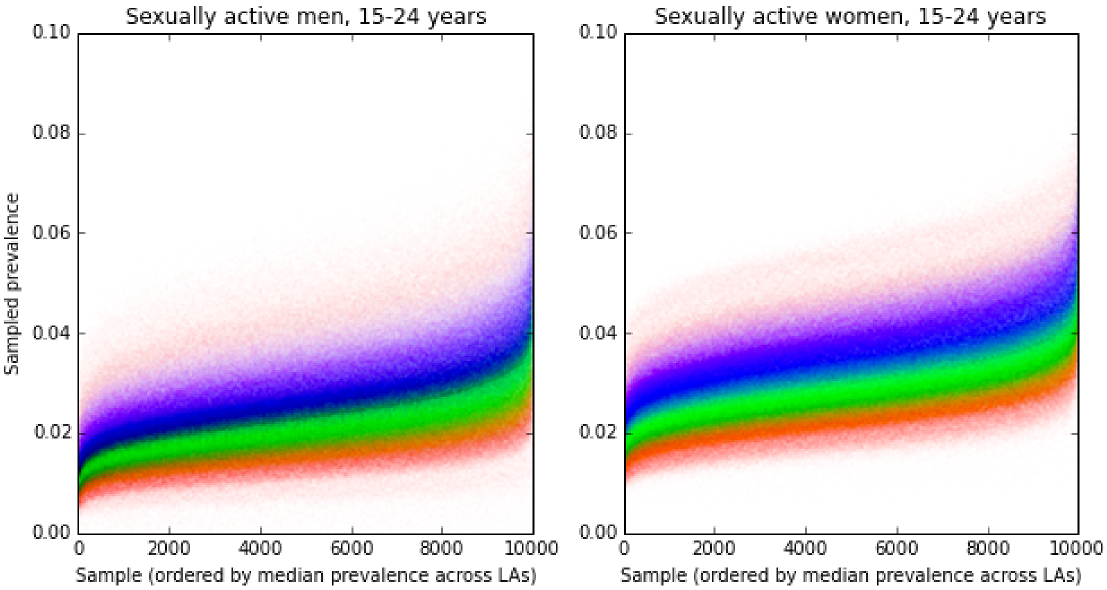

Figure 10: All prevalence samples, for all LA. Horizontal position indicates whether sampled parameter values generally estimated prevalence to be high or low. Vertical position gives the prevalence sampled. Colour (unique to each LA) corresponds to median sampled prevalence for that authority.

```
ax1 = fig.add_subplot(121)
ax2 = fig.add_subplot(122)

# sort samples
prev_m_la = prev_m_la[argsort(percentile(prev_m_la,50,1)),:]
prev_f_la = prev_f_la[argsort(percentile(prev_f_la,50,1)),:]

rb = plt.get_cmap('gist_rainbow')  # this returns a colormap
ax1.set_color_cycle(rb(array(range(151))/151.))
p1=ax1.plot(
    range(n_sample),
    prev_m_la[:,argsort(percentile(prev_m_la,50,0))],
    '.', markersize=0.01,alpha=0.5)
ax2.set_color_cycle(rb(array(range(151))/151.))
p2=ax2.plot(
    range(n_sample),
    prev_f_la[:,argsort(percentile(prev_f_la,50,0))],
    '.', markersize=0.01,alpha=0.5)

ax1.set_xlabel('Sample (ordered by median prevalence across LAs)')
ax1.set_ylabel('Sampled prevalence')
ax1.set_title('Sexually active men, 15-24 years')
ax1.set_ylim([0,0.1])
ax2.set_xlabel('Sample (ordered by median prevalence across LAs)')
ax2.set_title('Sexually active women, 15-24 years')
ax2.set_ylim([0,0.1])
```

```
Out[16]: (0, 0.1)
```

In each panel (left, men; right, women) one dot represents one sampled prevalence in one local authority. Its position on the x-axis corresponds to one set of sampled parameters (Table 2 in the main text) and indicates whether this set generally estimated prevalence to be low, intermediate or high by ordering for the median prevalence across LAs. The position on the y-axis is the sampled prevalence. The colour is unique to each LA, and determined on a colour scale according to that LA's median sampled prevalence so that low-prevalence LAs are red and high-prevalence are violet.

The samples for each LA form a band – indicating that rank of prevalence is largely preserved across samples. The fact that the bands overlap shows that there is some swapping of rank order – this is due to uncertainty in the rate of testing and diagnosis. The y-range over which the band moves as it goes from left to right is at least as great as the thickness of the band itself, showing that uncertainty in the model parameters in Table 2 contributes at least as much variation in the final sample as does uncertainty in the testing and diagnosis rates. Improving estimates of natural history and behaviour parameters would improve prevalence estimates.

Another approach to examing the same question is shown below:

In [17]:
```
# Figure 9

n_quantiles = 5 # choose how many bands you'd like to plot

fig = plt.figure(figsize = (10,3))
ax1 = fig.add_subplot(121)
ax2 = fig.add_subplot(122)

# sort las
prev_m_la_s = prev_m_la[:,argsort(percentile(prev_m_la,50,0))]
prev_f_la_s = prev_f_la[:,argsort(percentile(prev_f_la,50,0))]

# men
blues = plt.get_cmap('Blues')
quantiles = n_quantiles*argsort(prev_m_la_s,axis=1)/151
sizes = [bincount(quantiles[:,i], minlength=n_quantiles) for i in range(151)]
bottoms = zeros(151)
for i in xrange(n_quantiles):
    ax1.bar(range(151), array([sizes[j][i] for j in range(151)])/float(n_sample),
            1,
            bottoms,
            color=blues((0.+i)/n_quantiles), edgecolor='None')
    bottoms = bottoms + array([sizes[j][i] for j in range(151)])/float(n_sample)

# these labels are positioned for quintiles
ax1.annotate('first quintile', [15, 0.6], rotation = 'vertical')
ax1.annotate('second quintile', [45, 0.6], rotation = 'vertical')
ax1.annotate('third quintile', [75, 0.6], rotation = 'vertical')
ax1.annotate('fourth quintile', [110, 0.6], rotation = 'vertical', color='0.9')
ax1.annotate('fifth quintile', [135, 0.6], rotation = 'vertical', color='0.9')

ax1.set_xlim([0,151])
ax1.set_ylim([0,1])
ax1.set_xlabel('LA (ordered by median sampled prevalence)')
ax1.set_ylabel('Proportion of prevalence samples in each quintile')
#ax1.set_title('Sexually active men, 15-24 years')

# how many quintiles are occupied >5% of the time?
#howmany = [sum(sizes[i] >= 0.05*n_sample) for i in range(151)]
#print {x:howmany.count(x)/151. for x in howmany}

# women
reds = plt.get_cmap('Reds')
quantiles = n_quantiles*argsort(prev_f_la_s,axis=1)/151
sizes = [bincount(quantiles[:,i], minlength=n_quantiles) for i in range(151)]
bottoms = zeros(151)
for i in xrange(n_quantiles):
    ax2.bar(range(151),
            array([sizes[j][i] for j in range(151)])/float(n_sample),
            1,
            bottoms,
            color=reds((0.+i)/n_quantiles), edgecolor='None')
    bottoms = bottoms + array([sizes[j][i] for j in range(151)])/float(n_sample)

# these labels are positioned for quintiles
ax2.annotate('first quintile', [15, 0.6], rotation = 'vertical')
ax2.annotate('second quintile', [45, 0.6], rotation = 'vertical')
ax2.annotate('third quintile', [75, 0.6], rotation = 'vertical')
```

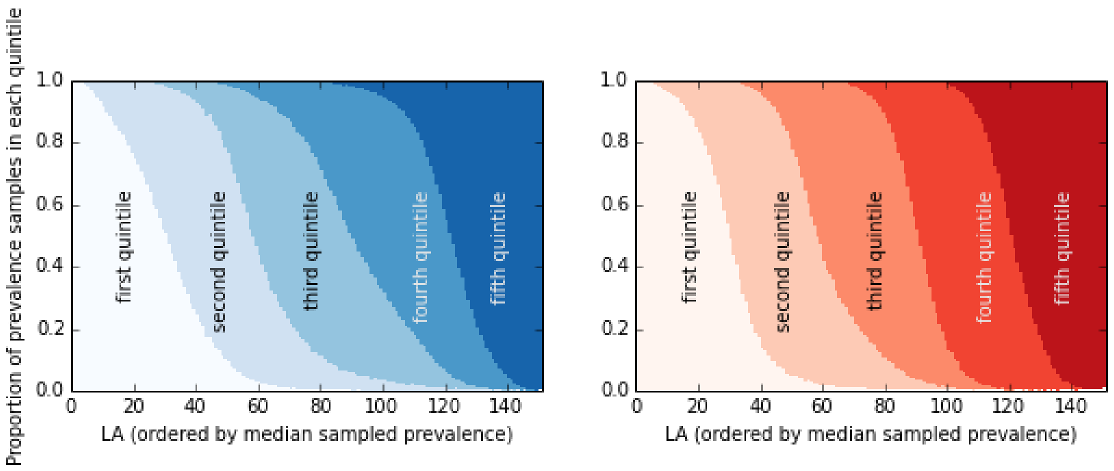

Figure 11: For each LA (horizontal axis), this plot shows how often samples placed the authority in the first, second, ..., fifth quintile for prevalence in men (left) and women (right).

```
ax2.annotate('fourth quintile', [110, 0.6], rotation = 'vertical', color='0.9')
ax2.annotate('fifth quintile', [135, 0.6], rotation = 'vertical', color='0.9')

ax2.set_xlim([0,151])
ax2.set_ylim([0,1])
ax2.set_xlabel('LA (ordered by median sampled prevalence)')
#ax2.set_title('Sexually active women, 15-24 years')

# how many quintiles are occupied >5% of the time?
#howmany = [sum(sizes[i] >= 0.05*n_sample) for i in range(151)]
#print {x:howmany.count(x)/151. for x in howmany}
```

```
Out[17]: <matplotlib.text.Text at 0x1292fb990>
```

This time one column represents one LA, ordered by median sampled prevalence (lowest to highest). Each column is filled according to how many times out of 10000 samples the LA fell into the lowest, second, third, fourth or highest quintile for prevalence. (Adjust the first line of this code block to choose the number of quantiles used.) Samples for the lowest-and highest-prevalence LAs are almost always in the lowest and highest quintiles, respectively, whilst LAs with prevalence estimates in the middle of the range are more likely to be found in two or sometimes three quintiles. There is again a clear order of prevalence which is generally preserved regardless of the particular sampled model parameters.

1.4 Prevalence and incidence

Finally, we plot incidence in each sex against prevalence in the other to examine the effect of infection levels in men on the rate of new infections in women, and vice versa.

```
In [37]: # Figure 10
#plt.rc("savefig", dpi=2300) # for high-resolution version
plt.rc("savefig", dpi=80)

fig = plt.figure(figsize = (5,5), dpi=2300)
ax1 = fig.add_subplot(111)

# add to plot
ax1.plot(percentile(prev_m_la, 50, 0), percentile(inc_f_la, 50, 0), '.', color='#F98400')
ax1.plot(percentile(prev_f_la, 50, 0), percentile(inc_m_la, 50, 0), '.', color='#00A08A')
plt_ppc(ax1, prev_m_la, inc_f_la, 0, 95, '#F98400', alpha=0.15)
plt_ppc(ax1, prev_f_la, inc_m_la, 0, 95, '#00A08A', alpha=0.15)
```

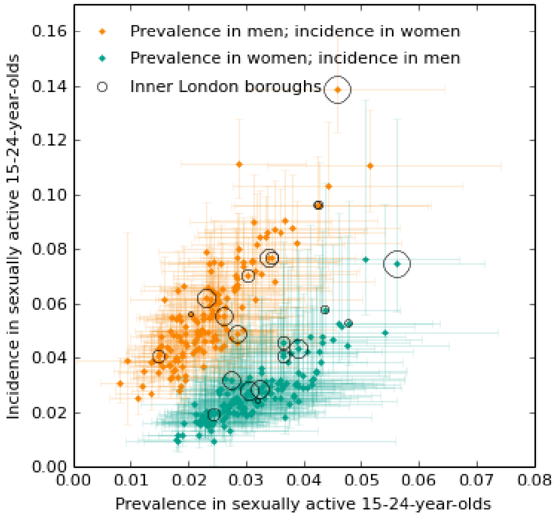

Figure 12: Sampled incidence in each sex against prevalence in the other. Markers and error bars indicate median samples and central 95% credible intervals. Hollow circles highlight inner London boroughs, with circle area proportional to the percentage of the 16-44-year-old population estimated to be MSM.

```
# add inner London boroughs
inn_london_names = ['Lambeth', 'Southwark', 'Lewisham', 'Camden', 'Islington', 'Tower Hamlets',
                    'Hackney', 'Haringey', 'Newham', 'Westminster' 'Kensington and Chelsea',
                    'Hammersmith and Fulham', 'Wandsworth']
inn_london_prop_msm = [15.6, 11.1, 4.9, 11.2, 10.8, 7.7, 7.6, 4.3, 2.9, 9.8, 10.7, 7.5, 4.2]
for i in xrange(len(inn_london_names)):
        interest = where((alldata.la == inn_london_names[i]))
        ax1.plot(percentile(prev_m_la[:,interest], 50, 0)[0],
                 percentile(inc_f_la[:,interest], 50, 0)[0],
                 'o', markerfacecolor='None', c='k',
                 markersize = inn_london_prop_msm[i]
                 )
        ax1.plot(percentile(prev_f_la[:,interest], 50, 0)[0],
                 percentile(inc_m_la[:,interest], 50, 0)[0],
                 'o', markerfacecolor='None', c='k',
                 markersize = inn_london_prop_msm[i]
                 )


ax1.set_xlim([0,0.08]); ax1.set_ylim([0,0.17])

ax1.set_xlabel('Prevalence in sexually active 15-24-year-olds');
ax1.set_ylabel('Incidence in sexually active 15-24-year-olds');

ax1.plot(0.005, 0.16, '.', c='#F98400')
ax1.text(0.01, 0.16, 'Prevalence in men; incidence in women', va='center')
ax1.plot(0.005, 0.15, '.', c='#00A08A')
ax1.text(0.01, 0.15, 'Prevalence in women; incidence in men', va='center')
ax1.plot(0.005, 0.14, 'o', markerfacecolor='None', c='k') # this line can be used to add 'x' markers for the London Bor
ax1.text(0.01, 0.14, 'Inner London boroughs', va='center')
```

```
Out[37]: <matplotlib.text.Text at 0x130f386d0>
```

Orange indicates the relationship between prevalence in men and incidence in women, and green shows the relationship between prevalence in women and incidence in men. Hollow circles highlight inner London boroughs, with circle area proportional to the percentage of the male population aged 16-44 years estimated to be MSM (Ruf *et al.*, *Int J. STD AIDS* **22**:25-29; 2011).

An natural question is why some LAs have higher incidence and prevalence than others. One possibility is that higher screening rates in some areas lower prevalence and incidence. To investigate this, we plot

incidence against screening in men and women:

```
In [38]: # Figure 11

         fig = plt.figure(figsize = (10,10))
         ax1 = fig.add_subplot(221)
         ax2 = fig.add_subplot(222)
         ax3 = fig.add_subplot(223)
         ax4 = fig.add_subplot(224)

         plt_ppc(ax1, scr_m_la, inc_m_la, 0, 95, 'b', alpha=0.2)
         ax1.plot(percentile(scr_m_la,50,axis=0), percentile(inc_m_la,50,axis=0), '.b')
         ax1.set_xlabel('Screening in men'); ax1.set_ylabel('Incidence in men')

         plt_ppc(ax2, scr_f_la, inc_m_la, 0, 95, '#00A08A', alpha=0.2)
         ax2.plot(percentile(scr_f_la,50,axis=0), percentile(inc_m_la,50,axis=0), '.', c='#00A08A')
         ax2.set_xlabel('Screening in women'); ax2.set_ylabel('Incidence in men')

         plt_ppc(ax3, scr_m_la, inc_f_la, 0, 95, '#F98400', alpha=0.2)
         ax3.plot(percentile(scr_m_la,50,axis=0), percentile(inc_f_la,50,axis=0), '.', c='#F98400')
         ax3.set_xlabel('Screening in men'); ax3.set_ylabel('Incidence in women')

         plt_ppc(ax4, scr_f_la, inc_f_la, 0, 95, 'r', alpha=0.2)
         ax4.plot(percentile(scr_f_la,50,axis=0), percentile(inc_f_la,50,axis=0), '.', c='r')
         ax4.set_xlabel('Screening in women'); ax4.set_ylabel('Incidence in women')

Out[38]: <matplotlib.text.Text at 0x13110d650>
```

```
In [39]: # Figure 12

         # examine the Spearman correlation by sample
         spearman = empty([n_sample,4])

         for i in xrange(shape(pos_m_la)[0]):
             spearman[i,0] = stats.spearmanr(scr_m_la[i], inc_m_la[i])[0]
             spearman[i,1] = stats.spearmanr(scr_f_la[i], inc_m_la[i])[0]
             spearman[i,2] = stats.spearmanr(scr_m_la[i], inc_f_la[i])[0]
             spearman[i,3] = stats.spearmanr(scr_f_la[i], inc_f_la[i])[0]

         mpl.rcParams['axes.color_cycle'] = ['b','#00A08A','#F98400','r']
         h=plt.hist(spearman, 20, histtype='step', )
         plt.xlabel('Spearman correlation coefficient')
         plt.ylabel('Frequency')

Out[39]: <matplotlib.text.Text at 0x131300e90>
```

(Colours correspond to marker colours in the plot above.) In fact, the positive correlations show that areas with more screening tend to have higher incidence.

We also examine the relationship with prevalence:

```
In [40]: # Figure 13

         fig = plt.figure(figsize = (10,10))
         ax1 = fig.add_subplot(221)
         ax2 = fig.add_subplot(222)
         ax3 = fig.add_subplot(223)
         ax4 = fig.add_subplot(224)

         plt_ppc(ax1, scr_m_la, prev_m_la, 0, 95, 'b', alpha=0.2)
         ax1.plot(percentile(scr_m_la,50,axis=0), percentile(prev_m_la,50,axis=0), '.b')
         ax1.set_xlabel('Screening in men'); ax1.set_ylabel('Prevalence in men')

         plt_ppc(ax2, scr_f_la, prev_m_la, 0, 95, '#00A08A', alpha=0.2)
         ax2.plot(percentile(scr_f_la,50,axis=0), percentile(prev_m_la,50,axis=0), '.', c='#00A08A')
         ax2.set_xlabel('Screening in women'); ax2.set_ylabel('Prevalence in men')

         plt_ppc(ax3, scr_m_la, prev_f_la, 0, 95, '#F98400', alpha=0.2)
         ax3.plot(percentile(scr_m_la,50,axis=0), percentile(prev_f_la,50,axis=0), '.', c='#F98400')
         ax3.set_xlabel('Screening in men'); ax3.set_ylabel('Prevalence in women')
```

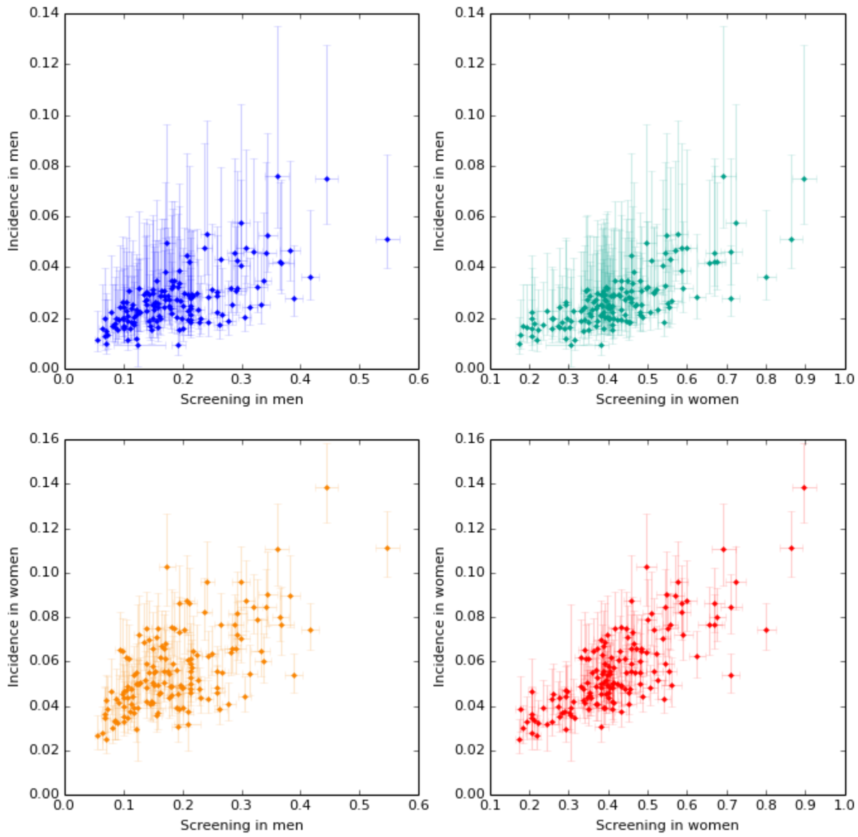

Figure 13: Sampled incidence vs. screening rate. Markers and error bars indicate median samples and central 95% credible intervals. Top-left: incidence in men vs. screening in men; top-right: incidence in men vs. screening in women; bottom-left: incidence in women vs. screening in men; bottom-right: incidence in women vs. screening in women.

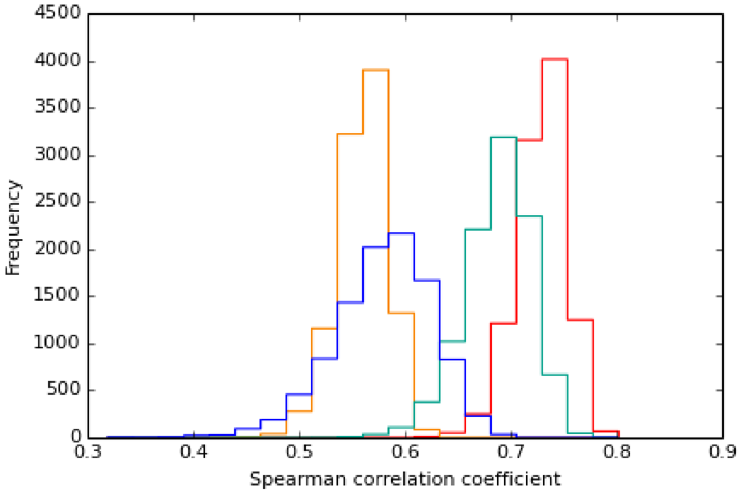

Figure 14: Spearman correlations between screening and incidence, at each of 10000 samples. Blue: incidence in men vs. screening in men; green: incidence in men vs. screening in women; orange: incidence in women vs. screening in men; red: incidence in women vs. screening in women.

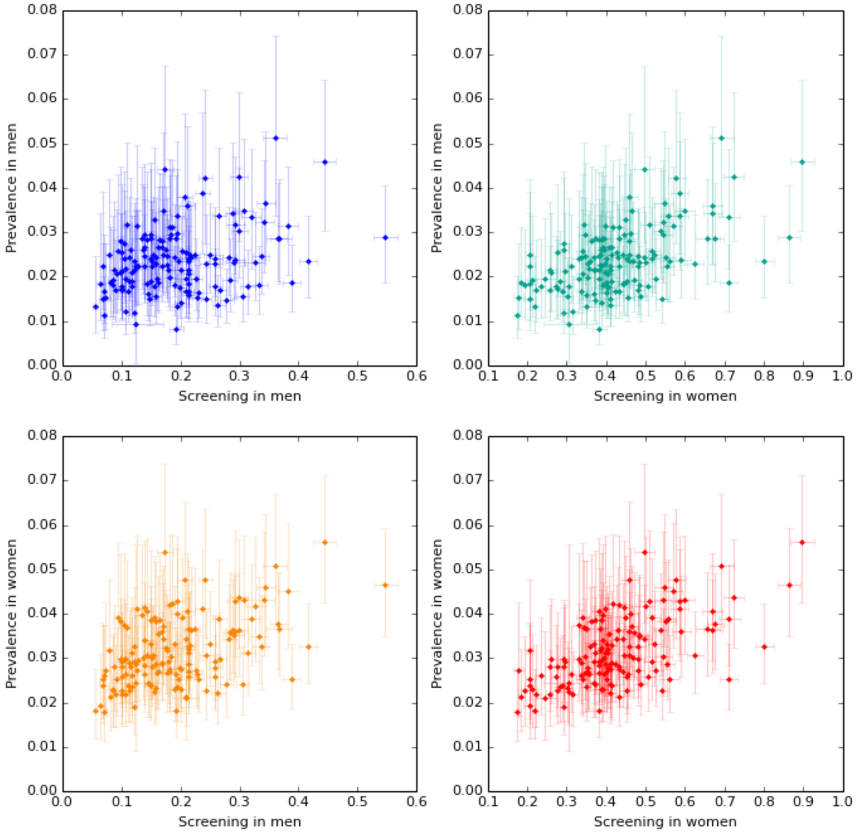

Figure 15: Sampled prevalence vs. screening rate. Markers and error bars indicate median samples and central 95% credible intervals. Top-left: prevalence in men vs. screening in men; top-right: prevalence in men vs. screening in women; bottom-left: prevalence in women vs. screening in men; bottom-right: prevalence in women vs. screening in women.

```
        plt_ppc(ax4, scr_f_la, prev_f_la, 0, 95, 'r', alpha=0.2)
        ax4.plot(percentile(scr_f_la,50,axis=0), percentile(prev_f_la,50,axis=0), '.', c='r')
        ax4.set_xlabel('Screening in women'); ax4.set_ylabel('Prevalence in women')
```

```
Out[40]: <matplotlib.text.Text at 0x13186a710>
```

```
In [41]: # Figure 14
         # examine the Spearman correlation by sample
         spearman = empty([n_sample,4])

         for i in xrange(shape(pos_m_la)[0]):
             spearman[i,0] = stats.spearmanr(scr_m_la[i], prev_m_la[i])[0]
             spearman[i,1] = stats.spearmanr(scr_f_la[i], prev_m_la[i])[0]
             spearman[i,2] = stats.spearmanr(scr_m_la[i], prev_f_la[i])[0]
             spearman[i,3] = stats.spearmanr(scr_f_la[i], prev_f_la[i])[0]

         h=plt.hist(spearman, 20, histtype='step', )
         plt.xlabel('Spearman correlation coefficient')
         plt.ylabel('Frequency')
```

```
Out[41]: <matplotlib.text.Text at 0x131a65c10>
```

Prevalence is also generally higher in areas with more screening.

What about the relationship between screening in men vs. women, and screening in men vs. women?

```
In [42]: # Figure 15
```

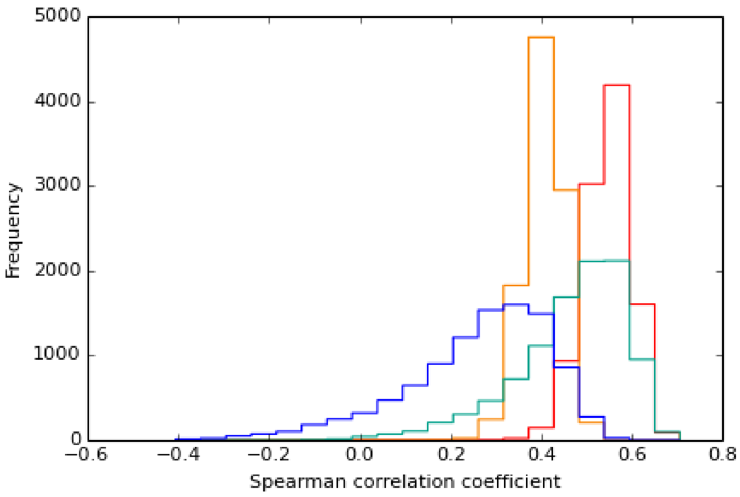

Figure 16: Spearman correlations between screening and prevalence, at each of 10000 samples. Blue: prevalence in men vs. screening in men; green: prevalence in men vs. screening in women; orange: prevalence in women vs. screening in men; red: prevalence in women vs. screening in women.

```
fig = plt.figure(figsize = (10,5))

ax1 = fig.add_subplot(121)
plt_ppc(ax1, prev_m_la, prev_f_la, 0, 95, 'k', alpha=0.15)
p = ax1.plot(percentile(prev_m_la,50,0), percentile(prev_f_la,50,0), '.', color='k')
ax1.set_xlim(0,0.1)
ax1.set_ylim(0,0.1)
ax1.set_xlabel('Prevalence in men')
ax1.set_ylabel('Prevalence in women')

ax2 = fig.add_subplot(122)
plt_ppc(ax2, scr_m_la, scr_f_la, 0, 95, 'k', alpha=0.3)
p = ax2.plot(percentile(scr_m_la,50,0), percentile(scr_f_la,50,0), '.', color='k')
ax2.set_xlim(0,1)
ax2.set_ylim(0,1)
ax2.set_xlabel('Screening in men')
ax2.set_ylabel('Screening in women')
```

Out[42]: <matplotlib.text.Text at 0x131e61810>

Prevalence in men and women is positively correlated, because of the incidence-prevalence relationship illustrated above. LAs with more non-symptomatic screening of men also tend to have more screening of women, but all LAs have more screening in women than men.

In [ ]:

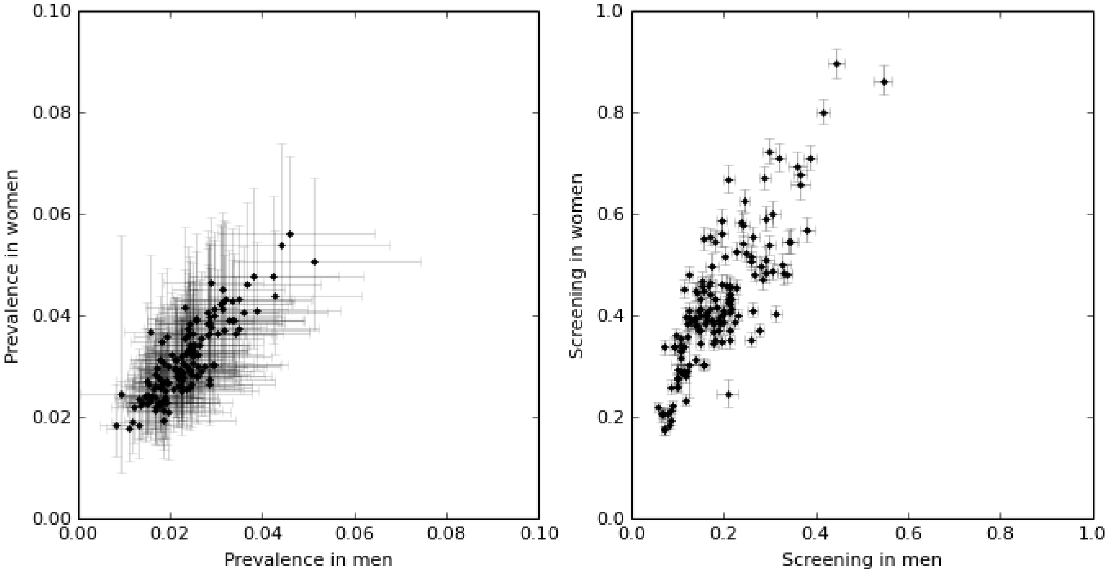

Figure 17: Correlation in local prevalence (left) and screening (right) in men vs. women. Markers and error bars indicate median samples and central 95% credible intervals.
